# Organic persistent room temperature phosphorescence enabled by carbazole impurity

**DOI:** 10.3389/fchem.2022.1008658

**Published:** 2023-01-06

**Authors:** Alexander C. Brannan, Nguyen Le Phuoc, Mikko Linnolahti, Alexander S. Romanov

**Affiliations:** ^1^ Department of Chemistry, The University of Manchester, Manchester, United Kingdom; ^2^ Department of Chemistry, University of Eastern Finland, Joensuu, Finland

**Keywords:** organic, persistent room-temperature phosphorescence, afterglow, carbazole, charge transfer

## Abstract

The molecular design of metal-free organic phosphors is essential for realizing persistent room-temperature phosphorescence (pRTP) despite its spin-forbidden nature. A series of halobenzonitrile–carbazoles has been prepared following a one-pot nucleophilic substitution protocol involving commercially available and laboratory-synthesized carbazoles. We demonstrate how halo- and cyano-substituents affect the molecular geometry in the crystal lattice, resulting in tilt and/or twist of the carbazole with respect to the phenyl moiety. Compounds obtained from the commercially available carbazole result in efficient pRTP of organic phosphors with a high quantum yield of up to 22% and a long excited state lifetime of up to 0.22 s. Compounds obtained from the laboratory-synthesized carbazole exhibit thermally activated delayed fluorescence with an excited state lifetime in the millisecond range. In-depth photophysical studies reveal that luminescence originates from the mixed locally excited state (^3^LE, nπ*)/charge transfer state.

## Introduction

Persistent room-temperature phosphorescence (pRTP) denotes phosphorescent emission with an excited state lifetime exceeding 0.1 s with clearly observable afterglow after switching off the excitation light source at room temperature (Bhattacharjee and Hirata, 2020). This behavior is highly desirable for implementation in various technologies, such as time-resolved bioimaging and biosensing applications, safety signage, lighting, and anti-counterfeiting (Maldiney et al., 2011; An et al., 2015; Cai et al., 2017; Su et al., 2018; Wu et al., 2021; Zhang et al., 2022). A record-setting afterglow lasting 10 h has been reported for inorganic salts (CaAlO_4_ and others) containing Eu, Dy, or Nd rare-earth dopants, although it affected the cost of the final product, raising sustainability concerns while limiting the processability (Aoki et al., 1996; Lin et al., 2001; Wang et al., 2002). The development of purely organic pRTP materials would be of immense importance to provide an alternative while leveraging the aforementioned problems. However, organic compounds rarely show bright and room-temperature phosphorescence due to spin-forbidden triplet-to-singlet transitions where non-radiative pathways out-compete the phosphorescence. Several approaches have been demonstrated on how to overcome this problem by modifying the organic materials with heavy atom substituents (for instance, bromide or iodide) (Cai et al., 2017; Yuan et al., 2019), molecular design and crystal packing engineering of the organic emitter based on the intermolecular contacts (Li et al., 2019; Ma et al., 2019; Wang et al., 2020; Zhao et al., 2020), or H-aggregates (Gu et al., 2019). Heavy atoms, such as bromide and iodide, facilitate the intersystem-crossing due to large spin–orbit coupling coefficients (H_SO_), enabling otherwise spin-forbidden room-temperature phosphorescence. Even isotopic H/D-exchange on an organic emitter, such as coronene, enhances the pRTP lifetime from 6 s and PLQY 4% up to 23 s and PLQY 12% for the perdeuterated coronene (Kropp and Dawson, 1967). However, the most efficient and bright pRTP materials would involve a combination of these approaches, where Yuan et al. reported a series of bromophenyl–carbazole materials with impressive excited state lifetimes exceeding 0.3 s and high photoluminescence quantum yields (PLQY up to 20%) (Li et al., 2019; Yuan et al., 2019).

**CHART 1:**
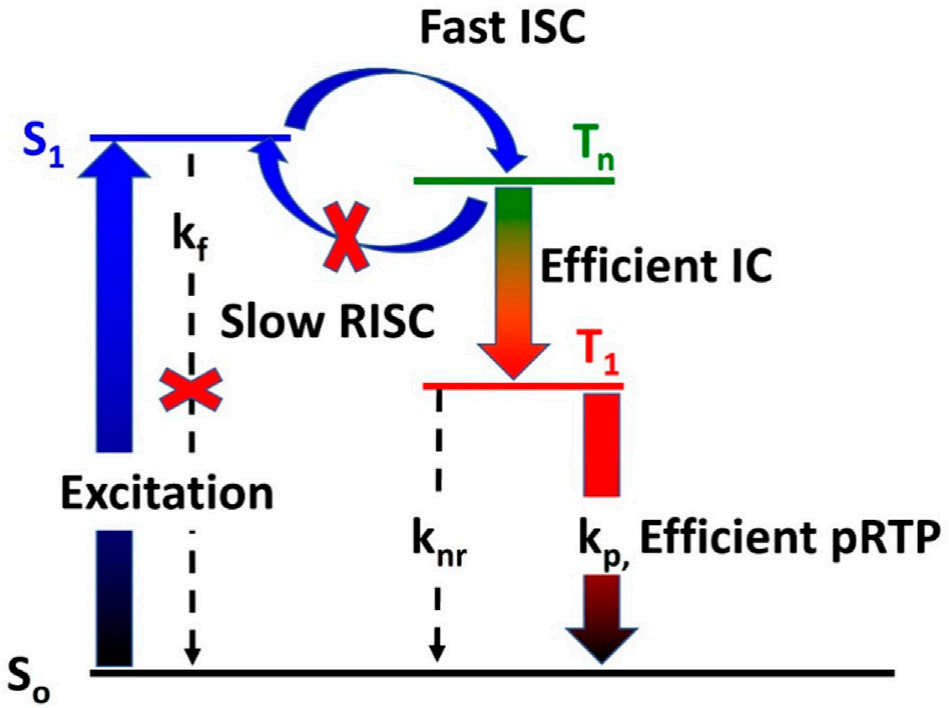
General scheme to enable efficient pRTP in organic materials, where RISC, reverse intersystem crossing; ISC, intersystem crossing; IC, internal conversion or triplet–triplet transition (Zhao et al., 2019).

The pRTP materials have to qualify a key requirement—a large energy gap (>0.3 eV) between the charge transfer manifold of the singlet and triplet states (CT, ππ*) and the triplet locally excited state (^3^LE, nπ*)—to suppress the reverse intersystem crossing (RISC) and thermally activated delayed fluorescence radiative pathway (Chart 1). Therefore, organic materials must be carefully designed to enable efficient intersystem crossing (ISC) *via* spin–vibronic coupling (H_SOC_) (Etherington et al., 2016) and satisfy El-Sayed’s rule, which denotes ISC between states of different configurations (<^1^CT |H_SOC_ |^3^LE>) is more efficient compared to that of states having the same configuration (<^1^CT |H_SOC_ |^3^CT>) (El-Sayed, 1963; Yang et al., 2016). However, a large family of the pRTP materials contain a heavy atom that not only facilitates the desired ISC but also simultaneously contributes to high phosphorescence rate k_P_ and non-radiative rate k_nr_, resulting in either a short excited state lifetime or quenching of the luminescence. Tang et al. (Zhao et al., 2019) suggested bypassing this problem by using the triplet–triplet energy transfer process (TTET) to convert the high-energy triplet excitons (T_n_, Chart 1) into low-energy ones by the Dexter energy transfer mechanism (Tanner et al., 2018). Following this strategy, Tang et al. demonstrated highly efficient (PLQY 41%) and persistent phosphors (0.54 s) based on intramolecular triplet–triplet transitions in carbazole-decorated (bromo)dibenzofuran or (bromo)dibenzothiophene (Zhao et al., 2019). The intermolecular TTET process was applied by Bryce, Chi, and Zhang et al. in various substituted ketone and sulphone organic materials to demonstrate pRTP materials with respectable PLQY of 5% and remarkably long excited state lifetime of 0.28 s, achieved by careful tuning of the energy of the excited states having different configurations (i.e., nπ* and ππ*) (Yang et al., 2016). The design of pRTP organic materials operating *via* the intermolecular TTET process is highly challenging, although a recent report by Hirata demonstrates bright and heavy atom-free pRTP materials (PLQY 46% and lifetime 1.4 s) triggered by the hidden long phosphorescent antenna (Bhattacharjee and Hirata, 2020).

The molecular design is important to achieve efficient pRTP materials and requires in-depth investigation of the molecular conformation where the nature and steric requirements of the substituents in the organic donor–acceptor pRTP materials enable the control of the molecular conformation. For instance, donor–acceptor materials based on the N-substituted phenothiazine donors and dibenzosulfone core as acceptor result in a significant pyramidalization of the donor N-atom (the sum of the bond angles is less than 348°) (Chen et al., 2018). Increasing the size of the substituent in the ortho position of the phenothiazine donor (R = H < Me < iPr) results in the gradual change from nearly co-planar to near-orthogonal donor–acceptor orientation (torsion angle, 57–62°) (Ward et al., 2018) and efficient room temperature phosphorescence instead of the thermally activated delayed fluorescence (TADF) (Ward et al., 2016). It is found that the RTP property becomes more pronounced with an increase in the twist angle between the donor and acceptor ligands (Ward et al., 2016).

**CHART 2:**
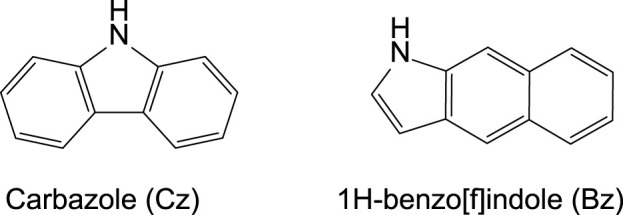
Molecular structures of the carbazole (Cz) and commonly present admixture 1H-benzo[f]indole (Bz) in the commercial samples of the carbazole.

The pRTP phosphors with carbazole (Cz, Chart 2) as a donor moiety represent a well-studied class of materials that benefit from the low-energy (nπ*) triplet excited state located on the Cz. Ortho-substitution of the carbazoles enables a control between nearly twisted, tilted, and/or fully twisted conformation in organic emitters or organometallic compounds (Brannan and Lee, 2020; Gu et al., 2022). However, there are no reports of the pRTP materials where the N-atom of the carbazole moiety may experience a pyramidalization due to the rigidity of the carbazole ligand. In addition, Liu et al. reported that the minor admixture of the 1H-benzo [f]indole (Bz) in commercially available carbazole (Chart 2) induces pRTP luminescence in numerous Cz-based materials (Chen et al., 2021). This discovery initiated research activities toward the doping of the carbazole-containing compounds with Bz to enable and intensify pRTP luminescence (Qian et al., 2022). Here, we demonstrate that the Bz admixture in the commercial samples of the carbazole enables the pRTP luminescence in the halobenzonitrile–carbazole materials. We show that tilted and twisted carbazole ligand conformation defines the network of intermolecular contacts in the crystal lattice that enable and/or intensify the efficiency of the pRTP luminescence enabled by the Bz admixture.

## Results and discussion

**FIGURE 1:**
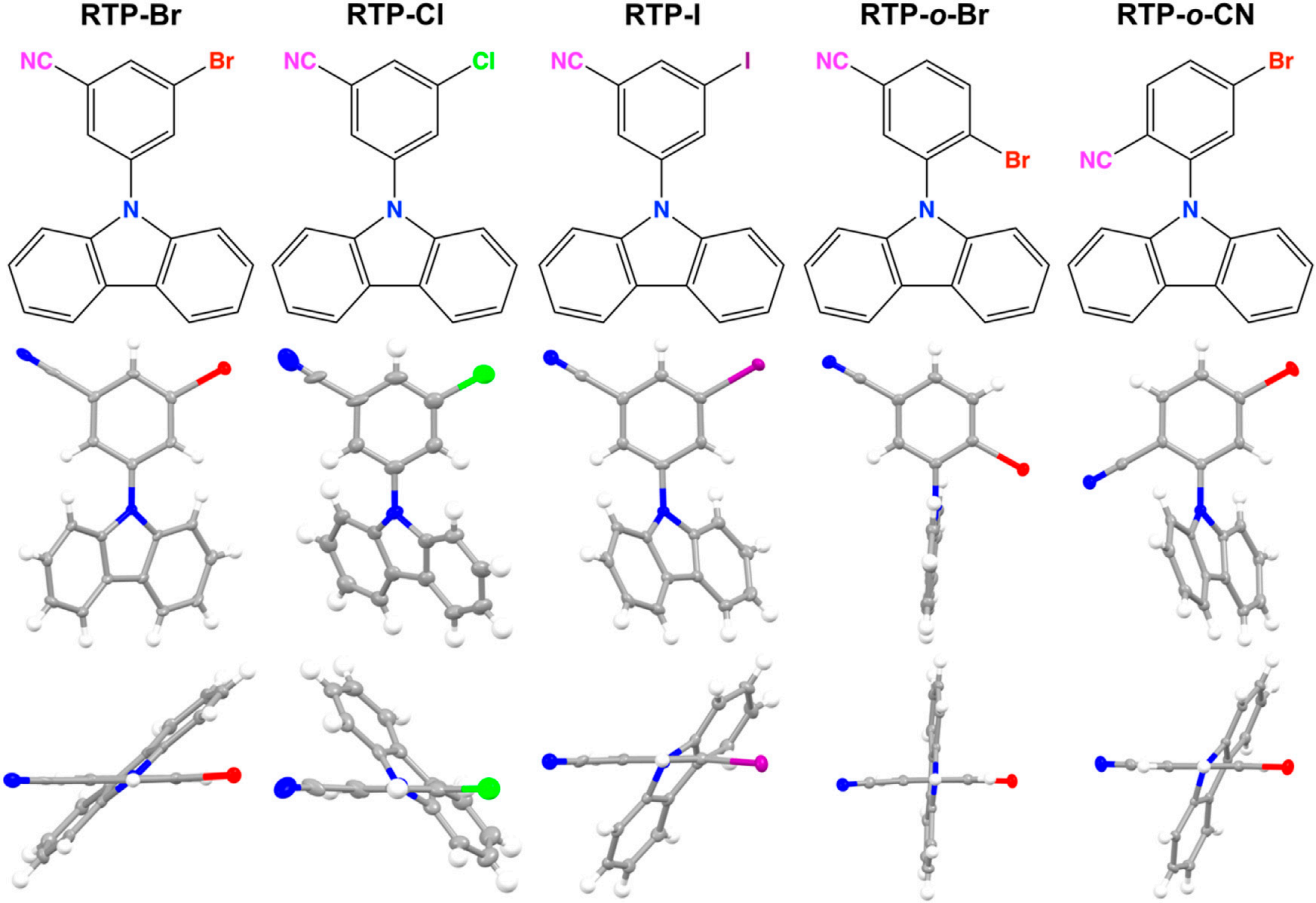
Molecular structures and single-crystal X-ray crystal structures of the pRTP materials **RTP-Br**, **RTP-Cl**, **RTP-I**, **RTP-*o*-Br**, and **RTP-*o*-CN** (left to right with front and side views).

A series of the pRTP materials (Figure 1) are prepared in a two-step procedure by treatment of the carbazole with sodium hydride in dry DMF, followed by heating with the respective fluorinated halobenzonitrile to form the products by nucleophilic substitution in high yields. The compounds are off-white solids stable in the air, which were fully characterized by NMR, X-ray crystallography, and high-resolution mass spectroscopy with the synthetic details collected in the Supplementary Materials. The synthesis of the RTB-Br and RTP-o-Br was repeated with the carbazole synthesized in the laboratory (Freeman et al., 2005) to demonstrate the influence of the 1H-benzo[f]indole admixture in the commercial samples of the carbazole.

### X-ray crystallography

The crystal structures for all complexes were determined by single crystal X-ray diffraction to reveal the exact conformation of the molecules by introducing a twist angle α (C3-C4-N2-C8), a tilt angle β (C4–N2-Centroid Cg1), and the pyramidalization of the carbazole N atom (represented by the angle sum γ around N2; *γ* = C4–N2–C8 + C4–N2–C19 + C8–N2–C19, Figure 1; Table 1). The bond length between the phenyl and carbazole moieties C4–N2 varies in the range of 1.408(5)–1.425(4)Å, while it is shorter for the brightest pRTP materials (**RTP-Br** and **RTP-o-CN**) and longer for the rest and less efficient pRTP compounds. All compounds show a twisted geometry between carbazole and phenyl moiety along the N_Cz_-C_Ph_ bond (twist angle α, Table 1). The torsion angle increases in the range **RTP-Cl** < **RTP-I** < **RTP-Br** < **RTP-*o*-CN** < **RTP-*o*-Br**, indicating a nearly twisted geometry for the chloride and iodide derivatives and almost fully twisted for the **RTP-*o*-Br** compound. The nearly twisted **RTP-Cl** and **RTP-I**, and fully twisted **RTP-*o*-Br** demonstrate the lowest PLQY value of 5%. The fact that the high pRTP PLQY value of *ca.* 20% is measured for **RTP-Br** and **RTP-*o*-CN** suggests that the most efficient materials should have a twist angle in the range of ca. 44°–52°. Notably, poorly emissive materials with the near co-planar **RTP-Cl** and **RTP-I** geometry show considerable titling of the carbazole moiety and pyramidalization of the N-atom (angle β and *γ*, Table 1), whereas the fully twisted and poorly emissive **RTP-*o*-Br** shows neither tilt nor pyramidalization of the carbazole N-atom. It is likely that the minor tilt and twist of the carbazole ligand observed for the **RTP-Br** and **RTP-*o*-CN**, which fit precisely within the range of two extreme cases of nearly twisted (**RTP-Cl** and **RTP-I**) and fully twisted **RTP-*o*-Br** compounds, are advantageous to realize the bright pRTP luminescence (Table 1).

**TABLE 1:** General numbering scheme, selected bond lengths [Å], and angles [°] for all compounds and unit cell parameters for the **RTP-Br and RTP-*o*-Br materials obtained from the commercial and laboratory synthesized carbazole**

Compound	C4–N2	Torsion angle (*α,* °) C3-C4-N2-C8	Tilt angle (*β,* °) Cg1-N2-C2	Sum of the angles around N2, (*γ,* °)
RTP-Br	1.408 (5)	43.6 (5)	174.44)	359.7 (3)
RTP-Cl	1.422 (5)	35.0 (6)	162.5 (3)	356.0 (3)
RTP-I	1.425 (4)	37.3 (4)	160.4 (2)	355.1 (4)
RTP-*o*-CN	1.416 (3)	51.7 (3)	164.5 (3)	356.5 (4)
RTP-*o*-Br	1.411 (4)	88.0 (4)	178.1 (3)	359.9 (3)

Unit cell parameters	com-RTP-Br	lab-RTP-Br	com-RTP-*o*-Br	lab-RTP-*o*-Br
*a*, Å	3.9322 (2)	3.9217 (2)	15.9502 (7)	15.9510 (4)
*b*, Å	10.0657 (5)	10.0473 (3)	12.5562 (4)	12.56033)
*c*, Å	18.0728 (8)	18.1502 (6)	15.9434 (8)	15.9556 (4)
*α*,°	88.000 (4)	88.050 (3)	–	–
*β*,°	84.762 (3)	84.531 (4)	113.440 (5)	113.396 (3)
*γ*,°	86.165 (4)	85.9584)	–	–
*V,* (Å^3^)	710.45 (6)	709.86 (5)	2,929.5 (2)	2,933.87 (14)

Here, Cg1 is the centroid of the C8-C13-C14-C19-N2 carbazole plane; **com,** sample obtained from the commercially available carbazole; **lab,** sample obtained from the carbazole prepared in the laboratory.
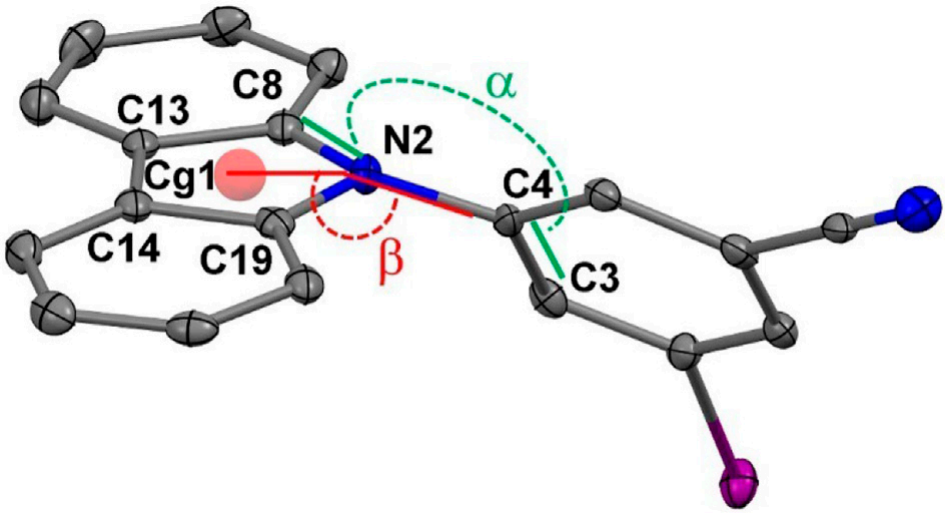

Recently, Li et al. reported that strong intermolecular forces in the unit cell for analogous bromophenyl–carbazole compounds enhanced the quantum yields and are imperative for efficient pRTP (Li et al., 2019). This is due to suppressed non-radiative pathways and better protection from quenchers such as atmospheric oxygen. Analogous methoxyphenyl carbazoles were shown to increase the number of strong intermolecular forces in the crystal lattice, with C−H···O interaction distances ranging from 2.755(3) to 3.348(4) Å. Our most efficient materials, **RTP-Br** and **RTP-*o*-CN**, exhibit short bifurcated intermolecular hydrogen bonds between C−H_Ph_···NC_cyano_ and C−H_Cz_···NC_cyano_ in the range of 2.587(5)–2.739(5) Å (Supplementary Figures S10, S12, S14 and S16). These hydrogen bond lengths are shorter than those for the methoxy derivative, indicating stronger intermolecular forces for **RTP-Br** and **RTP-*o*-CN**, which also contains weak C–Br···π(carbazole) interaction 3.347(7) Å. The analysis of the intermolecular contacts for the least efficient **RTP-Cl** and **RTP-I** shows a very different 3D network of weak C−H···halide and C−H···π hydrogen bonds, resulting in a distortion of the phenyl moiety plane and significant tilting of the carbazole ligand outside the optimal range of the aforementioned twist and tilt angles (α and β, Table 1). Therefore, we observe that strong bifurcated intermolecular hydrogen bonds for **RTP-Br** and **RTP-*o*-CN** in the crystal correlates well with the most pronounced pRTP emission (PLQY over 20%).

We analyzed the single-crystal structure of the best pRTP materials (**RTP-Br** and **RTP-*o*-Br**) obtained from the laboratory-made carbazole to demonstrate that both compounds crystallize with only minor deviations in the unit cell parameters (Table 1) and intermolecular contact network identical to the crystals containing the Bz admixture. This fact indicates that the presence of the Bz impurity in the samples is insufficient to influence the molecular conformation and intermolecular contacts of pRTP materials; however, those are imperative to enable and enhance the pRTP luminescence.

### Photophysical properties

**FIGURE 2:**
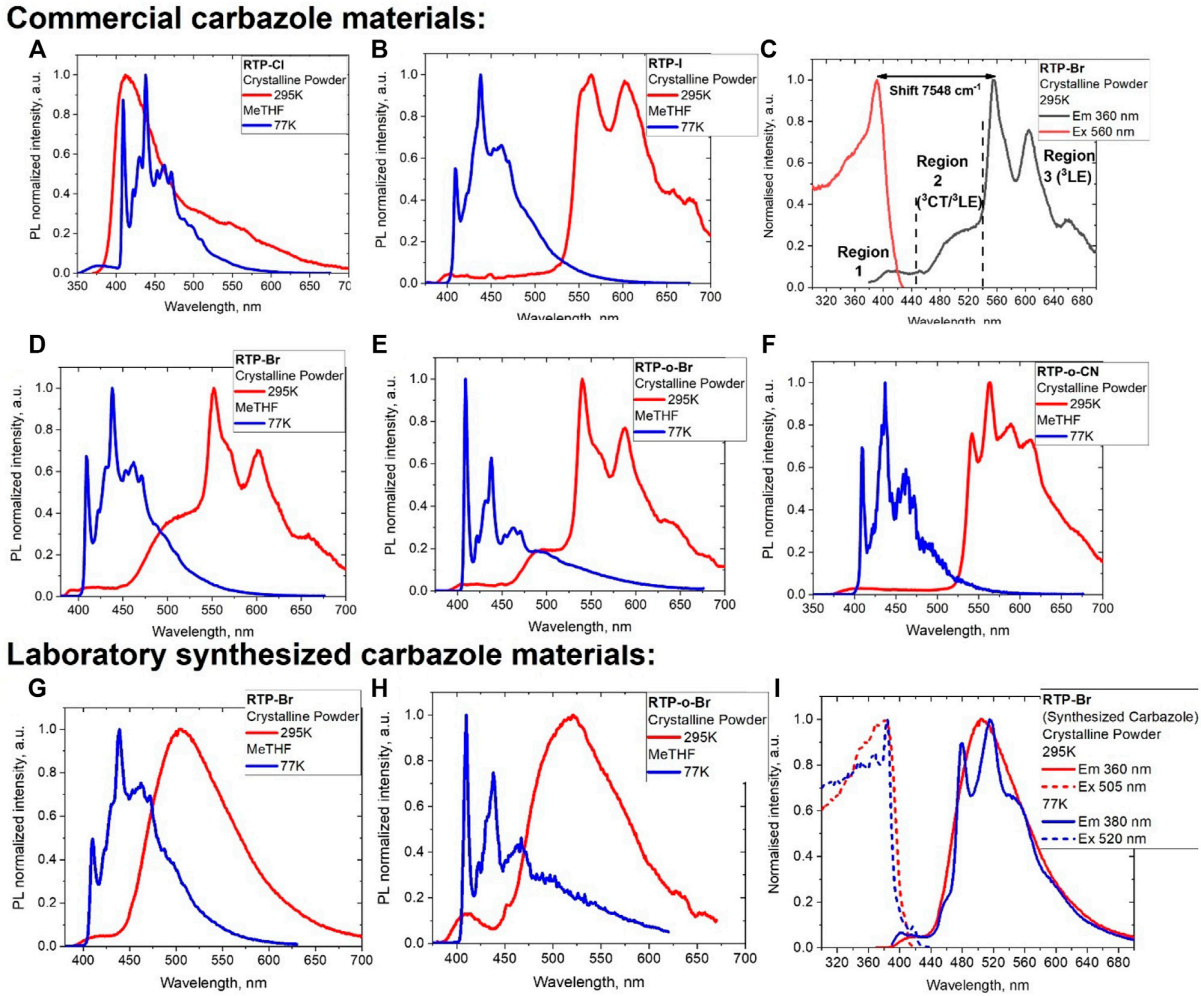
Photoluminescent spectra in crystalline powder at 295 K (red, excited at 360 nm) and frozen MeTHF solution at 77 K (blue, excited at 320 nm) for **(A) RTP-Cl**; **(B) RTP-I**; **(C) RTP-Br** emission (360 nm) and excitation (560 nm) profiles with three luminescence regions: fluorescence (region 1), phosphorescence (region 2), and room temperature persistent phosphorescence (region 3); **(D) RTP-Br**; **(E) RTP-*o*-Br**; and **(F) RTP-o-CN**. Photoluminescent spectra in crystalline powder at 295 K (red, excited at 360 nm) and frozen MeTHF solution at 77 K (blue, excited at 320 nm) for materials obtained from the laboratory synthesized carbazole: **(G) RTP-Br**, **(H) RTP-*o*-Br**, and **(I)** emission (360 nm) and excitation (560 nm) profiles for **RTP-Br**.

We measured the photoluminescence behavior for all pRTP materials in the crystalline state at 295 K and frozen MeTHF solution at 77 K, as shown in Figure 2, while luminescence data are collected in Table 2. We first discuss the samples obtained from the commercially available Cz. We can identify three emissive regions, which we measure and explain based on the example of the **RTP-Br** (Figure 2F). The first emissive region is usually weak and observed as a broad profile in the range of 380–440 nm (fluorescence from a singlet state), and the second emissive region (450–530 nm) can be weak or second in intensity while being featureless, indicating the luminescence occurs from the charge transfer between the carbazole moiety to a 3-bromo-5-cyanophenyl moiety. The third emissive region (540–700 nm) corresponds to the most intense luminescence with clear vibronic progression. We measured a very large energy difference between the excitation and emission maxima (7,544 cm^−1^ as shown in Figure 2C for RTP-Br) which, together with the very long excited state lifetime, enabled us to ascribe luminescence with vibronic progression to room-temperature phosphorescence, which originates due to Bz impurity in line with a previous study (Chen et al., 2021).

**TABLE 2:** Photophysical properties in solid state and MeTHF solution.

	λ_em_ (nm)	τ_500nm_ (ms)	τ_550nm_ (ms)	Φ (%)	λ_em_ (nm)	τ_460nm_ (ms)
Crystalline powder (295 K with Bz admixture)					MeTHF (77 K)	
RTP-Br	552	0.65 (39%)	19.9 (11%)	20	438	40.0 (21%)
		3.51 (31%)	216 (89%)			217 (20%)
		27.9 (30%)				2,434 (59%)
RTP-Cl	413	1.71 (44%)	14.6 (23%)	6	438	744 (17%)
		22.6 (56%)	406 (77%)			4,227 (83%)
RTP-I	564	-	16.9 (78%)	7	438	10.9 (24%)
			163 (22%)			105 (23%)
						1,517 (53%)
RTP-*o*-CN	563	-	106 (41%)	22	437	203 (4%)
			293 (59%)			3,060 (96%)
RTP-*o*-Br	540	4.78 (29%)	56.6 (6%)	6	409	16.3 (6%)
						180 (21%)
		127 (71%)	371 (94%)			670 (73%)
pRTP materials from the laboratory synthesized carbazole at 295 K					MeTHF (77 K)	
RTP-Br	505	0.05 (48%)	–	4	438	42.0 (23%)
		0.3 (35%)				220 (25%)
		1.1 (17%)				2,440 (55%)
RTP-*o*-Br	520	0.002 (50%)	–	3	409	124 (9%)
		0.01 (35%)				
		0.04 (15%)				658 (91%)

**FIGURE 3:**
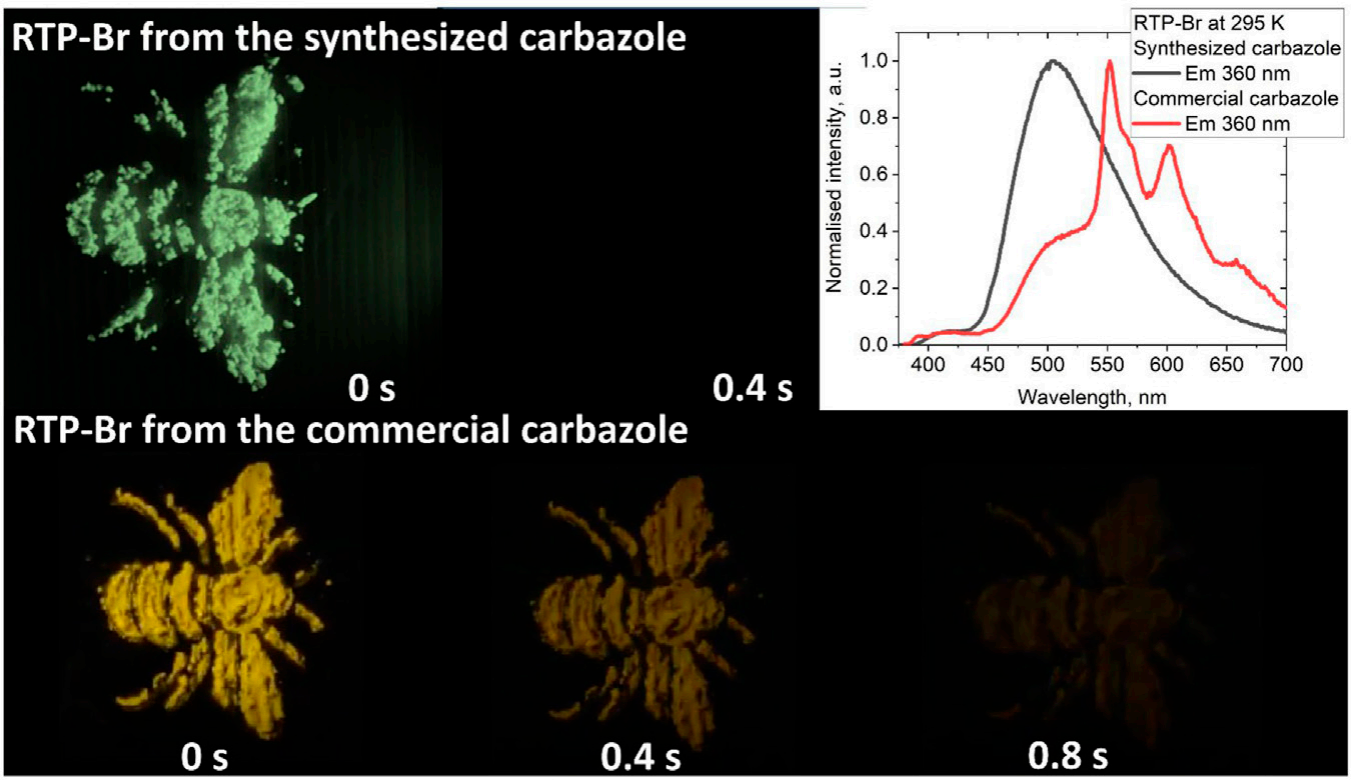
Bottom: complex RTP-Br obtained from the commercial carbazole sample (bottom, shows RTP property). Top: complex RTP-Br obtained from the synthesized carbazole (fast decay of the luminescence). All samples are exposed to 360 nm UV-light with top right panel showing difference in the luminescence profile.

The intensity of the luminescence from the first region gradually decreases from **RTP-Cl > RTP-Br > RTP-I**, which correlates well with an increase in the spin–orbit coupling coefficients for the halides facilitating the ISC process and phosphorescence. For instance, **RTP-Cl** shows the most intense high-energy emission band at 413 nm (Figure 2A) with a long tail up to 700 nm where phosphorescence has a multiexponential decay (1–20 ms), including the pRTP process (0.4 s) with a total PLQY value of 6%. In contrast, **RTP-I** predominantly shows a low-energy well-resolved pRTP luminescence at 564 nm (third region, Figure 2B) with 0.16 s excited state lifetime and 7% PLQY. Unlike the chloride substituent in RTP-Cl, high spin–orbit coupling of the iodide substituent could facilitate the ISC rates and IC. Low PLQY for **RTP-Cl** and **RTP-I** compounds were associated with geometrical distortions such as tilting and twisting of the carbazole moiety, as discussed previously. Bromide derivative **RTP-Br** shows a very weak emission profile from the first region (Figures 2C, D; Figure 3), whereas it shows a dual luminescence: phosphorescence from the CT-state at 500 nm (the second region with excited state lifetime up to 22 ms) and resolved pRTP from the ^3^LE (Cz) state at 552 nm (0.2 s). High PLQY values (up to 20%) for pRTP at room temperature suggest that the short bifurcated intermolecular hydrogen bonds and weak C–Br···π(carbazole) interactions for the **RTP-Br** are imperative to enhance RTP luminescence. Notably Li et al. (2019) and Yuan et al. (2019) reported a compound similar to **RTP-Br** but without the cyano-substituent 9-(3-bromophenyl)-carbazole demonstrating pRTP with lifetimes of 0.24 s but only 7% PLQY. Therefore, installation of the cyano-group at the meta-position resulted in a three-fold increase of the PLQY in **RTP-Br** compared to the reported 9-(3-bromophenyl)-carbazole p-RTP material.

The crystalline powders of ortho-substituted materials **RTP-*o*-CN** and **RTP-*o*-Br** show a very weak luminescence in the first region (Figures 2E, F). The emission profile of **RTP-*o*-Br** is closely similar to that of **RTP-Br**, where the excited state lifetime for the second region (CT luminescence, Figure 2E) is 92 ms, whereas the structured RTP-luminescence shows an exceptionally long lifetime of 0.35 s (region 3, Figure 2E). Changing the position of the bromide from meta to ortho on the phenyl ligand resulted in a two-time longer pRTP, at the sacrifice of the PLQY values, which dropped from 20% to 6% due to the fully twisted conformation of **RTP-*o*-Br**. Finally, we consider the case where we change the position of the cyano-substituent from meta to ortho while keeping the bromide in the desirable meta position **RTP-*o*-CN**. The photoluminescence spectrum of crystalline powder of **RTP-*o*-CN** is similar to the **RTP-I** with a dominant and well-resolved pRTP luminescence at 563 nm and negligible contribution from the first and second regions (Figure 2F). The **RTP-*o*-CN** compound has an averaged excited state lifetime of 0.22 s, which is slightly longer than 0.2 s for **RTP-Br**. While there is no discernible emission component from a triplet CT state, the PLQY value is also the highest in the series, reaching 22%. Such a performance is likely connected with the molecular conformation of the **RTP-*o*-CN** (half-twist and significant tilting of the carbazole moiety), resulting in similar but shorter intermolecular contacts (hydrogen bonds and weak C–Br···π(carbazole) interactions) discussed for the second-best material **RTP-Br**. This further supports the particular intermolecular contact network’s importance in realizing highly efficient RTP luminescence for materials with Bz admixture (Chart 2).

We obtained **RTP-Br** and **RTP-o-Br** compounds from the laboratory-synthesized carbazole to demonstrate the role of the Bz-impurity as an origin of the RTP luminescence. Both compounds show a white-green luminescence (Figures 2G, H; Figure 3) with only two distinct regions overlapping with the first two luminescence regions of **RTP-Br** and **RTP-o-Br** having a Bz admixture (Figure 3). The excited state lifetime for the second region PL increases from the sub-millisecond range to seconds upon cooling to 77 K. The PL profile at 77 K clearly shows a vibronic structure (Figure 2I) which indicates that the luminescence originates from the carbazole-centered locally excited triplet state ^3^LE (Cz) for the most intense second region (Figures 2G,H). Therefore, we ascribe the PL from the second region to the thermally activated delayed luminescence. Notably, the excited state lifetime for **RTP-Br** and **RTP-o-Br** is up to four orders of magnitude shorter than the RTP samples with Bz admixture. The absence of the third region the in PL profile clearly indicates the need for Bz-doping (Chart 2) to enable RTP luminescence, as shown in Figure 5.

We measured the luminescence in frozen 2-methyltetrahydrofuran (MeTHF at 77 K) for all compounds to identify the ^3^LE energy level without the presence of intermolecular contacts. All complexes were excited with 340 nm light and showed a highly resolved emission profile from 400 to 550 nm, which is similar across all compounds. We assign it to a high-energy triplet local excited state of the carbazole ^3^LE (carbazole) at 3.07 eV, which is close to the theoretically calculated energy value for the T1 state, for instance, 3.47 eV for RTP-Br. The difference between the calculated and experimental values for the T1 state [^3^LE (carbazole)] is in the range of 0.4–0.5 eV for all complexes in frozen MeTHF glass. The excited state lifetime for all complexes increased a minimum ten-fold up to 4.2 s, exceeding our equipment’s detection limit. A remarkable difference in the phosphorescence profiles between the frozen MeTHF solution (77 K) and crystalline powder (295 K, Figures 2A–H) demonstrates the importance of the strong intermolecular contacts in the crystal lattice to realize twisted and tilted geometry of the carbazole moiety and enable efficient pRTP luminescence at room temperature. We explain such a change in the phosphorescence profiles for the 77 K frozen MeTHF glass and 295 K pRTP microcrystalline solid samples with the difference in the protocol of the solid sample preparation, thus resulting in a different environment of the pRTP molecule in the solid state. The microcrystalline samples have only one conformer with a particular set of intermolecular contacts between the RTP molecules. However, the 77 K frozen glass samples are obtained by freezing diluted 10^−4^ M MeTHF solution, therefore preventing the formation of intermolecular contacts between RTP molecules which are likely surrounded by multiple MeTHF molecules. The MeTHF spectra at 77 K are nearly identical for the materials obtained from the laboratory-synthesized and commercial carbazole samples (Figure 2, blue profiles). This indicates the importance of the particular intermolecular interactions and conformation of the RTP material, which can only be realized in the solid state (see Supplementary Materials).

### Computational results

**FIGURE 4:**
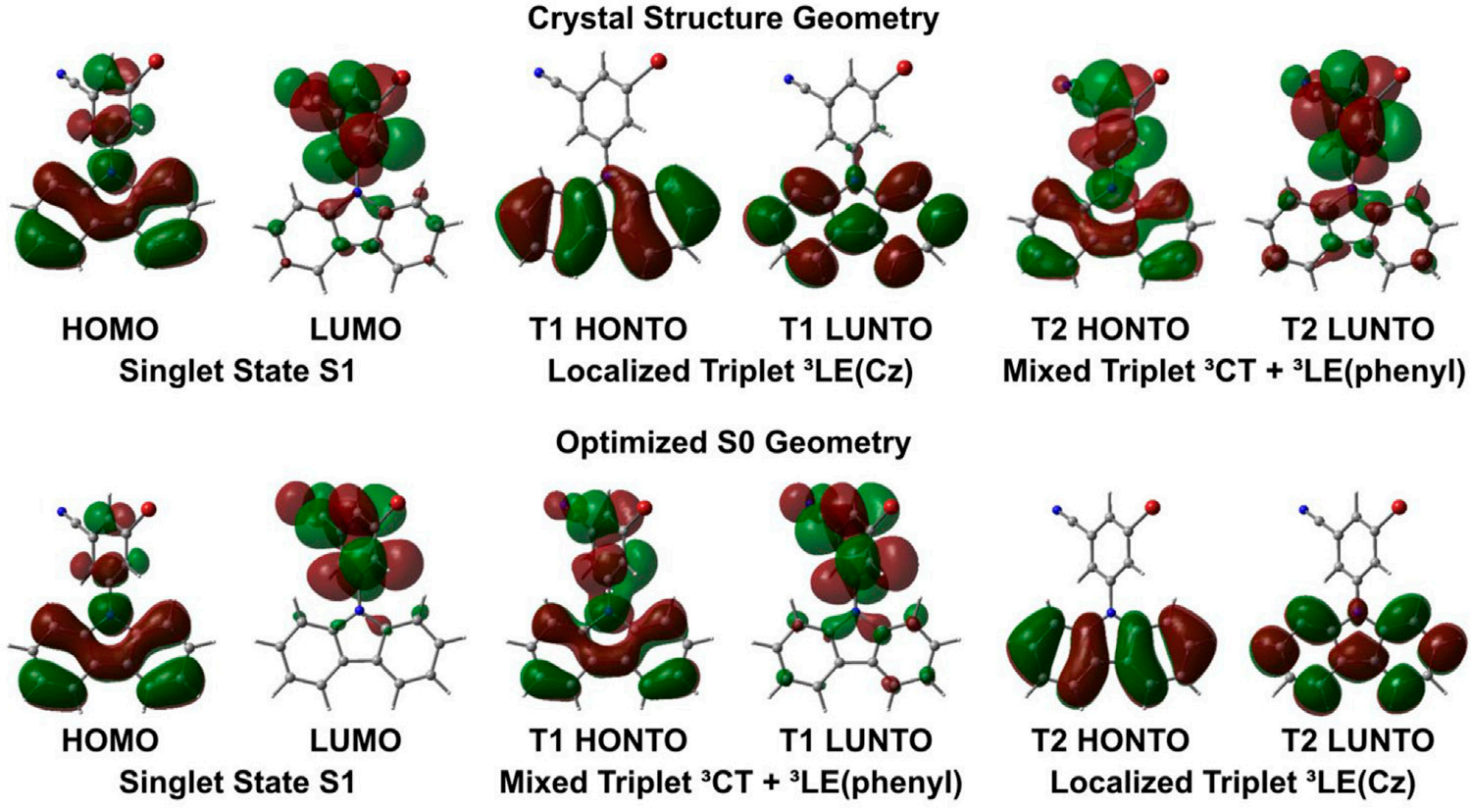
Calculated HOMO, LUMO, HONTO, and LUNTO plots of **RTP-Br** calculated from crystal geometries and optimized S0 geometries.

We investigated the electronic structure of the pRTP materials using density functional theory (DFT) for the ground state and time-dependent DFT (TD-DFT) with Tamm–Dancoff approximation (Hirata and Head-Gordon, 1999; Furche and Rappoport, 2005) for the excited states calculations, using the MN15 functional by Yu et al. (2016) in combination with def2-TZVP basis set by Ahlrichs. Andrae et al. (1990); Peterson et al. (2003); Weigend et al. (1998), Freeman et al. (2005), and Weigend and Ahlrichs (2005). TD-DFT calculations were performed to elucidate the nature of the excited state in a crystalline molecular geometry of the materials with all data collected (Supplementary Tables S2–S5). All materials have a high-transition dipole moment in the range of 10–15 D (see Supplementary Table S3), which is largely oriented along the C4-N2 bond between phenyl and carbazole moieties. All meta-substituted compounds **RTP-Halide** (halide = Cl, Br, I) have the highest occupied molecular orbital (HOMO) delocalized over the N-phenyl-carbazole unit of the **RTP-Halide** molecule, whereas ortho-substituted compounds **RTP-o-CN** and **RTP-o-Br** show localization of the HOMO orbital predominantly on the carbazole moiety (Figure 4, Supplementary Table S2). The lowest unoccupied molecular orbital (LUMO) is largely localized on the phenyl moiety for all compounds (Figure 4). Theoretical calculation predicts vertical excitation to the first excited singlet state S1 having a charge transfer nature (^1^CT), having 91%–98% HOMO−LUMO character for pRTP materials for all compounds. Theoretical calculations allowed us to assign the nature of the triplet states observed in pRTP materials based on the highest occupied and lowest unoccupied natural-transition orbitals (HONTO and LUNTO) for T1 and T2 excited states (Figure 3, Supplementary Tables S4, S5). (Martin, 2003). The first type of the triplet state has a charge transfer character ^3^CT mixed with local triplet ^3^LE (Cz) on the phenyl moiety, and the second type is a ^3^LE (Cz) state which is localized on carbazole (Figure 4). Theoretical calculations for the RTP-Br in the constrained crystal structure geometry indicate that the lowest in energy-excited triplet state T1 has a ^3^LE character resulting in efficient pRTP properties. However, the T1 triplet excited state is calculated to have ^3^CT-character when the structure of RTP-Br is optimized in a relaxed S_0_ geometry where we see less twists and tilts of the carbazole unit. This fact further demonstrates that the twisting and tilting of the carbazole enabled by strong intermolecular contacts in the crystal lattice is essential to modulate the relative energy of the ^3^CT and ^3^LE states (Figure 4). Theoretical calculations predict the energy gap between singlet (^1^CT) and triplet (^3^LE) excited states to be in the range of 0.3–0.5 eV (Supplementary Table S4), thus explaining a relatively poor delayed luminescence efficiency for the materials in the constrained crystalline geometry.

**FIGURE 5:**
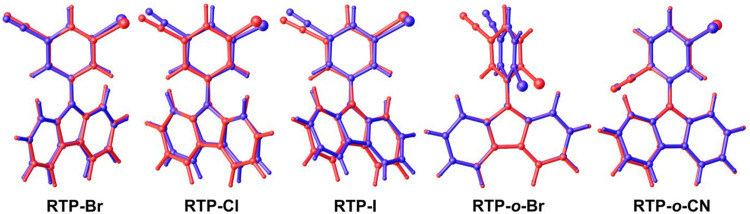
Superposition of X-ray crystal structure (blue) and theoretically calculated structure in S_0_ geometry (red).

The highest oscillator strength coefficients are calculated for **RTP-o-CN** (0.0569) and **RTP-Br** (0.1109), while the lowest oscillator strength coefficients are calculated for the **RTP-Cl**, **RTP-I**, and **RTP-o-Br**. High oscillator strength coefficients correlate well with the high experimental PLQY values over 20% measured for **RTP-o-CN** and **RTP-Br**. We optimized the geometry of pRTP materials in the ground (S_0_) state and superimposed it with the crystal structure’s geometry to explain potential non-radiative processes resulting in poor PLQY values for **RTP-Cl**, **RTP-I,** and **RTP-o-Br**. We found a significant twist-distortion between phenyl and carbazole moieties (Figure 4) for the **RTP-Cl**, **RTP-I,** and **RTP-o-Br** (correlates well with poor PLQY and low oscillator strength coefficients), whereas **RTP-o-CN** or **RTP-Br** show a good fit between overlaid structures with only a minor twist between phenyl and carbazole moieties (see Figure 4) supporting high PLQY values. Therefore, our theoretical result supports the experiment observation for **RTP-o-CN** and **RTP-Br** materials having a significantly stronger intermolecular interaction network (see Supplementary Materials) that suppresses the non-radiative processes in the crystalline state, which is likely associated with a twist distortion shown in Figure 5.

## Conclusion

We demonstrated a series of the halobenzonitrile–carbazole compounds with bright pRTP luminescence (PLQY up to 21% and excited state lifetime up to 0.4 s), which originate due to an impurity of 1H-benzo[f]indole (Bz) in commercially available carbazole. All materials obtained from the laboratory-synthesized carbazole demonstrate weak delayed luminescence and no pRTP luminescence. The position of the halogen and cyano-group on the 9-phenyl carbazole core severely affected the pRTP properties of the halobenzonitrile–carbazole materials by altering electronic and geometric parameters. The best performing **RTP-Br** and **RTP-*o*-CN** materials exhibit minor twist and tilt of the carbazole with respect to the phenyl moiety while having a strong bifurcated intermolecular hydrogen bond in the crystal lattice enabling high PLQY values over 20%. A weak 3D network of hydrogen bonds in **RTP-Cl** and **RTP-I** (C−H···halide and C−H···π) is unable to prevent the non-radiative distortions of the phenyl moiety with respect to carbazole ligand. Theoretical calculations supported the finding that the geometric distortions in the crystal lattice are likely the major reason for lower pRTP PLQY values. Therefore, future works will benefit if they consider the effect of the intermolecular contact network and conformation of carbazole ligand (twisting or tilting) guiding toward new bright pRTP materials enabled with an even longer excited state lifetime.

## Experimental part

### General methods

All reactions were performed under a N_2_ atmosphere. Solvents were dried as required. Sodium hydride was washed from mineral oil with diethyl ether and dried prior to use. Carbazole and fluorinated halobenzonitriles were purchased from Fluorochem and Tokyo Chemical Industry (TCI). A laboratory-synthesized sample of the carbazole was obtained according to the reported literature procedure (Freeman et al., 2005). ^1^H and ^13^C{^1^H} NMR spectra were recorded using a Bruker AVIII HD 500 MHz NMR spectrometer. ^1^H NMR spectra (500.19 MHz) and ^13^C{^1^H} (125.79 MHz) were referenced to acetone-d_6_ at δ 2.05 (^13^C, δ 29.84). Elemental analyses were performed by the Microanalysis Laboratory at the University of Manchester. Mass spectrometry data were obtained by the Mass Spectrometry Laboratory at the University of Manchester.

Photoluminescence measurements were recorded on an Edinburgh Instruments FLS980 spectrometer with a solid mount attachment where appropriate. Absolute photoluminescence quantum yields were recorded using Hamamatsu Quantaurus-QY C11347-11. Quantum yields have been measured in air for solid samples. Time-resolved luminescence data were collected on a time-correlated single-photon counting (TCSPC) Edinburgh Instruments FLS980 spectrometer using F-900 software. A xenon flash lamp was used as the excitation source. The collected data were analyzed using F-900 software.

### X-ray crystallography

Crystals were mounted in oil on glass fiber and fixed on the diffractometer in a cold nitrogen stream. Data were collected using an Agilent SuperNova with Mo Kα (*λ* = 0.71073 Å) radiation at 100 K. Data were processed using the CrystAlisPro-CCD and–RED software (Programs, 2010). The structure was solved by intrinsic phasing or direct method and refined by the full-matrix least-squares against F2 in an anisotropic (for non-hydrogen atoms) approximation. All hydrogen atom positions were refined in isotropic approximation in a “riding” model with the U_iso_(H) parameters equal to 1.2 U_eq_ (C_*i*_) for methyl groups equal to 1.5 U_eq_ (C_*ii*_), where U(Ci) and U(Cii) are, respectively, the equivalent thermal parameters of the carbon atoms to which the corresponding H atoms are bonded. All calculations were performed using SHELXTL software (Sheldrick, 2015a; Sheldrick, 2015b). OLEX2 software was used as a graphical user interface (Dolomanov et al., 2009).

### Crystal data for RTP-Cl: CCDC 2195535

C_19_H_11_ClN_2_ (*M* =302.75 g/mol): monoclinic, space group P2_1_/c (no. 14), *a* = 15.974(2) Å, *b* = 4.8752(7) Å, *c* = 18.790(3) Å, *β* = 94.909(12)°, *V* = 1,457.9(3) Å^3^, *Z* = 4, *T* = 100.01(10) K, μ(Mo Kα) = 0.259 mm^−1^, *Dcalc* = 1.379 g/cm^3^, 10,371 reflections measured (6.998° ≤ 2Θ ≤ 58.456°), and 3,469 were unique (*R*_int_ = 0.1121, R_sigma_ = 0.1467), which were used in all calculations. The final *R*_1_ was 0.0938 (I > 2σ(I)), and *wR*_2_ was 0.2853 (all data).

### Crystal data for RTP-Br: CCDC 2195533

C_19_H_11_BrN_2_ (*M* =347.21 g/mol): triclinic, space group P-1 (no. 2), *a* = 3.9322(2) Å, *b* = 10.0657(5) Å, *c* = 18.0728(8) Å, *α* = 88.000(4)°, *β* = 84.762(3)°, *γ* = 86.165(4)°, *V* = 710.45(6) Å^3^, *Z* = 2, *T* = 100.01(10) K, μ(Mo Kα) = 2.890 mm^−1^, *Dcalc* = 1.623 g/cm^3^, 9,735 reflections measured (6.794° ≤ 2Θ ≤ 57.968°), and 3,344 were unique (*R*_int_ = 0.0914, R_sigma_ = 0.0981), which were used in all calculations. The final *R*_1_ was 0.0516 (I > 2σ(I)), and *wR*_2_ was 0.1202 (all data).

### Crystal data for RTP-I: CCDC 2195534

C_19_H_11_IN_2_ (*M* =394.20 g/mol): monoclinic, space group P2_1_/c (no. 14), *a* = 17.5810(9) Å, *b* = 5.0921(2) Å, *c* = 18.9085 (12) Å, *β* = 116.944(7)°, *V* = 1,509.02(16) Å^3^, *Z* = 4, *T* = 100.00(10) K, μ(Mo Kα) = 2.119 mm^−1^, *Dcalc* = 1.735 g/cm^3^, 11,305 reflections measured (7.074° ≤ 2Θ ≤ 58.22°), and 3,603 unique (*R*_int_ = 0.0548, R_sigma_ = 0.0669), which were used in all calculations. The final *R*_1_ was 0.0375 (I > 2σ(I)), and *wR*_2_ was 0.0786 (all data).

### Crystal data for RTP-o-CN: CCDC 2195537

C_19_H_11_BrN_2_ (*M* =347.21 g/mol): monoclinic, space group P2_1_/c (no. 14), *a* = 9.2158(5) Å, *b* = 14.4330(6) Å, *c* = 11.7189(6) Å, *β* = 111.130(6)°, *V* = 1,453.95 (14) Å^3^, *Z* = 4, *T* = 100.00 (10) K, μ(Mo Kα) = 2.824 mm^−1^, *Dcalc* = 1.586 g/cm^3^, 10,955 reflections measured (6.766° ≤ 2Θ ≤ 58.18°), and 3,465 were unique (*R*_int_ = 0.0444, R_sigma_ = 0.0512), which were used in all calculations. The final *R*_1_ was 0.0355 (I > 2σ(I)), and *wR*_2_ was 0.0819 (all data).

### Crystal data for RTP-o-Br: CCDC 2195536

C_38_H_22_Br_2_N_4_ (*M* =694.41 g/mol): monoclinic, space group P2_1_/c (no. 14), *a* = 15.9502(7) Å, *b* = 12.5562(4) Å, *c* = 15.9434(8) Å, *β* = 113.440(5)°, *V* = 2,929.5(2) Å^3^, *Z* = 4, *T* = 100.01 (10) K, μ(Mo Kα) = 2.803 mm^−1^, *Dcalc* = 1.574 g/cm^3^, 10,956 reflections measured (6.92° ≤ 2Θ ≤ 57.846°), and 6,221 were unique (*R*_int_ = 0.0297, R_sigma_ = 0.0563), which were used in all calculations. The final *R*_1_ was 0.0415 (I > 2σ(I)), and *wR*_2_ was 0.0997 (all data).

### Synthesis of RTP-Cl

Carbazole (1.00 g, 5.98 mmol) was added to a suspension of NaH (143 mg, 5.98 mmol) in DMF (20 ml) at 0°C under a stream of N_2_. The reaction mixture was stirred for 1 h at room temperature. 3-Chloro-5-fluorobenzonitrile (1.02 g, 6.56 mmol) was added to the reaction mixture under N_2_ and was heated to 120°C and left to stir overnight. The reaction mixture was cooled to room temperature and poured into water. The product was extracted with DCM and dried with MgSO_4_. Recrystallization by dissolving the product in a minimum amount of DCM, layering with EtOH, and cooling gave the pure product as a white crystalline powder with 50% yield (912 mg, 3.01 mmol). Single crystals suitable for X-ray diffraction were grown by layering a concentrated solution in DCM with EtOH and cooling. ^1^H NMR (500 MHz, acetone) δ 8.23 (d, *J* = 7.8 Hz, 2H, Hg), 8.12 (t, *J* = 1.7 Hz, 1H, Hc), 8.09 (t, *J* = 2.0 Hz, 1H, Hb), 8.06–8.03 (m, 1H, Ha), 7.53 (d, *J* = 8.2 Hz, 2H, Hd), 7.47 (ddd, *J* = 8.2, 7.0, 1.2 Hz, 2H, He), and 7.34 (td, *J* = 7.5, 1.1 Hz, 2H, Hf). ^13^C NMR (126 MHz, acetone) δ 141.13 (Cz C-N), 140.85 (Ph C-N), 137.03 (Ph C-Cl), 132.71 (Ph Cb), 131.62 (Ph Cc), 130.15 (Ph Ca), 127.38 (Cz Ce), 124.64 (Cz C-C), 121.83 (Cz Cf), 121.30 (Cz Cg), 117.52 (Ph C-CN), 116.42 (CN), and 110.53 (Cz Cd). Anal. Calcd. For C_19_H_11_ClN_2_: C, 75.38; H, 3.66; N, 9.25. Found: C, 74.98; H, 3.56; N, 9.30. HRMS C_19_H_11_ClN_2_ theoretical [M + H]^+^ = 303.0684, HRMS (APCI): = 303.0675.

### Synthesis of RTP-Br

The compound was prepared by following the literature procedure (Ihn et al., 2017). Single crystals suitable for X-ray diffraction were grown by layering a concentrated solution in DCM with EtOH and cooling. HRMS C_19_H_11_BrN_2_ theoretical [M + H]^+^ = 347.0178, HRMS (APCI): = 347.0178. Anal. Calcd. For C_19_H_11_BrN_2_: C, 65.73; H, 3.19; N, 8.07. Found: C, 63.22; H, 3.01; N, 7.81.

### Synthesis of RTP-I

Carbazole (307 mg, 1.84 mmol) was added to a suspension of NaH (44.1 mg, 1.84 mmol) in DMF (20 ml) at 0°C under a stream of N_2_. The reaction mixture was stirred for 1 h at room temperature. 3-Chloro-5-fluorobenzonitrile (1.02 g, 6.56 mmol) was added to the reaction mixture under N_2_, and was heated to 120°C, and left to stir overnight. The reaction mixture was cooled to room temperature and poured into water. The product was extracted with DCM and dried with MgSO_4_. Recrystallization by dissolving the product in a minimum amount of DCM and layering with EtOH gave the pure product as an off-white crystalline powder with 51% yield (372 mg, 945 μmol). Single crystals suitable for X-ray diffraction were grown by layering a concentrated solution in DCM with EtOH and cooling. ^1^H NMR (500 MHz, acetone) δ 8.36 (t, *J* = 1.8 Hz, 1H, Hc), 8.31 (t, *J* = 1.4 Hz, 1H, Hb), 8.21 (d, *J* = 7.8 Hz, 2H, Hg), 8.15 (t, *J* = 1.8 Hz, 1H, Ha), 7.52–7.41 (m, 4H, Hd/e), 7.33 (td, *J* = 7.4, 1.4 Hz, 2H, Hf). ^13^C NMR (126 MHz, acetone) δ 141.31 (Cz C-N), 141.14 (Ph C-N), 140.34 (Ph Cc), 140.16 (Ph Cb), 130.91 (Ph Ca), 127.35 (Cz Ce), 124.59 (Cz C-C), 121.76 (Cz Cf), 121.29 (Cz Cg), 117.15 (Ph C-CN), 116.46 (CN), 110.46 (Cz Cd), 95.47 (Ph C-I). Anal. Calcd. For C_19_H_11_IN_2_: C, 57.89; H, 2.81; N, 7.11. Found: C, 57.86; H, 2.73; N, 7.23. HRMS C_19_H_11_BrN_2_ theoretical [M + H]^+^ = 395.0040, HRMS (APCI): = 395.0014.

### Synthesis of RTP-*o*-CN

Carbazole (1.00 g, 5.98 mmol) was added to a suspension of NaH (143 mg, 5.98 mmol) in DMF (20 ml) at 0°C under a stream of N_2_. The reaction mixture was stirred for 1 h at room temperature. 4-Bromo-2-fluorobenzonitrile (1.32 g, 6.60 mmol) was added to the reaction mixture under N_2_, and was heated to 120°C, and left to stir overnight. The reaction mixture was cooled to room temperature and poured into water. The product was extracted with DCM and dried with MgSO_4_. Recrystallization by cooling an EtOH solution gave the pure product as a white crystalline powder with 85% yield (1.76 g, 5.08 mmol). Single crystals suitable for X-ray diffraction were grown by layering a concentrated solution in DCM with EtOH and cooling. ^1^H NMR (500 MHz, acetone) δ 8.24 (d, *J* = 7.8 Hz, 2H, Hg), 8.09 (d, *J* = 8.2 Hz, 1H, Hb), 8.07–8.00 (m, 2H, Ha/c), 7.47 (ddd, *J* = 8.4, 7.2, 1.2 Hz, 2H, Hd), 7.35 (td, *J* = 7.6, 1.1 Hz, 2H, He), 7.31 (d, *J* = 8.2 Hz, 2H, Hf). ^13^C NMR (126 MHz, acetone) δ 142.14 (Cz C-N), 141.65 (Ph C-N), 136.79 (Ph Cb), 134.03 (Ph Ca), 133.48 (Ph Cc), 129.28 (Ph C-Br), 127.33 (Cz Cd), 124.64 (Cz C-C), 121.85 (Cz Ce), 121.38 (Cz Cg), 116.19 (CN), 112.87 (Ph C-CN), 110.72 (Cz Cf). Anal. Calcd. For C_19_H_11_BrN_2_: C, 65.73; H, 3.19; N, 8.07. Found: C, 65.53; H, 3.10; N, 8.09. HRMS C_19_H_11_BrN_2_ theoretical [M + H]^+^ = 347.0178, HRMS (APCI): = 347.0179.

### Synthesis of RTP-*o*-Br

Carbazole (1.00 g, 5.98 mmol) was added to a suspension of NaH (143 mg, 5.98 mmol) in DMF (20 ml) at 0°C under a stream of N_2_. The reaction mixture was stirred for 1 h at room temperature. 4-Bromo-3-fluorobenzonitrile (1.32 g, 6.60 mmol) was added to the reaction mixture under N_2_, and was heated to 120°C, and left to stir overnight. The reaction mixture was cooled to room temperature and poured into water. The product was extracted with DCM and dried with MgSO_4_. Recrystallization by cooling an EtOH solution gave the pure product as a white crystalline powder with 65% yield (1.35 g, 5.98 mmol). Single crystals suitable for X-ray diffraction were grown by layering a concentrated solution in DCM with EtOH and cooling. ^1^H NMR (500 MHz, acetone) δ 8.23 (pseudo dd, *J* = 8.1, 6.0 Hz, 3H, Hg/c), 8.15 (d, *J* = 2.0 Hz, 1H, Hb), 7.99 (dd, *J* = 8.4, 2.0 Hz, 1H, Ha), 7.43 (ddd, *J* = 8.2, 7.2, 1.2 Hz, 2H, Hd), 7.35–7.28 (m, 2H, He), 7.12 (d, *J* = 8.1 Hz, 2H, Hf). ^13^C NMR (126 MHz, acetone) δ 141.49 (Cz C-N), 138.69 (Ph C-N), 136.60 (Ph Cc), 135.95 (Ph Cb), 134.77 (Ph Ca), 130.34 (Ph C-Br), 127.16 (Cz Cd), 124.29 (Cz C-C), 121.35 (Cz Ce), 121.28 (Cz Cg), 117.91 (CN), 114.30 (Ph C-CN), 110.74 (Cz Cf). Anal. Calcd. For C_19_H_11_BrN_2_: C, 65.73; H, 3.19; N, 8.07. Found: C, 65.91; H, 3.08; N, 8.25. HRMS C_19_H_11_BrN_2_ theoretical [M + H]^+^ = 347.0178, HRMS (APCI): = 347.0179.

## Data Availability

The original contributions presented in the study are included in the article/Supplementary Materials; further inquiries can be directed to the corresponding authors.
