# Supplementary material for: Organic persistent room temperature phosphorescence enabled by carbazole impurity
Source: Front Chem. 2023 Jan 6;10:1008658. doi: 10.3389/fchem.2022.1008658 (PMC9853050; doi:10.3389/fchem.2022.1008658)

checkCIF/PLATON report

Structure factors have been supplied for datablock(s) RTPBr, RTPCl, RTPI, RTPoBr, RTPoCN

THIS REPORT IS FOR GUIDANCE ONLY. IF USED AS PART OF A REVIEW PROCEDURE FOR PUBLICATION, IT SHOULD NOT REPLACE THE EXPERTISE OF AN EXPERIENCED CRYSTALLOGRAPHIC REFEREE.

No syntax errors found. CIF dictionary Interpreting this report

Datablock: RTPCl

```
Bond precision:   C-C = 0.0062 A          Wavelength=0.71073

Cell:              a=15.974(2)     b=4.8752(7)          c=18.790(3)
                   alpha=90        beta=94.909(12)      gamma=90
Temperature:       100 K

                 Calculated                       Reported
Volume           1457.9(4)                        1457.9(3)
Space group      P 21/c                           P 1 21/c 1
Hall group       -P 2ybc                          -P 2ybc
Moiety formula   C19 H11 Cl N2                    C19 H11 Cl N2
Sum formula      C19 H11 Cl N2                    C19 H11 Cl N2
Mr               302.75                           302.75
Dx,g cm-3        1.379                            1.379
Z                4                                4
Mu (mm-1)        0.259                            0.259
F000             624.0                            624.0
F000'            624.79
h,k,lmax         19,6,23                          19,6,23
Nref             2845                             2839
Tmin,Tmax        0.959,0.985                      0.397,1.000
Tmin'            0.827

Correction method= # Reported T Limits: Tmin=0.397 Tmax=1.000
AbsCorr = MULTI-SCAN

Data completeness= 0.998           Theta(max)= 25.994

R(reflections)= 0.0896( 1645)                          wR2(reflections)=
                                                       0.2593( 2839)
S = 1.044                    Npar= 193
```

The following ALERTS were generated. Each ALERT has the format
test-name_ALERT_alert-type_alert-level.
Click on the hyperlinks for more details of the test.

Alert level C

```
PLAT084_ALERT_3_C High wR2 Value (i.e. > 0.25) ...................       0.26 Report
PLAT340_ALERT_3_C Low Bond Precision on  C-C Bonds ...............     0.0062 Ang.
PLAT906_ALERT_3_C Large K Value in the Analysis of Variance ......      4.073 Check
PLAT910_ALERT_3_C Missing # of FCF Reflection(s) Below Theta(Min).          6 Note
```

Alert level G

```
PLAT063_ALERT_4_G Crystal Size Possibly too Large for Beam Size ..       0.73 mm
PLAT072_ALERT_2_G SHELXL First  Parameter in WGHT  Unusually Large       0.10 Report
PLAT171_ALERT_4_G The CIF-Embedded .res File Contains EADP Records          1 Report
PLAT230_ALERT_2_G Hirshfeld Test Diff for    N1       --C7       .        9.2 s.u.
PLAT941_ALERT_3_G Average HKL Measurement Multiplicity ...........        3.3 Low
PLAT955_ALERT_1_G Reported (CIF) and Actual (FCF) Lmax Differ by .          1 Units
PLAT967_ALERT_5_G Note: Two-Theta Cutoff Value in Embedded .res ..       52.0 Degree
PLAT978_ALERT_2_G Number C-C Bonds with Positive Residual Density.          0 Info
```

0 **ALERT level A** = Most likely a serious problem - resolve or explain
0 **ALERT level B** = A potentially serious problem, consider carefully
4 **ALERT level C** = Check. Ensure it is not caused by an omission or oversight
8 **ALERT level G** = General information/check it is not something unexpected

1 ALERT type 1 CIF construction/syntax error, inconsistent or missing data
3 ALERT type 2 Indicator that the structure model may be wrong or deficient
5 ALERT type 3 Indicator that the structure quality may be low
2 ALERT type 4 Improvement, methodology, query or suggestion
1 ALERT type 5 Informative message, check

Datablock: RTPBr

Bond precision: C-C = 0.0054 A Wavelength=0.71073

Cell:	a=3.9322(2)	b=10.0657(5)	c=18.0728(8)
	alpha=88.000(4)	beta=84.762(3)	gamma=86.165(4)
Temperature:	100 K		

	Calculated	Reported
Volume	710.45(6)	710.45(6)
Space group	P -1	P -1
Hall group	-P 1	-P 1
Moiety formula	C19 H11 Br N2	C19 H11 Br N2
Sum formula	C19 H11 Br N2	C19 H11 Br N2
Mr	347.20	347.21
Dx,g cm-3	1.623	1.623
Z	2	2
Mu (mm-1)	2.890	2.890
F000	348.0	348.0
F000'	347.54	
h,k,lmax	5,13,24	5,13,24
Nref	3790	3344
Tmin,Tmax	0.530,0.838	0.493,1.000
Tmin'	0.459	

Correction method= # Reported T Limits: Tmin=0.493 Tmax=1.000
AbsCorr = MULTI-SCAN

Data completeness= 0.882 Theta(max)= 28.984

R(reflections)= 0.0516(2647) wR2(reflections)= 0.1202(3344)

S = 1.061 Npar= 199

The following ALERTS were generated. Each ALERT has the format
test-name_ALERT_alert-type_alert-level.
Click on the hyperlinks for more details of the test.

Alert level C

PLAT910_ALERT_3_C Missing # of FCF Reflection(s) Below Theta(Min). 7 Note

---

Alert level G

PLAT230_ALERT_2_G Hirshfeld Test Diff for C6 --C7 . 7.7 s.u.
PLAT912_ALERT_4_G Missing # of FCF Reflections Above STh/L= 0.600 440 Note
PLAT941_ALERT_3_G Average HKL Measurement Multiplicity .... 2.9 Low
PLAT978_ALERT_2_G Number C-C Bonds with Positive Residual Density. 4 Info

---

0 **ALERT level A** = Most likely a serious problem - resolve or explain
0 **ALERT level B** = A potentially serious problem, consider carefully
1 **ALERT level C** = Check. Ensure it is not caused by an omission or oversight
4 **ALERT level G** = General information/check it is not something unexpected

0 ALERT type 1 CIF construction/syntax error, inconsistent or missing data

```
   2 ALERT type 2 Indicator that the structure model may be wrong or deficient
   2 ALERT type 3 Indicator that the structure quality may be low
   1 ALERT type 4 Improvement, methodology, query or suggestion
   0 ALERT type 5 Informative message, check
```

Datablock: RTPI

```
Bond precision:   C-C = 0.0047 A          Wavelength=0.71073

Cell:             a=17.5810(9)     b=5.0921(2)         c=18.9085(12)
                  alpha=90         beta=116.944(7)     gamma=90
Temperature:      100 K

                 Calculated                    Reported
Volume           1509.02(17)                   1509.02(16)
Space group      P 21/c                        P 1 21/c 1
Hall group       -P 2ybc                       -P 2ybc
Moiety formula   C19 H11 I N2                  C19 H11 I N2
Sum formula      C19 H11 I N2                  C19 H11 I N2
Mr               394.20                        394.20
Dx,g cm-3        1.735                         1.735
Z                4                             4
Mu (mm-1)        2.119                         2.119
F000             768.0                         768.0
F000'            766.33
h,k,lmax         24,6,25                       22,6,25
Nref             4038                          3603
Tmin,Tmax        0.504,0.879                   0.030,1.000
Tmin'            0.368

Correction method= # Reported T Limits: Tmin=0.030 Tmax=1.000
AbsCorr = MULTI-SCAN

Data completeness= 0.892           Theta(max)= 29.110

                                                         wR2(reflections)=
R(reflections)= 0.0375( 2988)                            0.0786( 3603)

S = 1.052                  Npar= 199
```

The following ALERTS were generated. Each ALERT has the format
test-name_ALERT_alert-type_alert-level.
Click on the hyperlinks for more details of the test.

Alert level C

```
PLAT910_ALERT_3_C Missing # of FCF Reflection(s) Below Theta(Min).          6 Note
```

Alert level G

```
PLAT720_ALERT_4_G Number of Unusual/Non-Standard Labels ..........          1 Note
PLAT912_ALERT_4_G Missing # of FCF Reflections Above STh/L=  0.600        406 Note
PLAT941_ALERT_3_G Average HKL Measurement Multiplicity ...........        3.1 Low
PLAT950_ALERT_5_G Calculated (ThMax) and CIF-Reported Hmax Differ           2 Units
PLAT956_ALERT_1_G Calculated (ThMax) and Actual (FCF) Hmax Differ           2 Units
PLAT978_ALERT_2_G Number C-C Bonds with Positive Residual Density.          4 Info
```

```
   0 ALERT level A = Most likely a serious problem - resolve or explain
   0 ALERT level B = A potentially serious problem, consider carefully
   1 ALERT level C = Check. Ensure it is not caused by an omission or oversight
   6 ALERT level G = General information/check it is not something unexpected

   1 ALERT type 1 CIF construction/syntax error, inconsistent or missing data
   1 ALERT type 2 Indicator that the structure model may be wrong or deficient
   2 ALERT type 3 Indicator that the structure quality may be low
   2 ALERT type 4 Improvement, methodology, query or suggestion
   1 ALERT type 5 Informative message, check
```

Datablock: RTPoCN

Bond precision:	C-C = 0.0033 A		Wavelength=0.71073
Cell:	a=9.2158(5)	b=14.4330(6)	c=11.7189(6)
	alpha=90	beta=111.130(6)	gamma=90
Temperature:	100 K		

	Calculated	Reported
Volume	1453.95(14)	1453.95(14)
Space group	P 21/c	P 1 21/c 1
Hall group	-P 2ybc	-P 2ybc
Moiety formula	C19 H11 Br N2	C19 H11 Br N2
Sum formula	C19 H11 Br N2	C19 H11 Br N2
Mr	347.20	347.21
Dx,g cm-3	1.586	1.586
Z	4	4
Mu (mm-1)	2.824	2.824
F000	696.0	696.0
F000'	695.08	
h,k,lmax	12,19,16	11,19,15
Nref	3911	3465
Tmin,Tmax	0.253,0.435	0.456,1.000
Tmin'	0.196	

Correction method= # Reported T Limits: Tmin=0.456 Tmax=1.000
AbsCorr = MULTI-SCAN

Data completeness= 0.886 Theta(max)= 29.090

R(reflections)= 0.0355(2872) wR2(reflections)= 0.0819(3465)

S = 1.034 Npar= 199

The following ALERTS were generated. Each ALERT has the format
test-name_ALERT_alert-type_alert-level.
Click on the hyperlinks for more details of the test.

Alert level C

PLAT910_ALERT_3_C Missing # of FCF Reflection(s) Below Theta(Min). 5 Note

---

Alert level G

PLAT912_ALERT_4_G Missing # of FCF Reflections Above STh/L= 0.600 393 Note
PLAT913_ALERT_3_G Missing # of Very Strong Reflections in FCF .... 1 Note
PLAT941_ALERT_3_G Average HKL Measurement Multiplicity .... 3.2 Low
PLAT978_ALERT_2_G Number C-C Bonds with Positive Residual Density. 9 Info

---

0 **ALERT level A** = Most likely a serious problem - resolve or explain
0 **ALERT level B** = A potentially serious problem, consider carefully
1 **ALERT level C** = Check. Ensure it is not caused by an omission or oversight
4 **ALERT level G** = General information/check it is not something unexpected

0 ALERT type 1 CIF construction/syntax error, inconsistent or missing data

1 ALERT type 2 Indicator that the structure model may be wrong or deficient
3 ALERT type 3 Indicator that the structure quality may be low
1 ALERT type 4 Improvement, methodology, query or suggestion
0 ALERT type 5 Informative message, check

Datablock: RTPoBr

Bond precision: C-C = 0.0043 A Wavelength=0.71073

Cell:	a=15.9502(7)	b=12.5562(4)	c=15.9434(8)
	alpha=90	beta=113.440(5)	gamma=90
Temperature:	100 K		

	Calculated	Reported
Volume	2929.6(2)	2929.5(2)
Space group	P 21/c	P 1 21/c 1
Hall group	-P 2ybc	-P 2ybc
Moiety formula	C19 H11 Br N2	2(C19 H11 Br N2)
Sum formula	C19 H11 Br N2	C38 H22 Br2 N4
Mr	347.20	694.41
Dx,g cm-3	1.574	1.574
Z	8	4
Mu (mm-1)	2.803	2.803
F000	1392.0	1392.0
F000’	1390.16	
h,k,lmax	21,17,21	21,16,20
Nref	7736	6221
Tmin,Tmax	0.509,0.639	0.504,1.000
Tmin’	0.356	

Correction method= # Reported T Limits: Tmin=0.504 Tmax=1.000
AbsCorr = MULTI-SCAN

Data completeness= 0.804 Theta(max)= 28.923

R(reflections)= 0.0415(4932) wR2(reflections)= 0.0997(6221)

S = 1.065 Npar= 397

The following ALERTS were generated. Each ALERT has the format
test-name_ALERT_alert-type_alert-level.
Click on the hyperlinks for more details of the test.

Alert level B

```
PLAT029_ALERT_3_B _diffrn_measured_fraction_theta_full value Low .      0.940 Why?
PLAT910_ALERT_3_B Missing # of FCF Reflection(s) Below Theta(Min).         14 Note
```

Alert level C

```
PLAT911_ALERT_3_C Missing FCF Refl Between Thmin & STh/L=    0.600        304 Report
PLAT934_ALERT_3_C Number of (Iobs-Icalc)/Sigma(W) > 10 Outliers ..          1 Check
```

Alert level G

```
PLAT045_ALERT_1_G Calculated and Reported Z Differ by a Factor ...          2 Check
PLAT431_ALERT_2_G Short Inter HL..A Contact  Br2      ..N1       .       3.17 Ang.
                                     -1+x,3/2-y,-1/2+z  =      4_475 Check
PLAT912_ALERT_4_G Missing # of FCF Reflections Above STh/L=  0.600       1159 Note
PLAT941_ALERT_3_G Average HKL Measurement Multiplicity ..........         1.8 Low
PLAT978_ALERT_2_G Number C-C Bonds with Positive Residual Density.          3 Info
```

---

0 **ALERT level A** = Most likely a serious problem - resolve or explain
2 **ALERT level B** = A potentially serious problem, consider carefully
2 **ALERT level C** = Check. Ensure it is not caused by an omission or oversight
5 **ALERT level G** = General information/check it is not something unexpected

1 ALERT type 1 CIF construction/syntax error, inconsistent or missing data
2 ALERT type 2 Indicator that the structure model may be wrong or deficient
5 ALERT type 3 Indicator that the structure quality may be low
1 ALERT type 4 Improvement, methodology, query or suggestion
0 ALERT type 5 Informative message, check

It is advisable to attempt to resolve as many as possible of the alerts in all categories. Often the minor alerts point to easily fixed oversights, errors and omissions in your CIF or refinement strategy, so attention to these fine details can be worthwhile. In order to resolve some of the more serious problems it may be necessary to carry out additional measurements or structure refinements. However, the purpose of your study may justify the reported deviations and the more serious of these should normally be commented upon in the discussion or experimental section of a paper or in the "special_details" fields of the CIF. checkCIF was carefully designed to identify outliers and unusual parameters, but every test has its limitations and alerts that are not important in a particular case may appear. Conversely, the absence of alerts does not guarantee there are no aspects of the results needing attention. It is up to the individual to critically assess their own results and, if necessary, seek expert advice.

Publication of your CIF in IUCr journals

A basic structural check has been run on your CIF. These basic checks will be run on all CIFs submitted for publication in IUCr journals (*Acta Crystallographica*, *Journal of Applied Crystallography*, *Journal of Synchrotron Radiation*); however, if you intend to submit to *Acta Crystallographica Section C* or *E* or *IUCrData*, you should make sure that full publication checks are run on the final version of your CIF prior to submission.

Publication of your CIF in other journals

Please refer to the *Notes for Authors* of the relevant journal for any special instructions relating to CIF submission.

PLATON version of 18/05/2022; check.def file version of 17/05/2022

Datablock RTPCl - ellipsoid plot

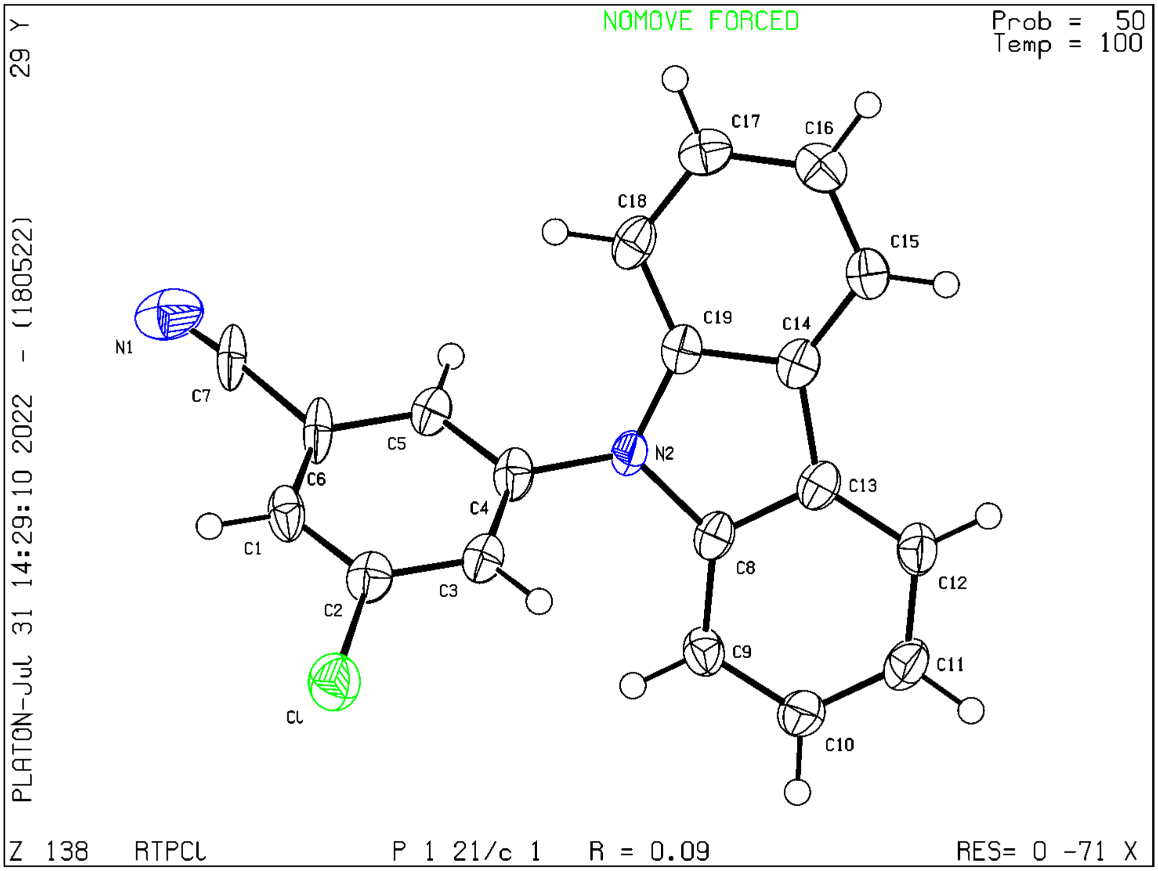

Datablock RTPBr - ellipsoid plot

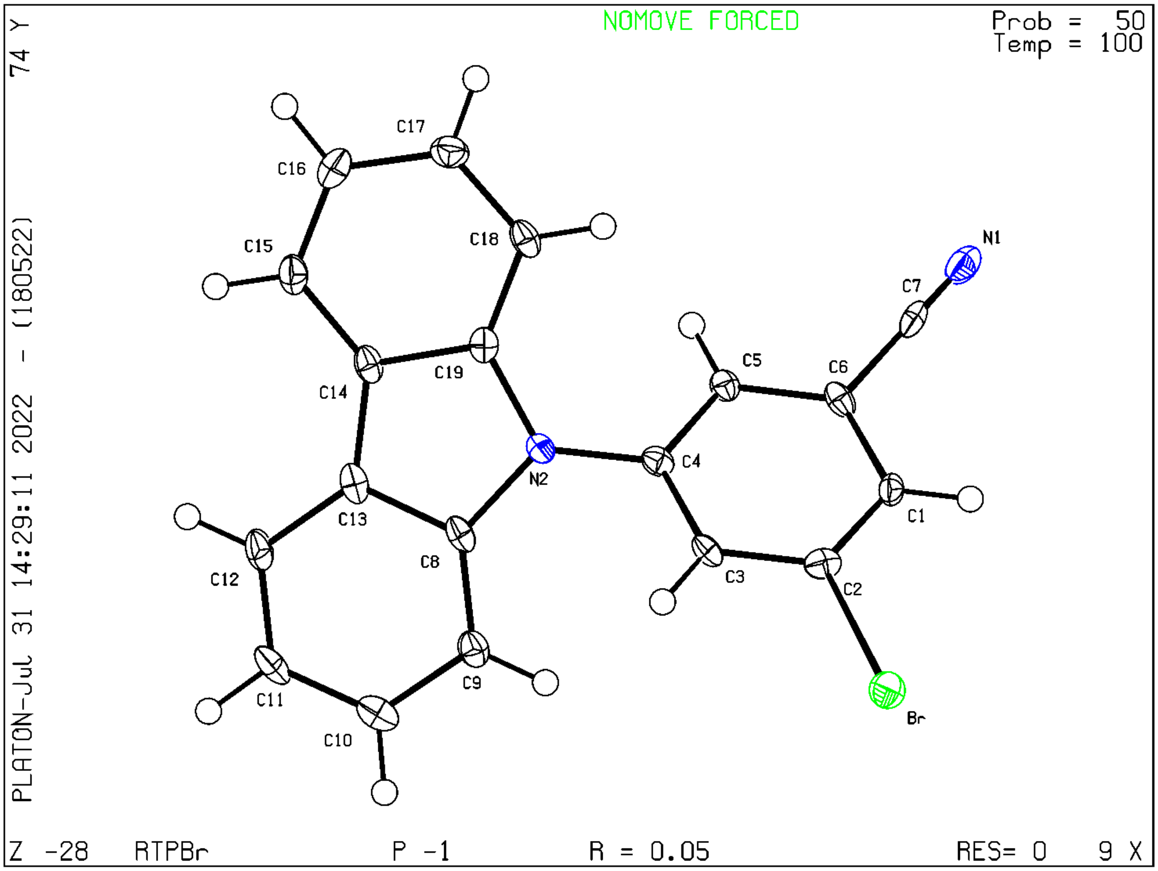

Datablock RTPI - ellipsoid plot

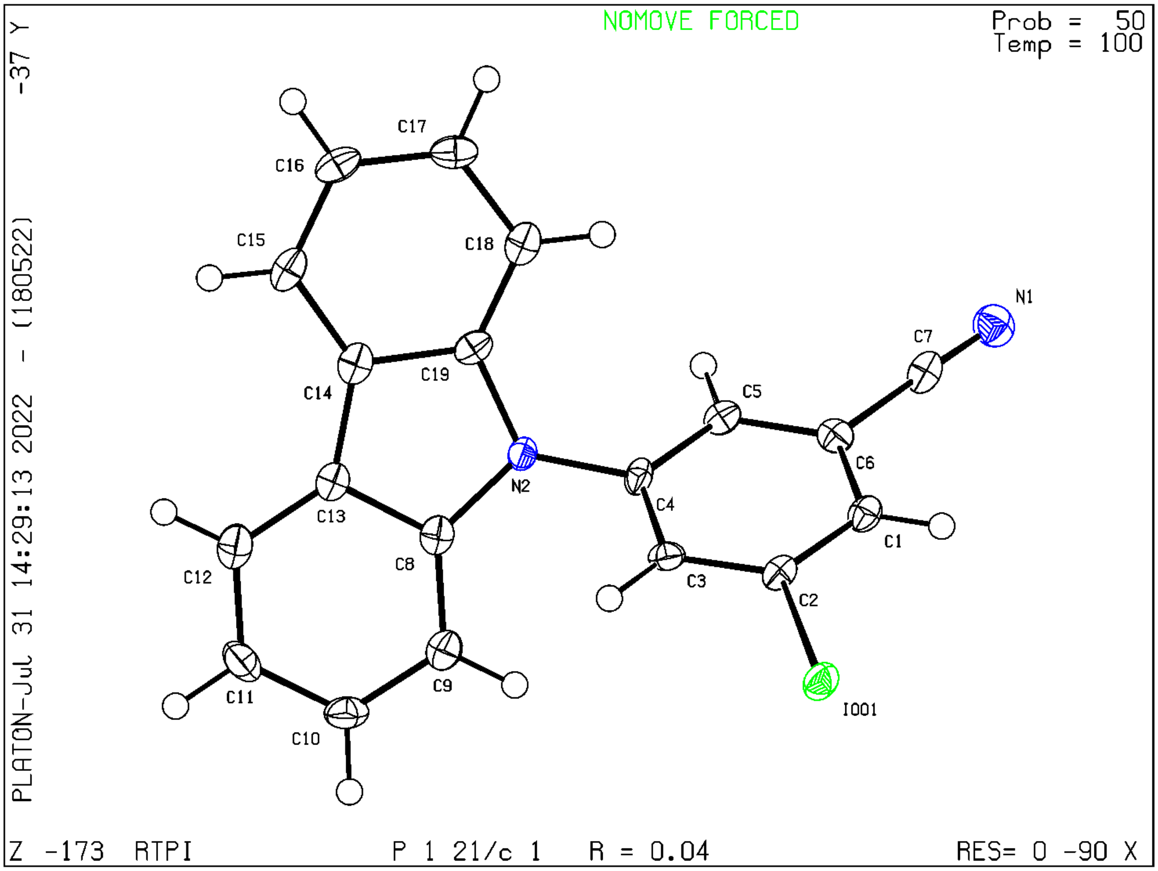

Datablock RTPoCN - ellipsoid plot

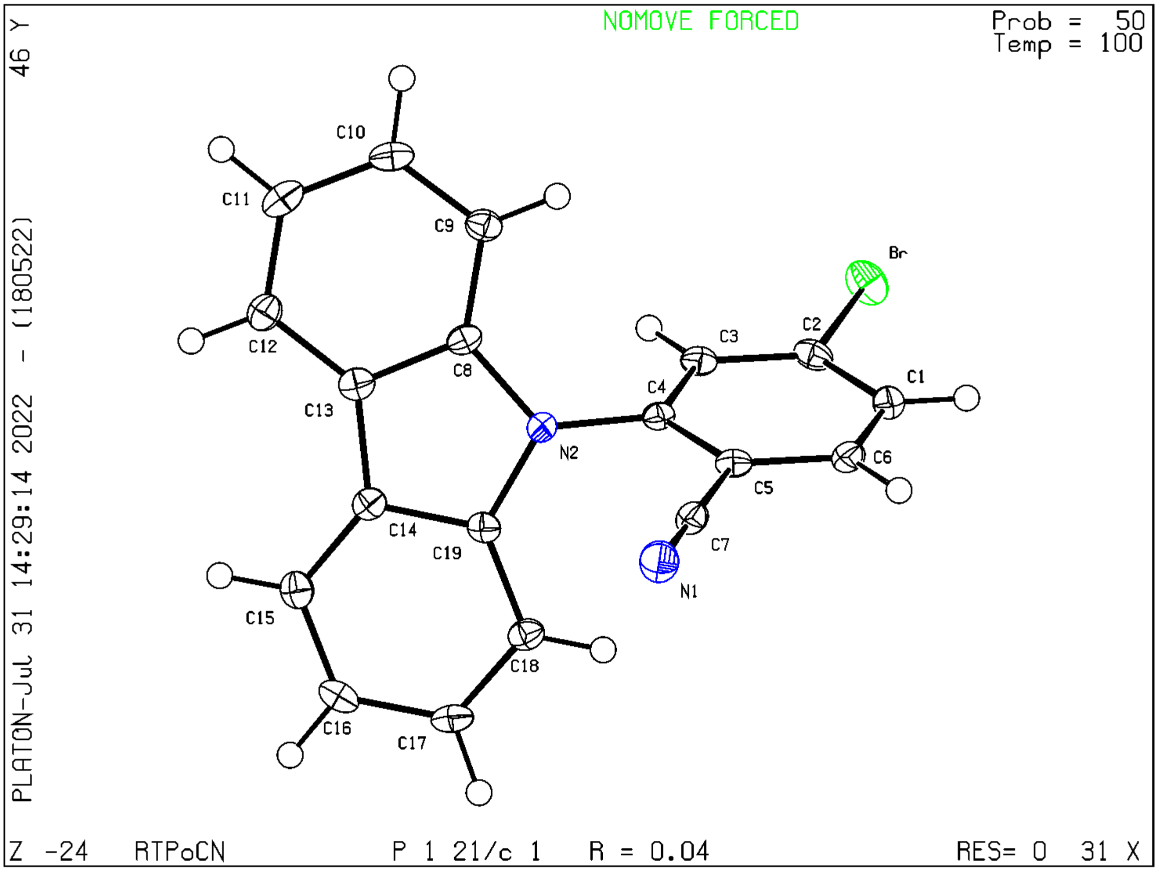

Datablock RTPoBr - ellipsoid plot

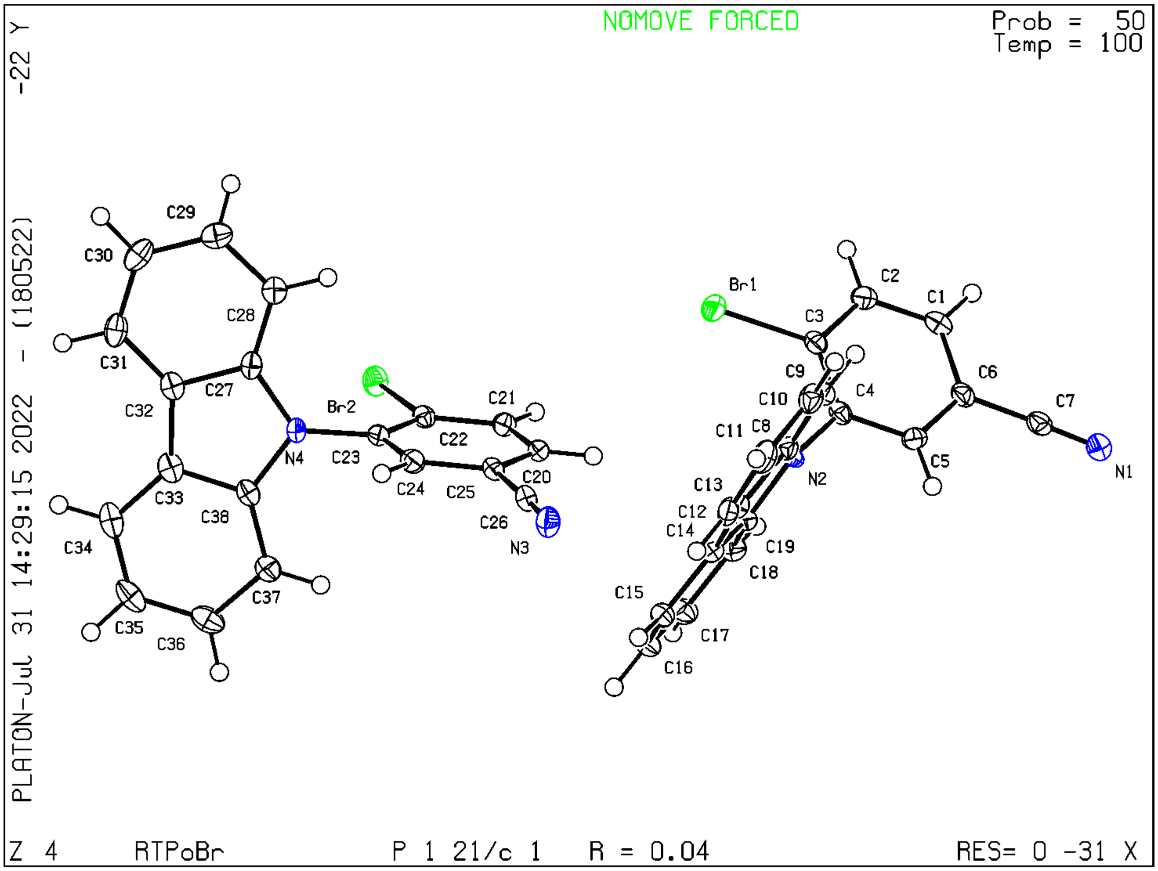

Supplement: Supplementary file 1 [file DataSheet1.PDF]
