# Supplementary material for: Organic persistent room temperature phosphorescence enabled by carbazole impurity
Source: Front Chem. 2023 Jan 6;10:1008658. doi: 10.3389/fchem.2022.1008658 (PMC9853050; doi:10.3389/fchem.2022.1008658)
Supplement: Supplementary file 2 [file DataSheet2.docx]

**Supporting Information for**

**Organic Persistent Room Temperature Phosphorescence Enhanced by Twisting and Tilting of the Carbazole Donor**

NMR spectra P2

X-ray Crystallography P10

Photophysical Characterisation P15

Computational Details P19

**Synthetic Schemes and NMR spectra:**

**Figure S1.** ^1^H NMR (500 MHz, Acetone-d_6_)

**Figure S2.** ^13^C{^1^H} NMR (126 MHz, Acetone-d_6_)


 **Figure S3.** ^1^H NMR (500 MHz, Acetone-d_6_)

**Figure S4.** ^13^C{^1^H} NMR (126 MHz, Acetone-d_6_)


 **Figure S5.** ^1^H NMR (500 MHz, Acetone-d_6_)

 **Figure S6.** ^13^C{^1^H} NMR (126 MHz, Acetone-d_6_)


 **Figure S7.** ^1^H NMR (500 MHz, Acetone-d_6_)

**Figure S8.** ^13^C{^1^H} NMR (126 MHz, Acetone-d_6_)

**X-ray Crystallography.**

**Figure S9.** Displacement ellipsoid (50%) representations of the solid state molecular structure of **RTP-Br**.


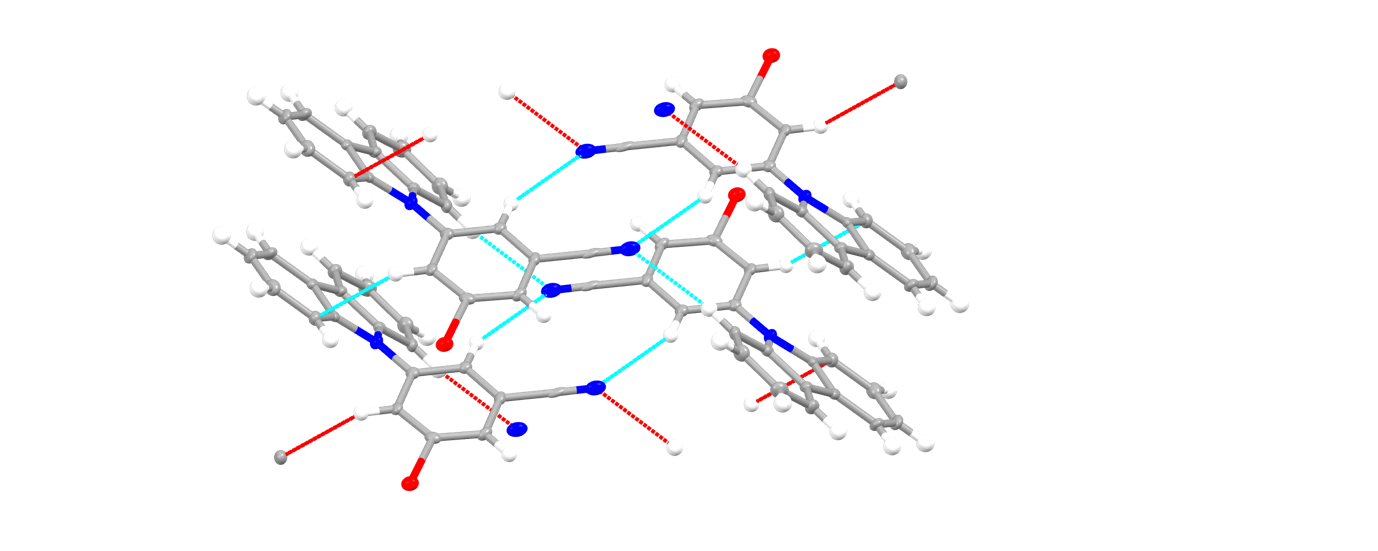


**Figure S10.** Displacement ellipsoid (50%) representations of the solid state molecular structure and packing arrangement of **RTP-Br**.

**Figure S11.** Displacement ellipsoid (50%) representations of the solid state molecular structure of **RTP-Cl**.


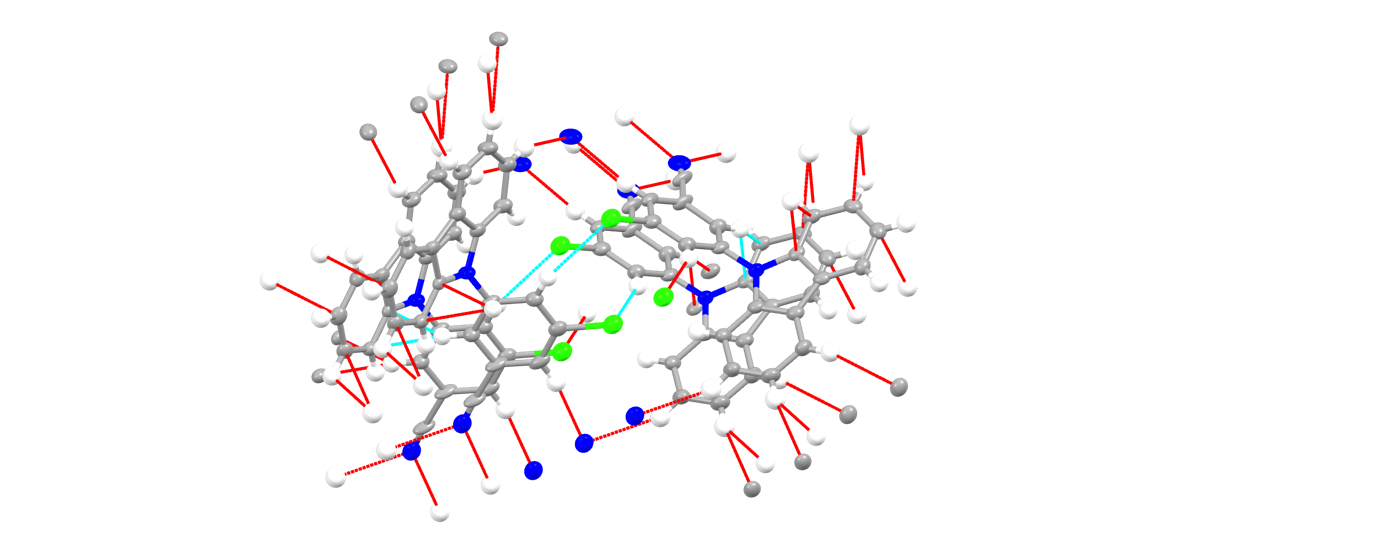


**Figure S12.** Displacement ellipsoid (50%) representations of the solid state molecular structure and packing arrangement of **RTP-Cl**.

**Figure S13.** Displacement ellipsoid (50%) representations of the solid state molecular structure of **RTP-I**.


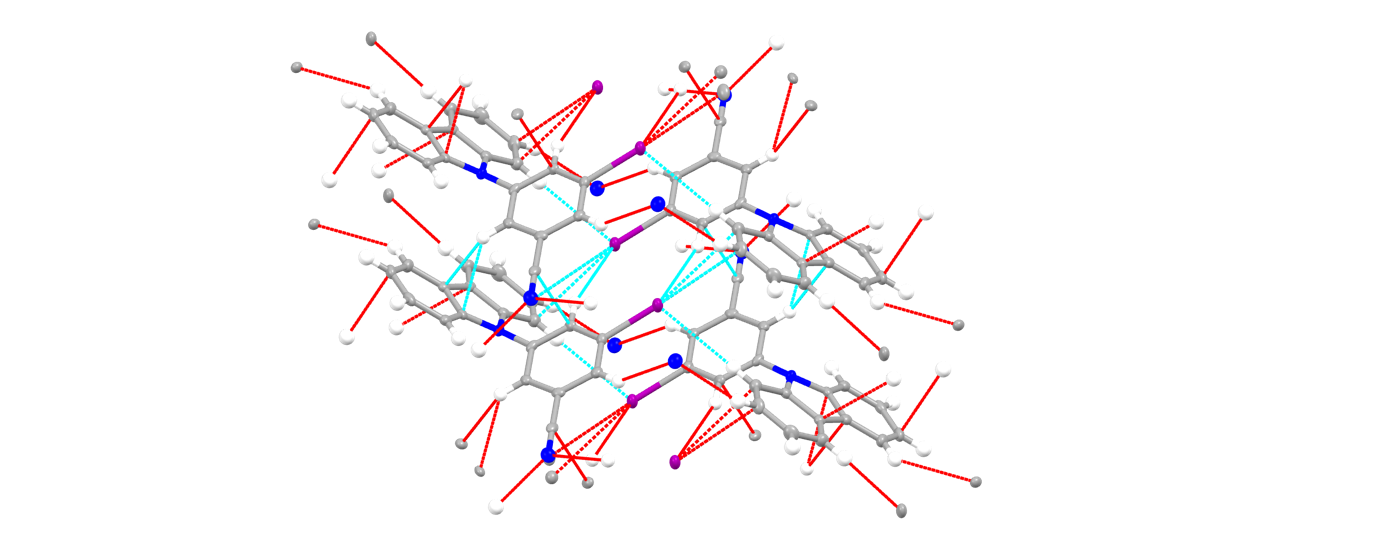


**Figure S14.** Displacement ellipsoid (50%) representations of the solid state molecular structure and packing arrangement of **RTP-I**.

**Figure S15.** Displacement ellipsoid (50%) representations of the solid state molecular structure of **RTP-*o*-CN**.


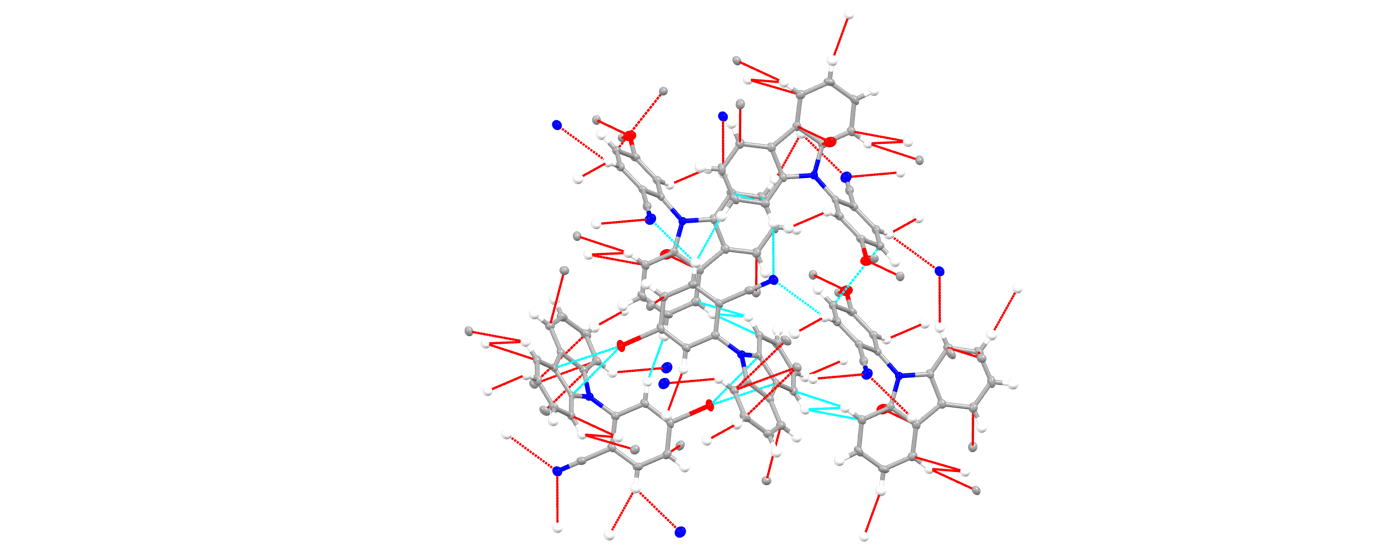


**Figure S16.** Displacement ellipsoid (50%) representations of the solid state molecular structure and packing arrangement of **RTP- *o*-CN**.

**Figure S17.** Displacement ellipsoid (50%) representations of the solid state molecular structure of **RTP-*o*-Br**.

**Photophysical Characterisation.**

**Table S1.** Photophysical properties in crystalline powder and frozen MeTHF solution.

|  | λ_em_ (nm) | τ_500nm_ (ms) | τ_550nm_ (ms) | Φ (%) | λ_em_ (nm) | τ_460nm_ (ms) | τ_560nm_ (ms) |
| --- | --- | --- | --- | --- | --- | --- | --- |
| Crystalline Powder (295K) | | | | | MeTHF (77K) | | |
| **RTP-Br** | 552 | 0.65 (39%)  3.51 (31%)  27.9 (30%) | 19.9 (11%)  216 (89%) | 20 | 438 | 40.0 (21%)  217 (20%)  2434 (59%) | - |
| **RTP-Cl** | 413 | 1.71 (44%)  22.6 (56%) | 14.6 (23%)  406 (77%) | 6 | 438 | 744 (17%)  4227 (83%) | - |
| **RTP-I** | 564 | - | 16.9 (78%)  163 (22%) | 7 | 438 | 10.9 (24%)  105 (23%)  1517 (53%) | - |
| **RTP-*o*-CN** | 563 | - | 106 (41%)  293 (59%) | 22 | 437 | 203 (4%)  3060 (96%) | - |
| **RTP-*o*-Br** | 540 | 4.78 (29%)  127 (71%) | 56.6 (6%)  371 (94%) | 6 | 409 | 16.3 (6%)  180 (21%)  670 (73%) | 8.27 (29%)  61.2 (34%)  410 (37%) |

**Figure S18.** Photoluminescence spectra of **RTP-Br** (commercial carbazole with Bz admixture) in crystalline powder (295K, left) and frozen MeTHF solution (77K, right) with emission and excitation profiles.

**Figure S19.** Photoluminescence spectra of **RTP-Cl** (commercial carbazole with Bz admixture) in crystalline powder (295K, left) and frozen MeTHF solution (77K, right) with emission and excitation profiles.

**Figure S20.** Photoluminescence spectra of **RTP-I** (commercial carbazole with Bz admixture) in crystalline powder (295K, left) and frozen MeTHF solution (77K, right) with emission and excitation profiles.

**Figure S21.** Photoluminescence spectra of **RTP-*o*-CN** (commercial carbazole with Bz admixture) in crystalline powder (295K, left) and frozen MeTHF solution (77K, right) with emission and excitation profiles.

**Figure S22.** Photoluminescence spectra of **RTP-*o*-Br** (commercial carbazole with Bz admixture) in crystalline powder (295K, left) and frozen MeTHF solution (77K, right) with emission and excitation profiles.

**Figure S23.** Photoluminescence spectra of **RTP-Br** (laboratory synthesized carbazole without Bz admixture) in crystalline powder (295K, left) and frozen MeTHF solution (77K, right) with emission and excitation profiles.

**Figure S24.** Photoluminescence spectra of **RTP-o-Br** (laboratory synthesized carbazole without Bz admixture) in crystalline powder (295K, left) and frozen MeTHF solution (77K, right) with emission and excitation profiles.

**Computational Details**

1. **Crystal structure geometry**

**Table S2.** Molecular-orbital distribution of the HOMO and LUMO for pRTP materials.

|  | HOMO | LUMO |
| --- | --- | --- |
| 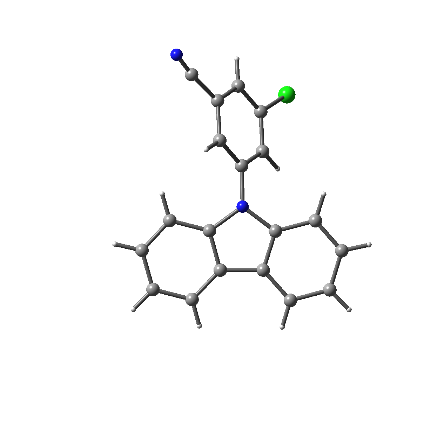  RTP-Cl  Overlap integral: 0.41 | 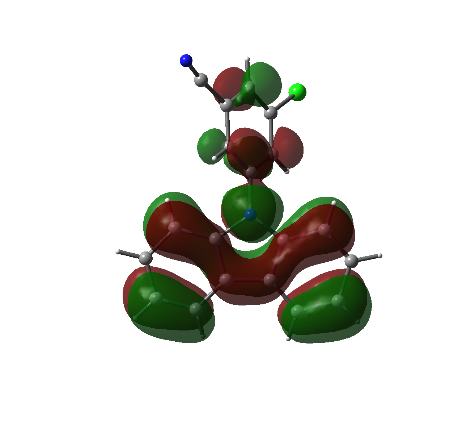 | 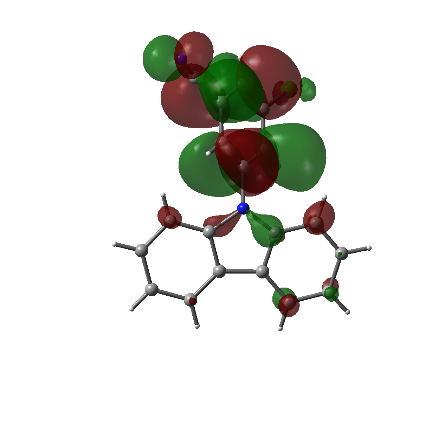 |
| 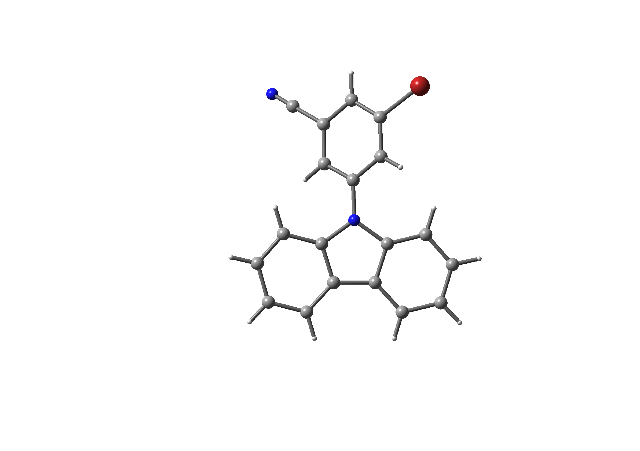  RTP-Br  Overlap integral: 0.43 | 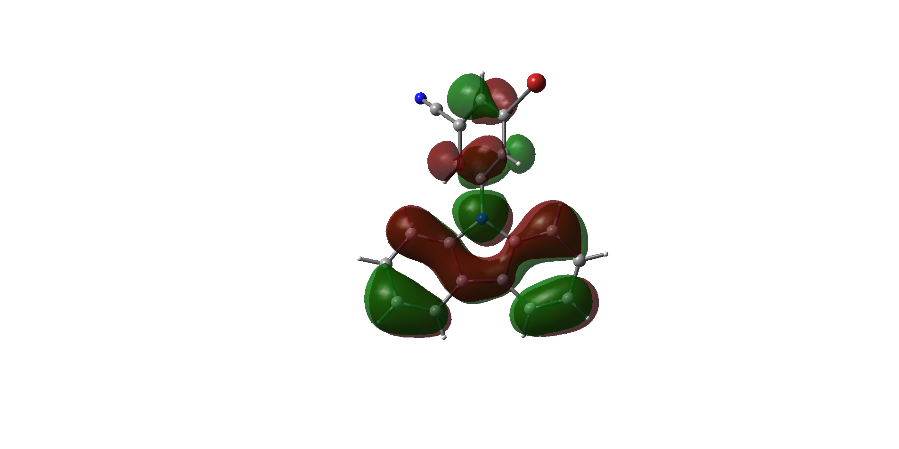 | 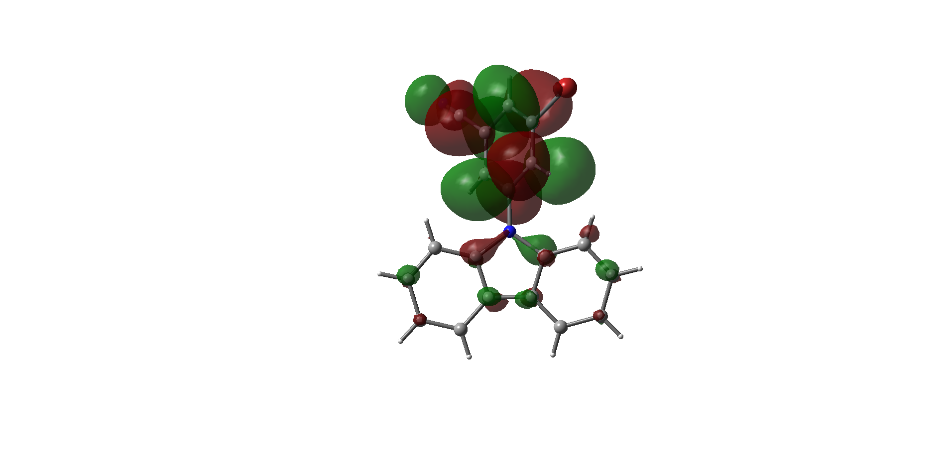 |
| 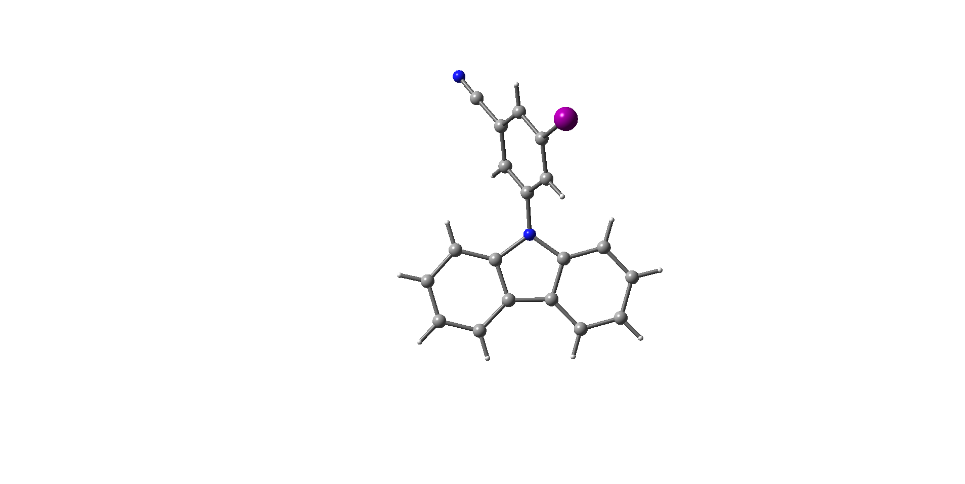  RTP-I  Overlap integral: 0.35 | 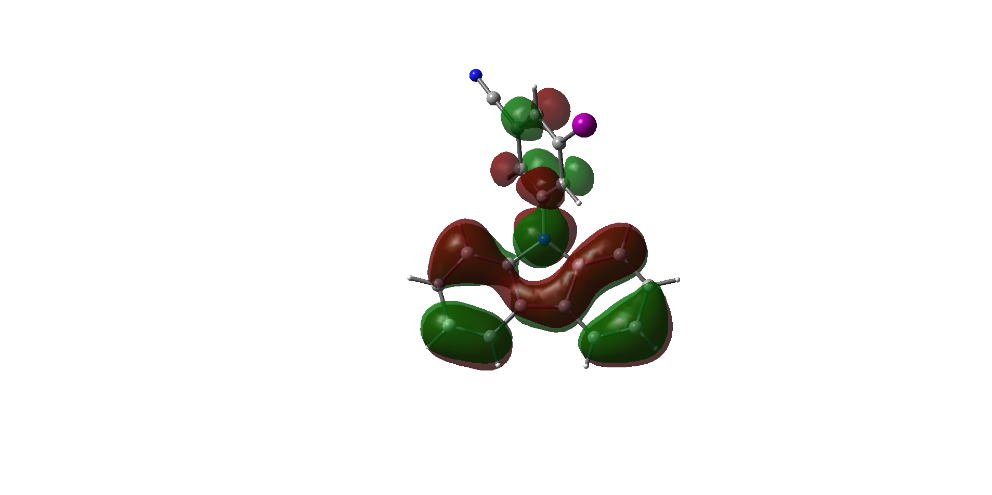 | 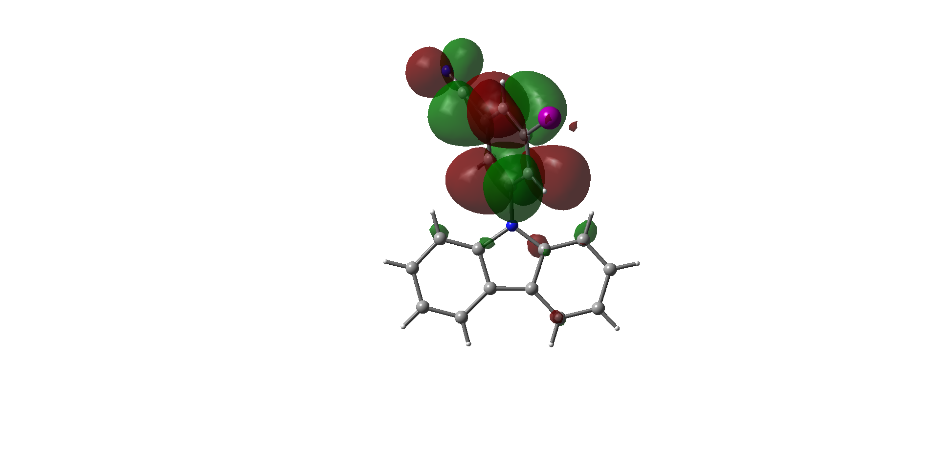 |
| 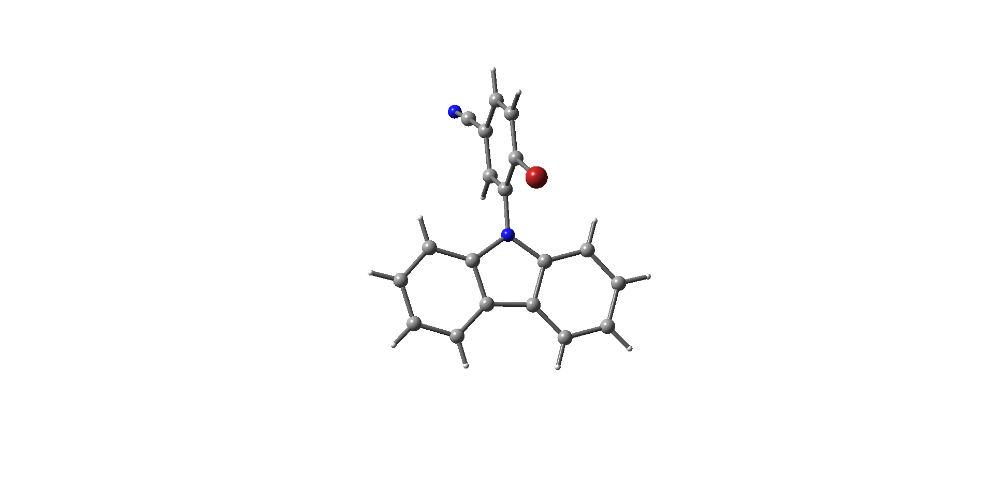  RTP-o-Br  Overlap integral: 0.17 | 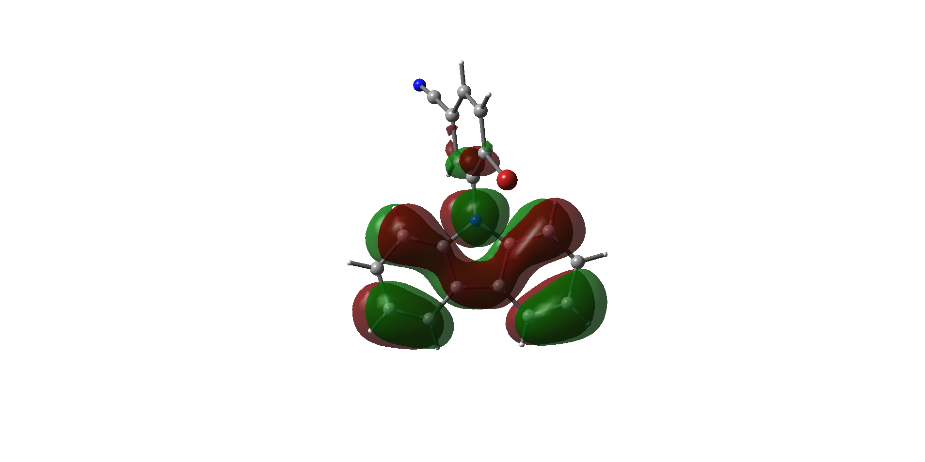 | 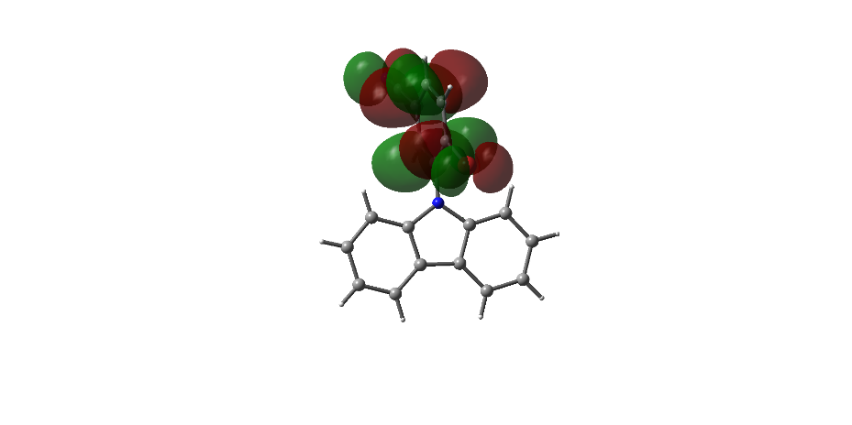 |
| 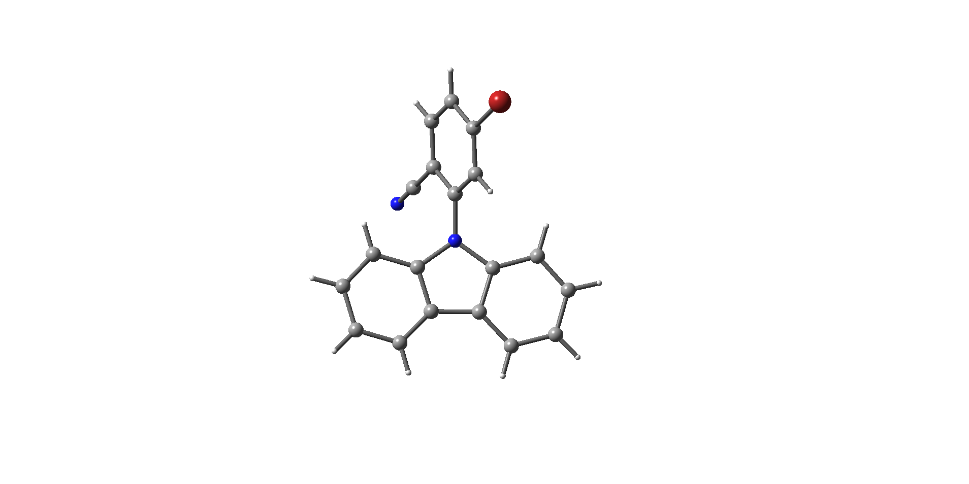  RTP-o-CN  Overlap integral: 0.28 | 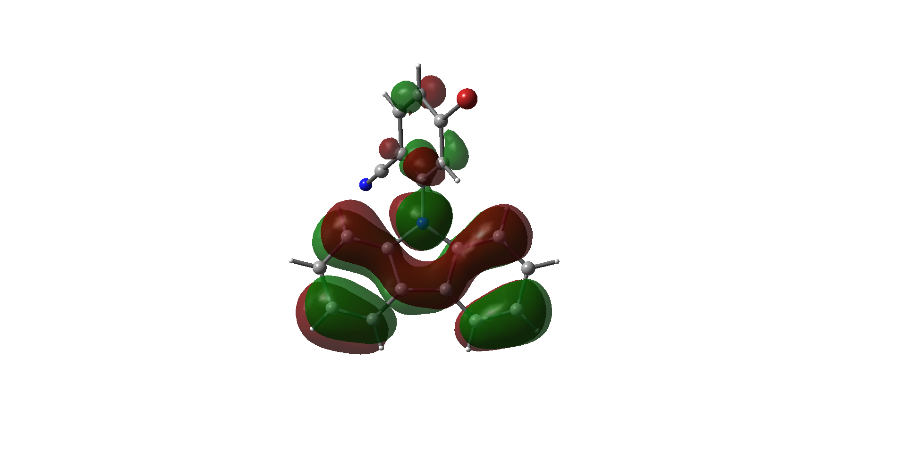 | 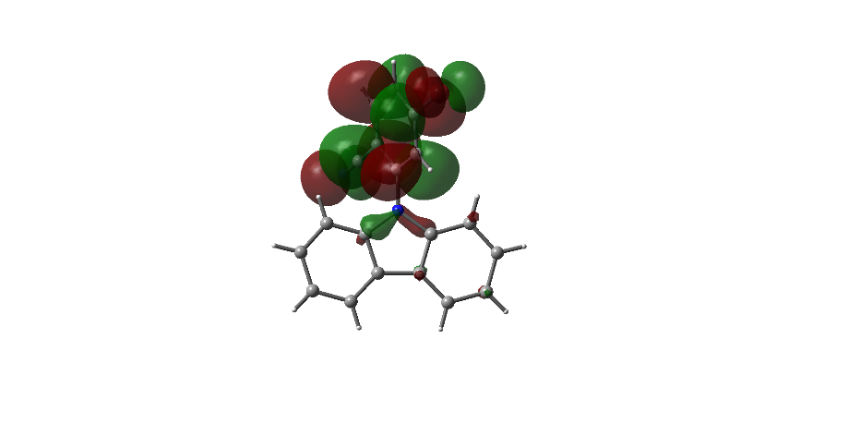 |

**Table S3.** Dipole moments for RTP materials in crystalline geometry for ground S_0_ and lowest vertical excitation S_1._

|  | Crystal structure | S1 |
| --- | --- | --- |
| RTP-Cl | 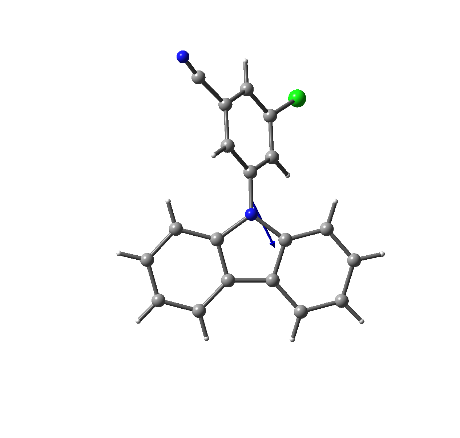  (-)3.6D | 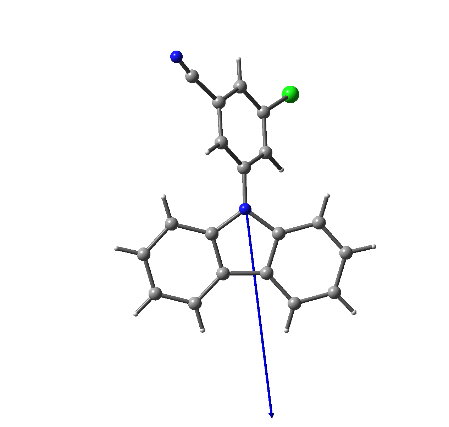  (-)14.0D |
| RTP-Br | 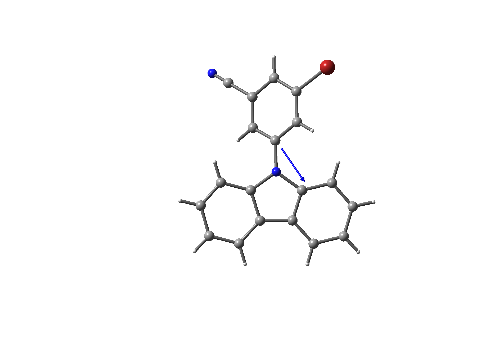  (-)3.7D | 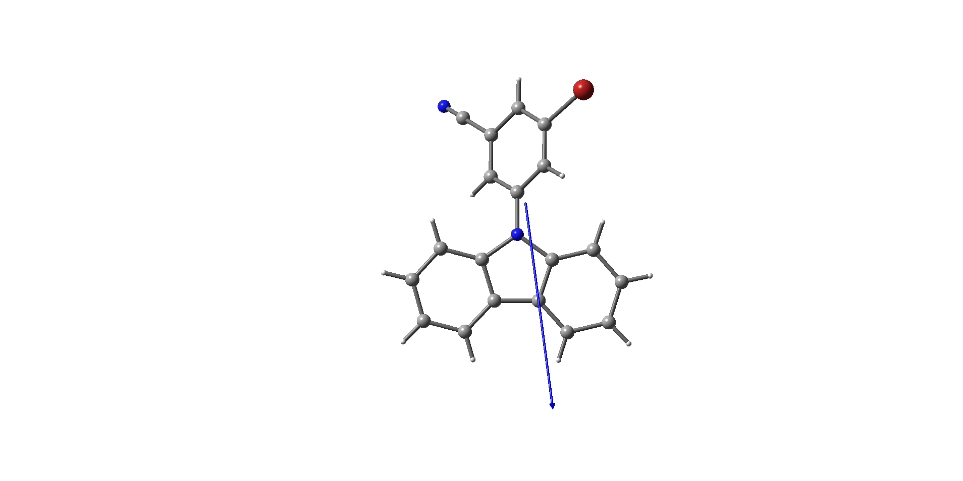  (-)13.1D |
| RTP-I | 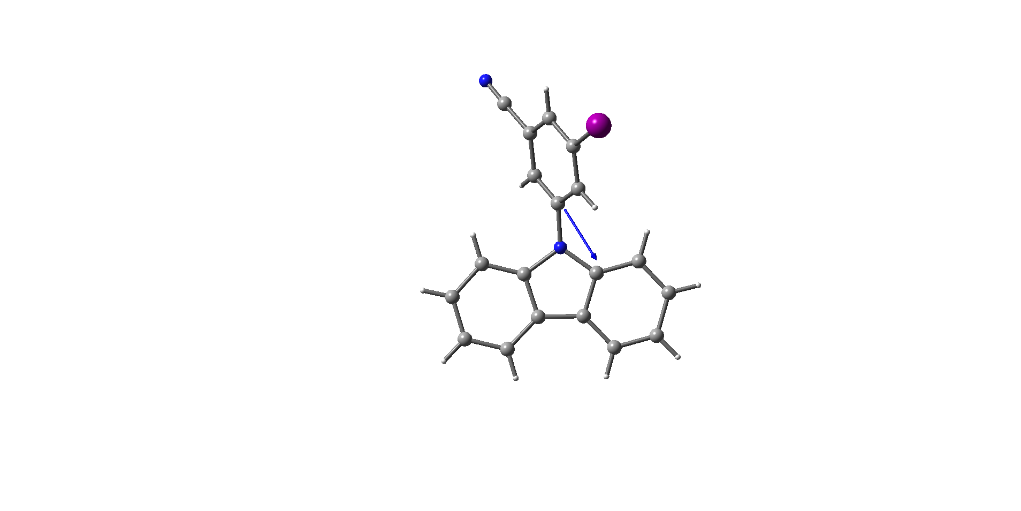  (-)3.8D | 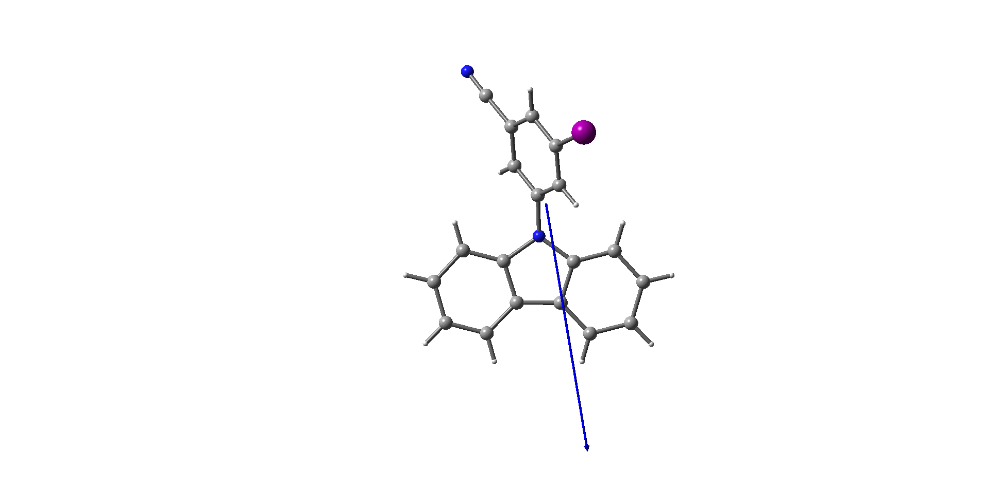  (-)15.0D |
| RTP-o-Br | 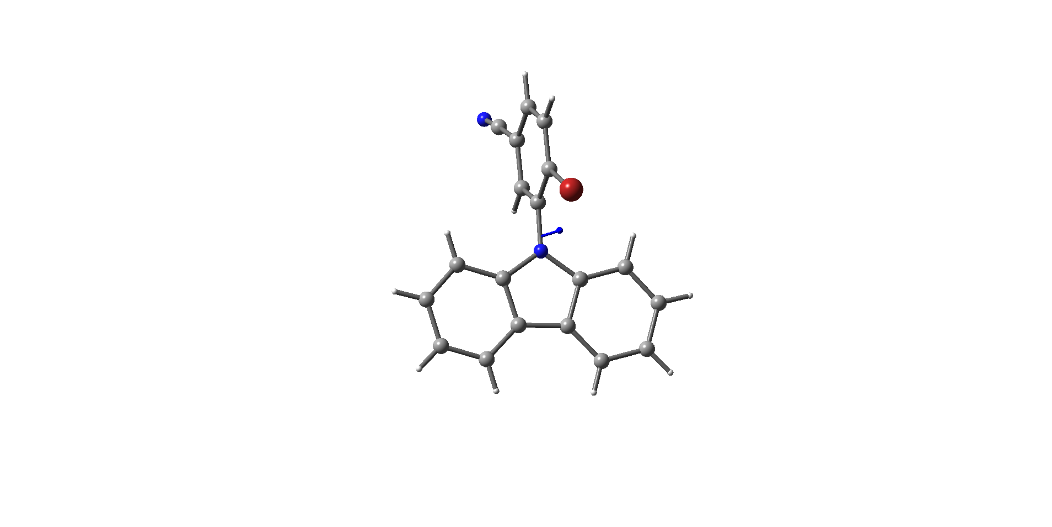  3.0D | 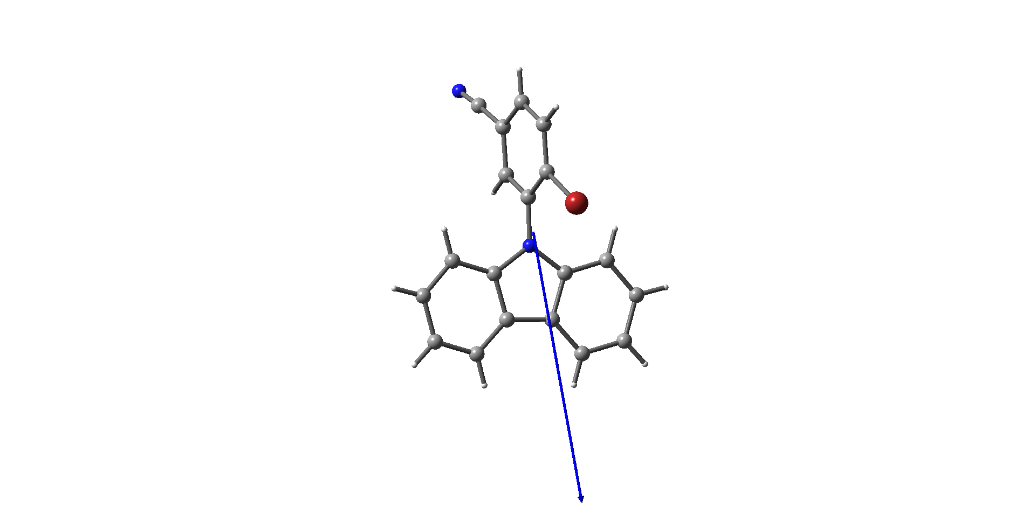  (-)14.9D |
| RTP-o-CN | 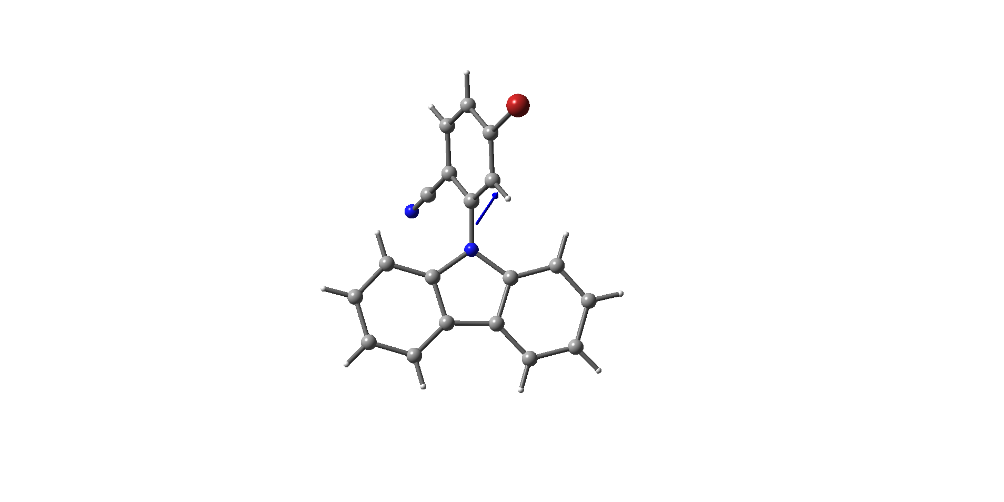  3.2D | 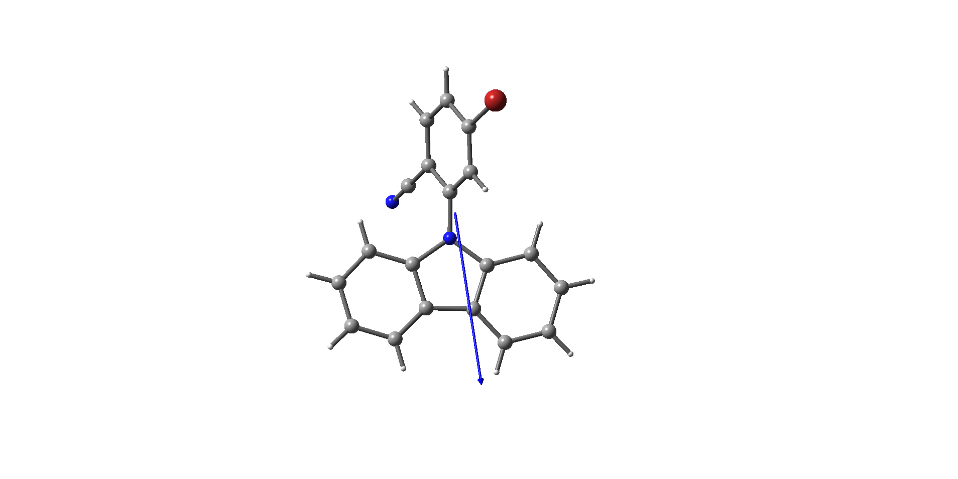  (-)10.3D |

**Table S4.** Excited state energies and orbital contributions to vertical excitation and oscillator strength coefficients.

|  | Excitation energy | Character | Oscillator strength |
| --- | --- | --- | --- |
| RTP-Cl | S0→S1; (^1^CT): 4.04eV = 307nm | HOMO – LUMO (95%) | 0.0447 |
|  | S0→T1; (mix ^3^CT and ^3^LE(phenyl)): 3.52eV = 353nm |  |  |
|  | S0→T2; (^3^LE(Cz)): 3.60eV = 345nm |  |  |
| RTP-Br | S0→S1; (^1^CT): 4.14eV = 300nm | HOMO – LUMO (91%) | 0.1109 |
|  | S0→T1; (^3^LE(Cz)): 3.45eV = 359nm |  |  |
|  | S0→T2; (mix ^3^CT and ^3^LE(phenyl)): 3.56eV = 349nm |  |  |
| RTP-I | S0→S1; (^1^CT): 3.96eV = 313nm | HOMO – LUMO (94%) | 0.0241 |
|  | S0→T1; (^3^LE(Cz)): 3.42eV = 362nm |  |  |
|  | S0→T2; (mix ^3^CT and ^3^LE(phenyl)): 3.53eV = 352nm |  |  |
| RTP-o-Br | S0→S1; (^1^CT): 3.77eV = 329nm | HOMO – LUMO (98%) | 0.0012 |
|  | S0→T1; (^3^LE(Cz)): 3.52eV = 352nm |  |  |
|  | S0→T2; (^3^LE(Cz)): 3.63eV = 342nm |  |  |
|  | S0→T3; (mix ^3^CT and ^3^LE(phenyl)): 3.72eV = 333nm |  |  |
| pRTP-o-CN | S0→S1; (^1^CT): 3.79eV = 327nm | HOMO – LUMO (93%) | 0.0569 |
|  | S0→T1; (mix ^3^CT and ^3^LE(phenyl)): 3.51eV = 353nm |  |  |
|  | S0→T2; (^3^LE(Cz)): 3.55eV = 349nm |  |  |


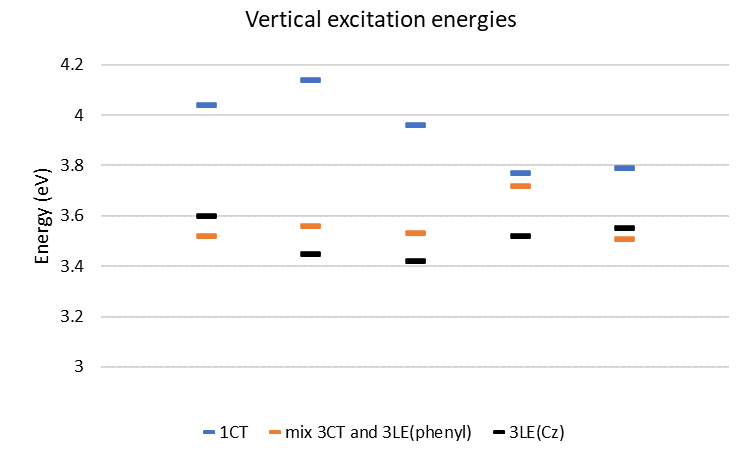


**1_Cl 1_Br 1_I o_Br o_CN**

**Table S5.** Natural transition orbitals (NTOs) of triplet excited states.

|  |  | HONTO | LUNTO |
| --- | --- | --- | --- |
| RTP-Cl | S0→T1 | 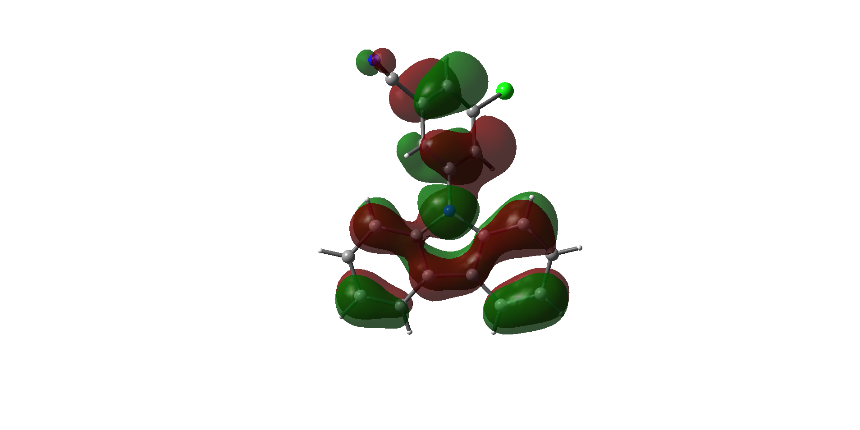 | 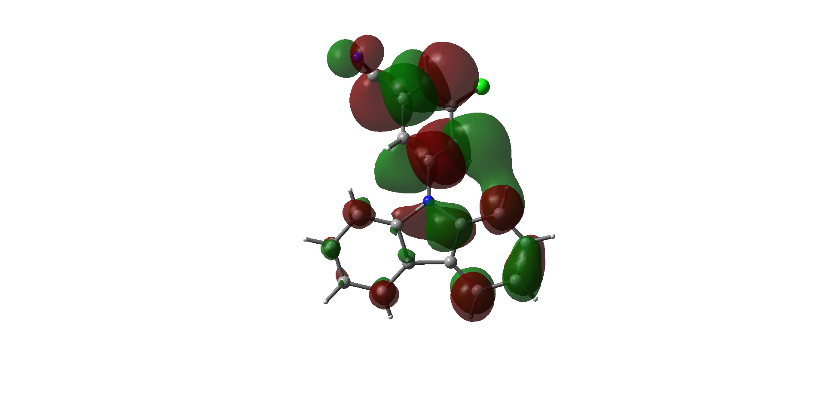 |
|  | S0→T2 | 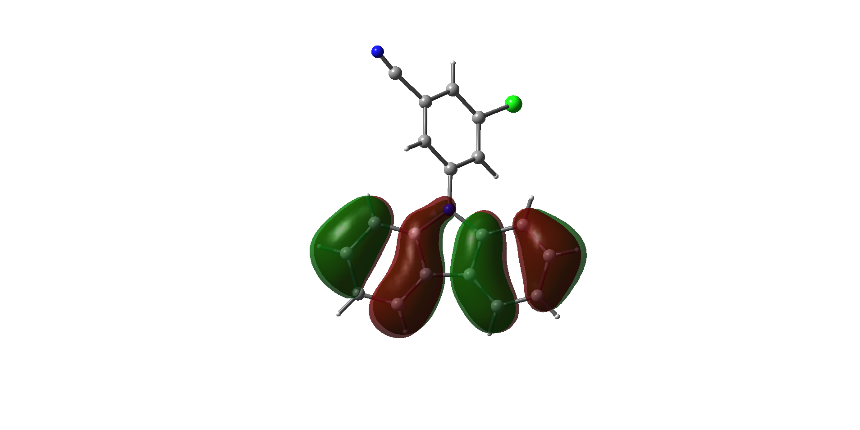 | 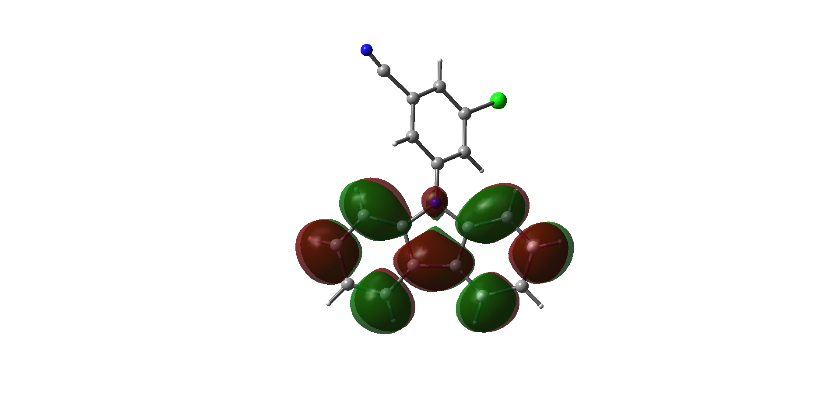 |
| RTP-Br | S0→T1 | 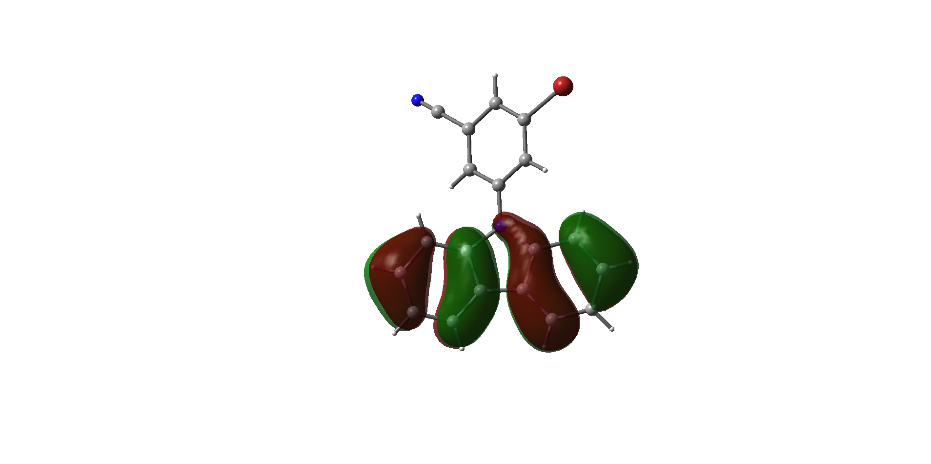 | 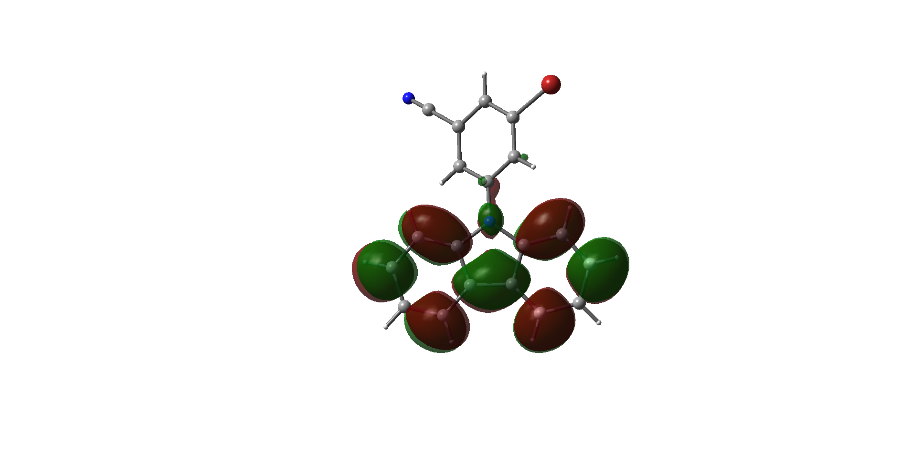 |
|  | S0→T2 | 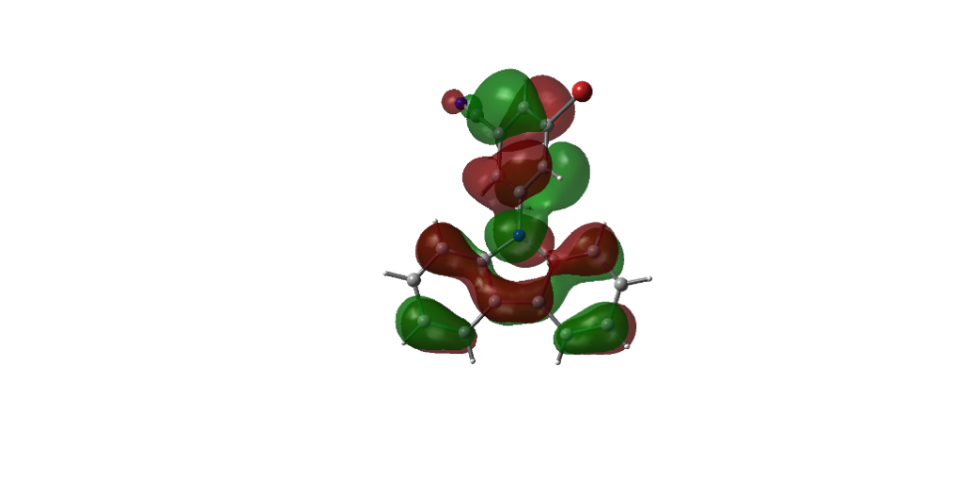 | 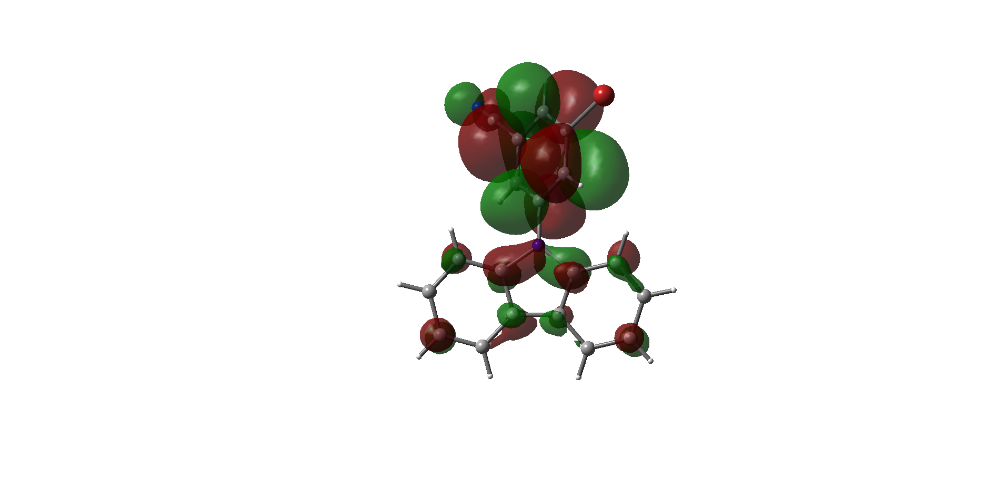 |
| RTP-I | S0→T1 | 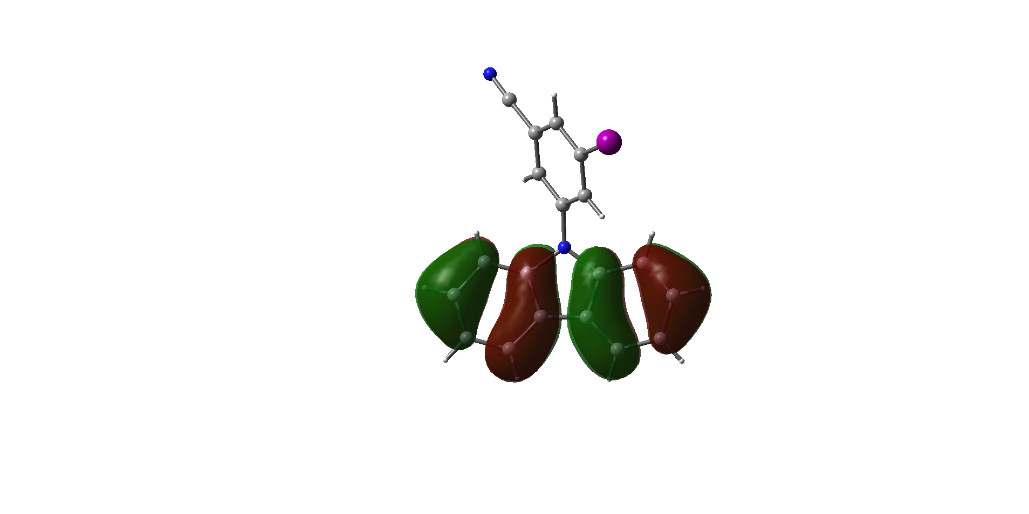 | 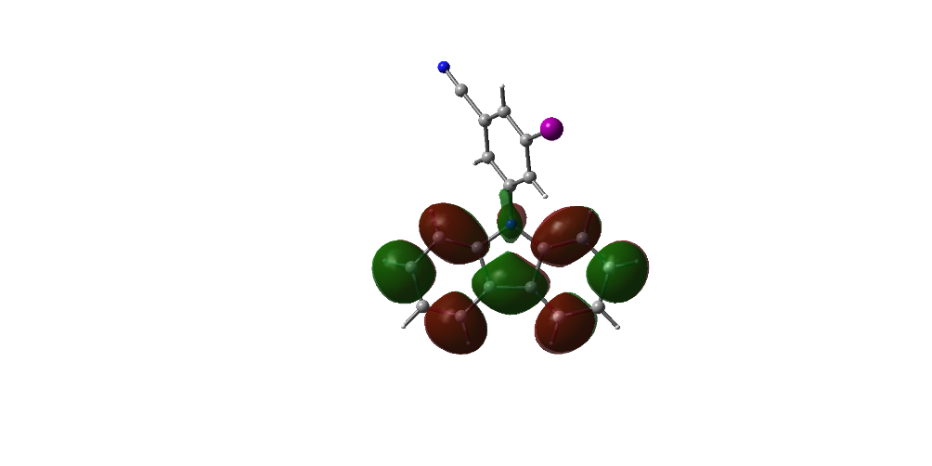 |
|  | S0→T2 | 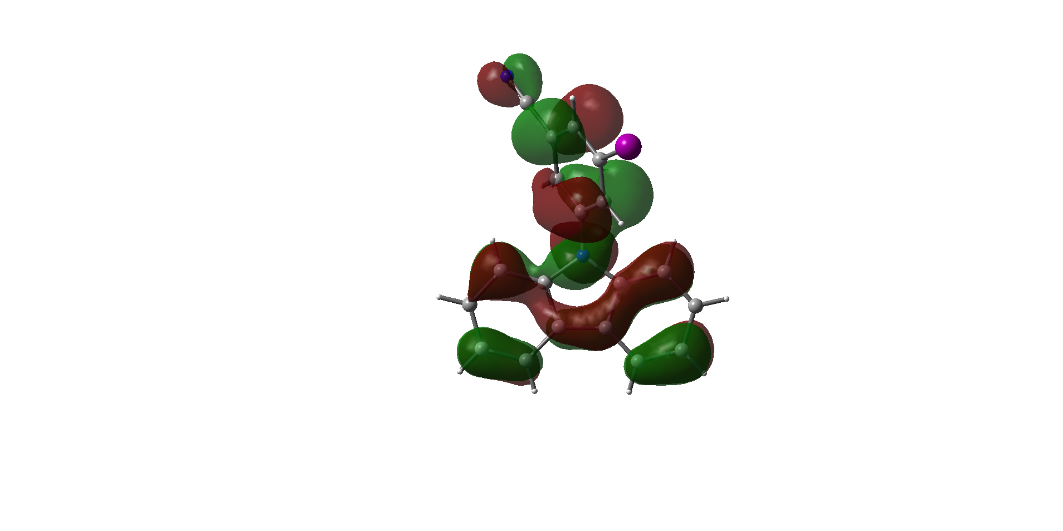 | 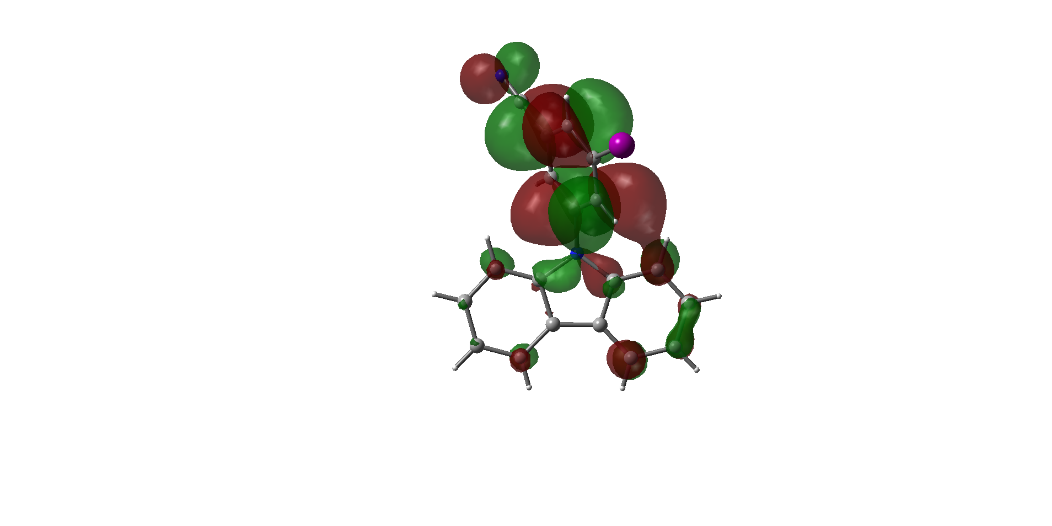 |
| RTP-o-Br | S0→T1 | 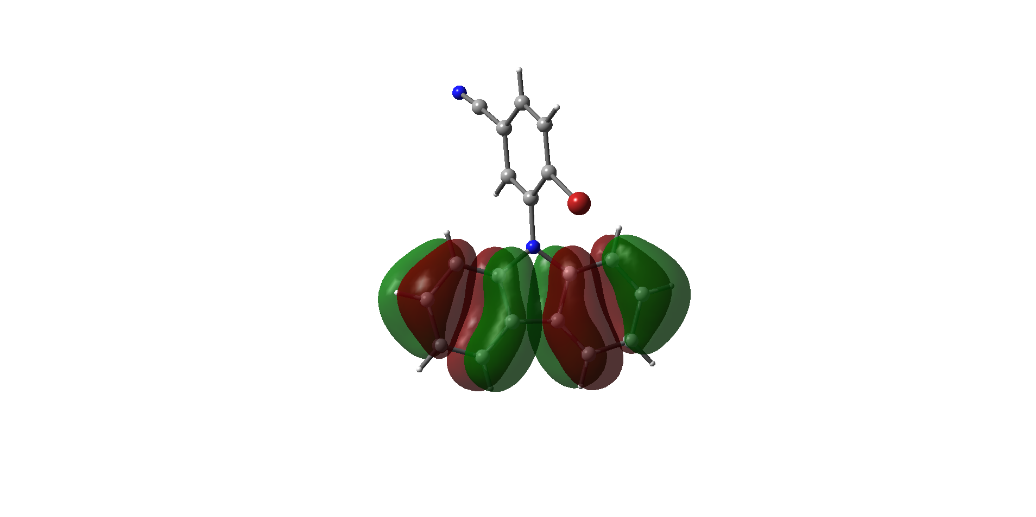 | 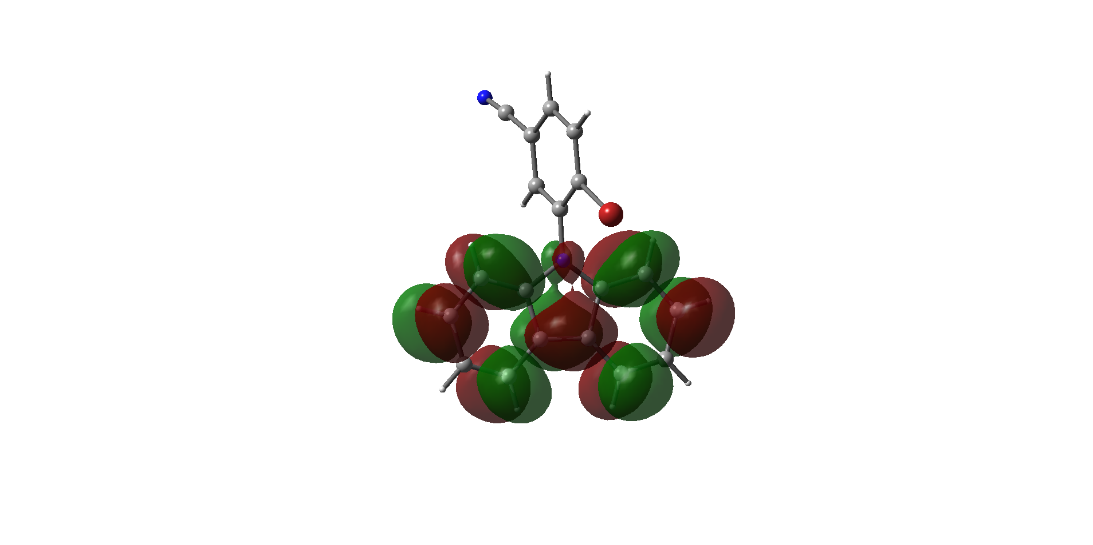 |
|  | S0→T2 | 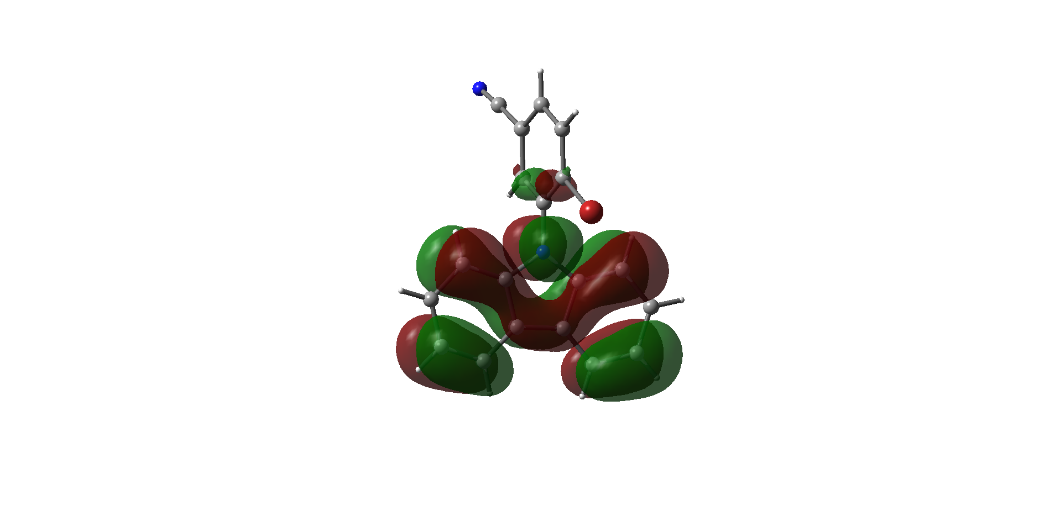 | 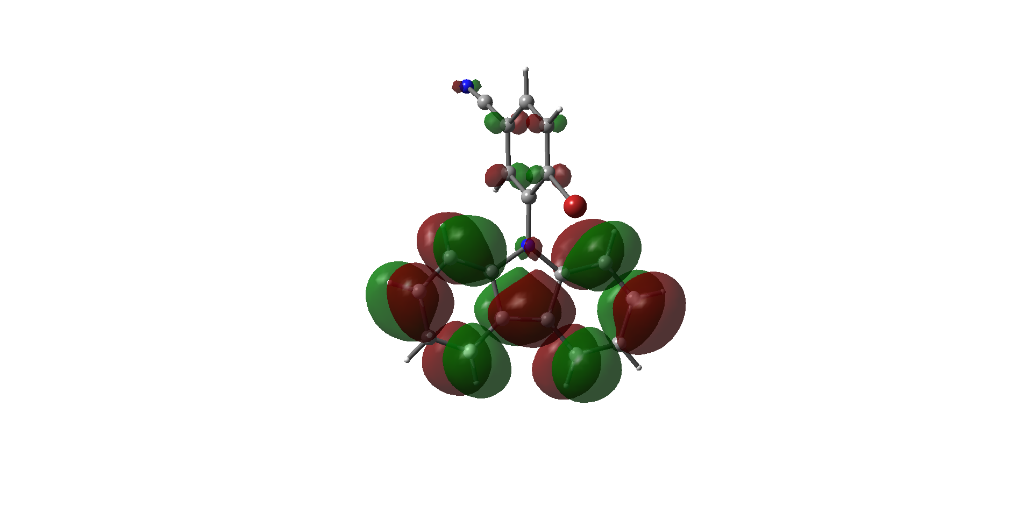 |
|  | S0→T3 | 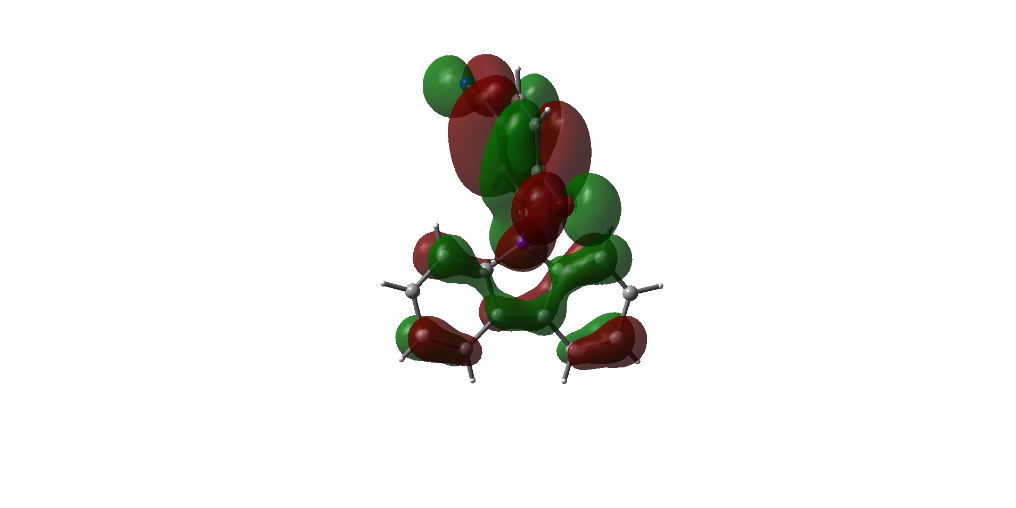 | 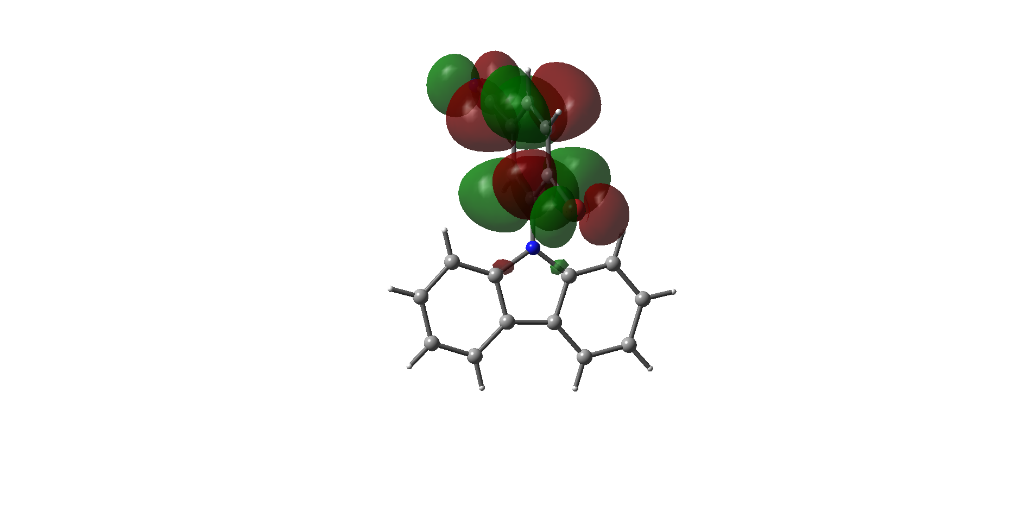 |
| RTP-o-CN | S0→T1 | 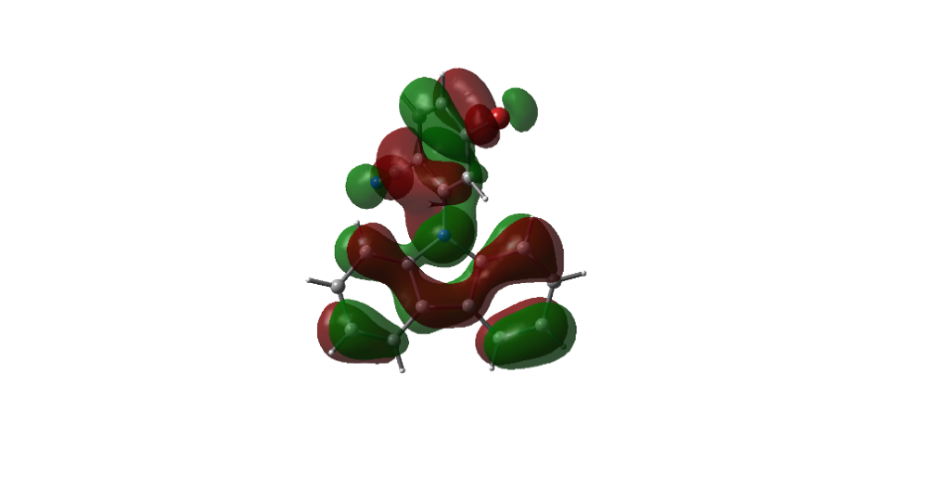 | 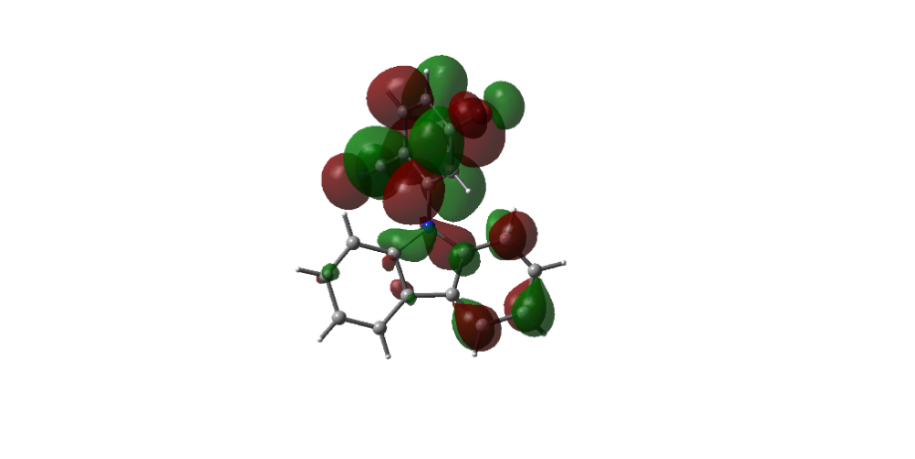 |
|  | S0→T2 | 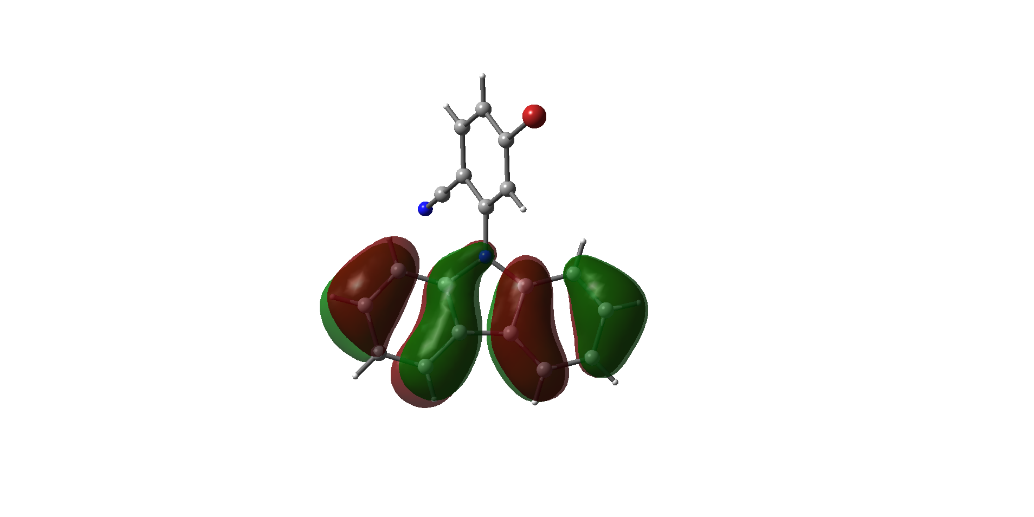 | 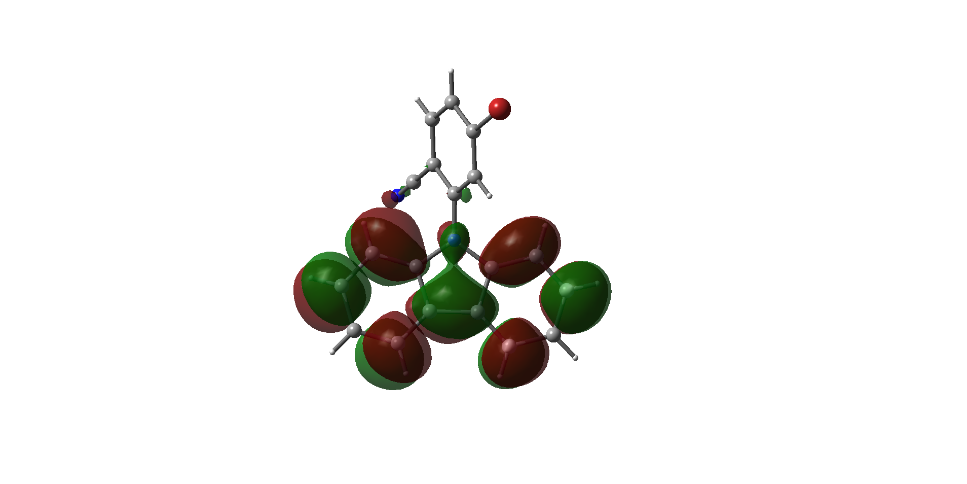 |

1. **Optimized S0 geometry**

**Table S6.** Molecular-orbital distribution of the HOMO and LUMO for RTP materials in optimized geometry.

|  | HOMO | LUMO |
| --- | --- | --- |
| 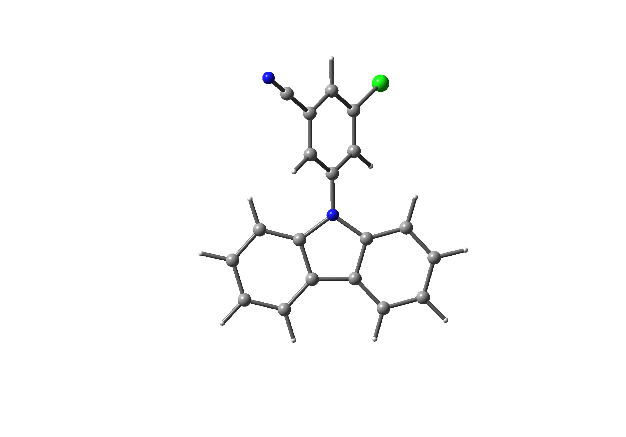  RTP-Cl  Overlap integral: 0.35 | 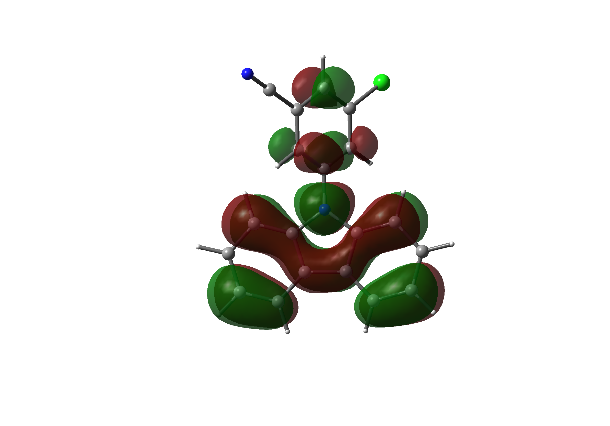 | 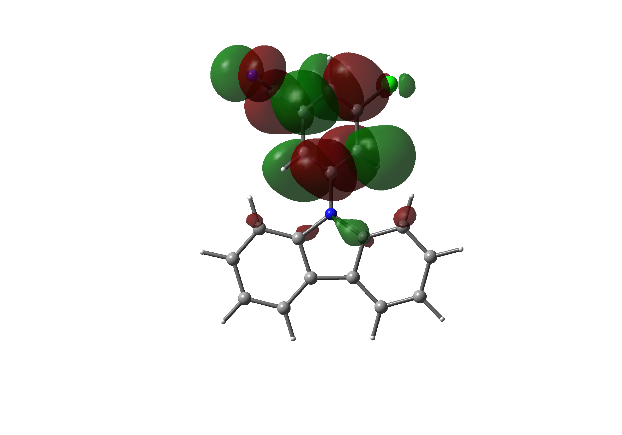 |
| 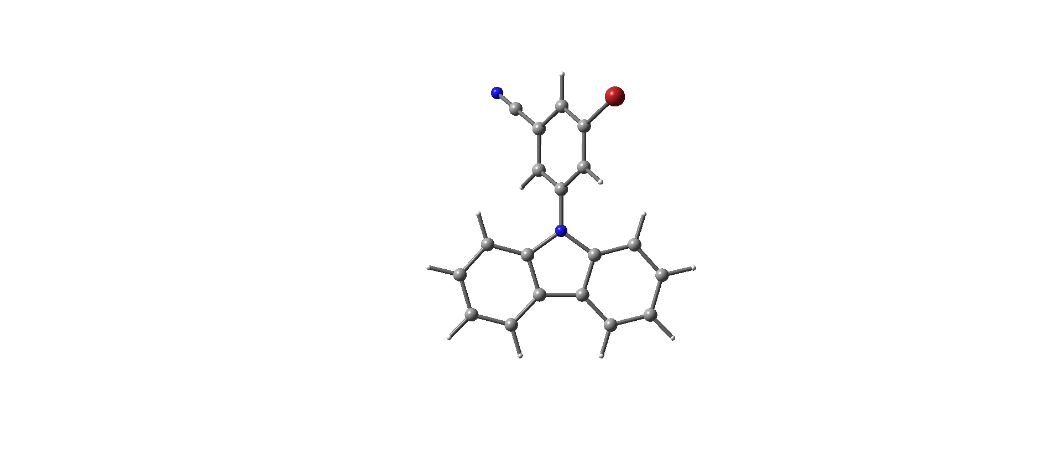  RTP-Br  Overlap integral: 0.35 | 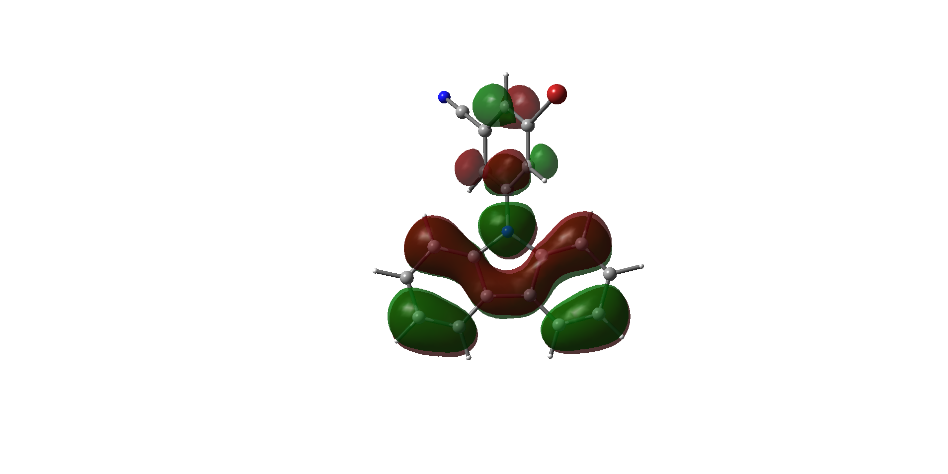 | 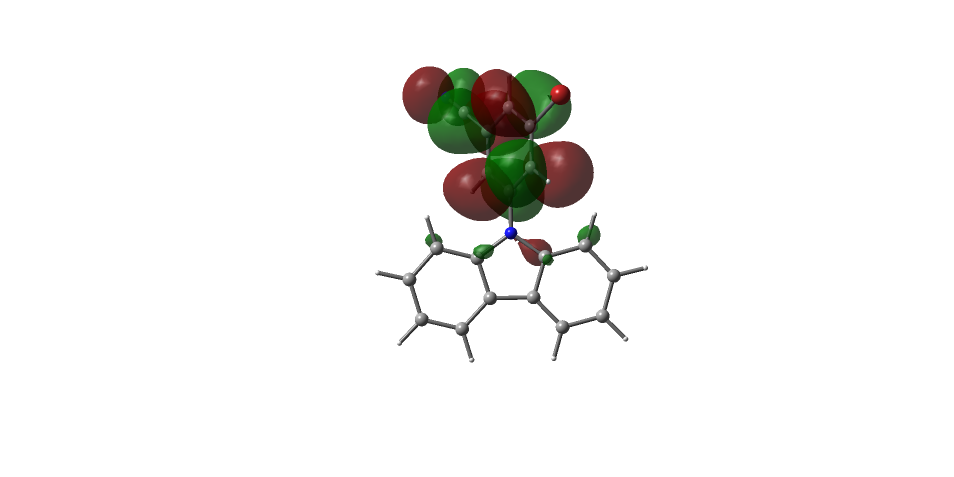 |
| 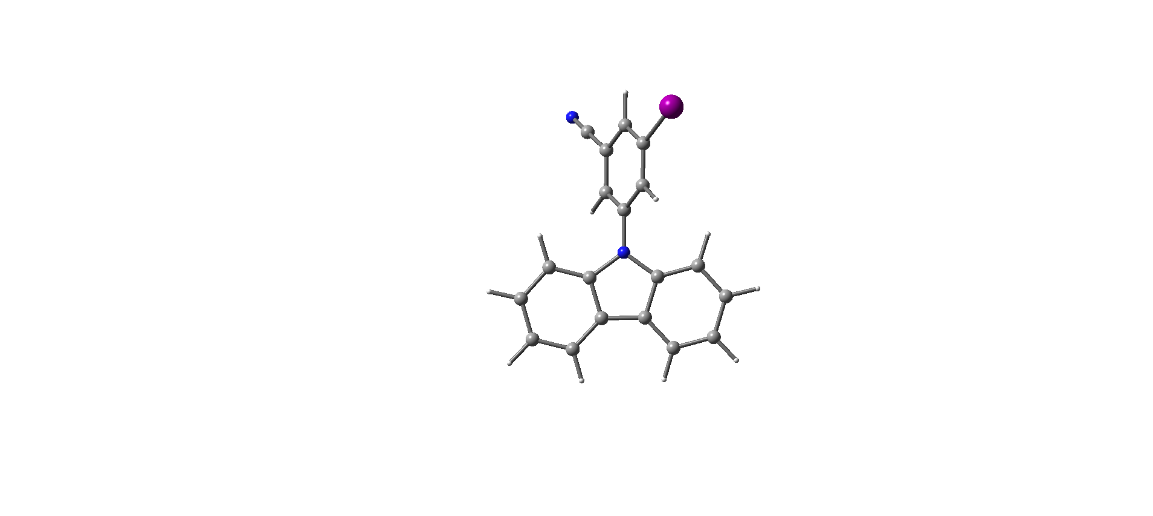  RTP-I  Overlap integral: 0.35 | 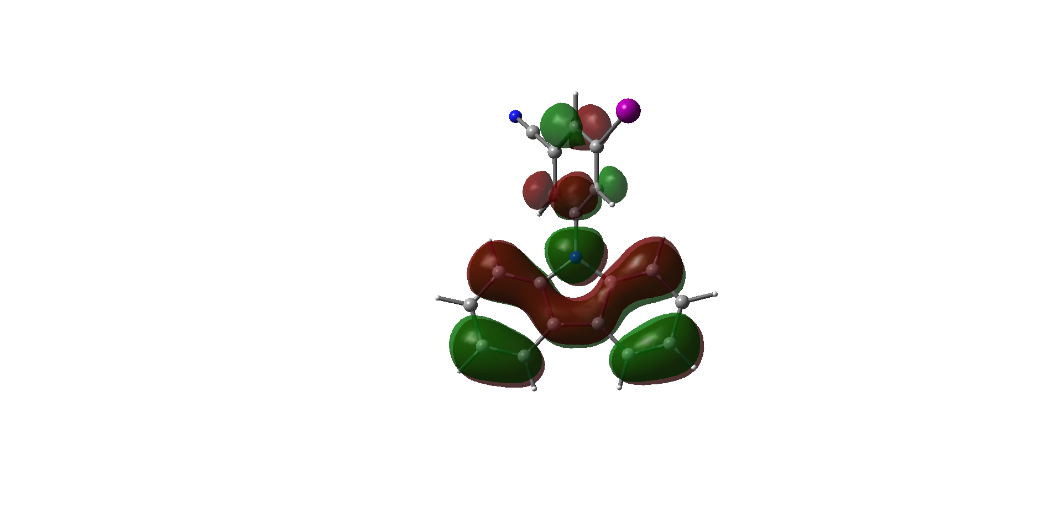 | 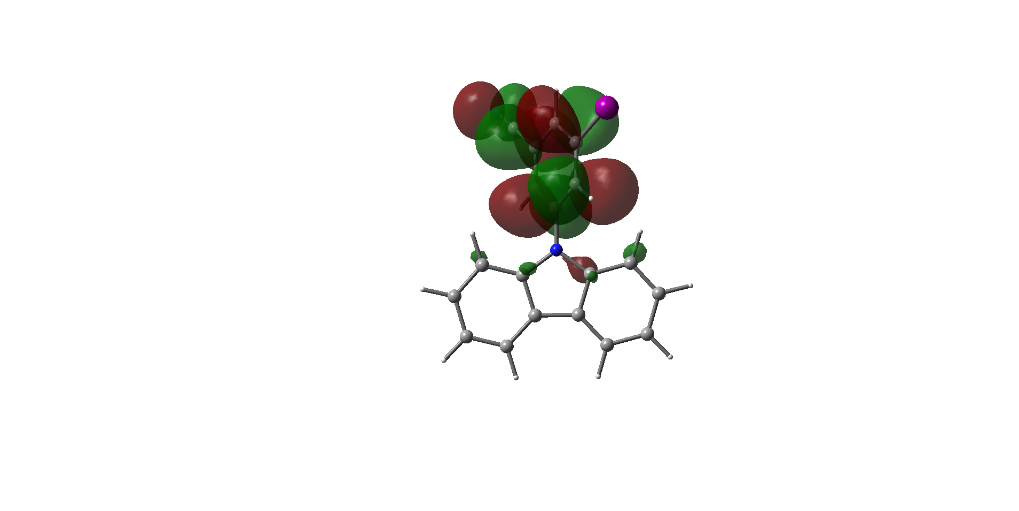 |
| 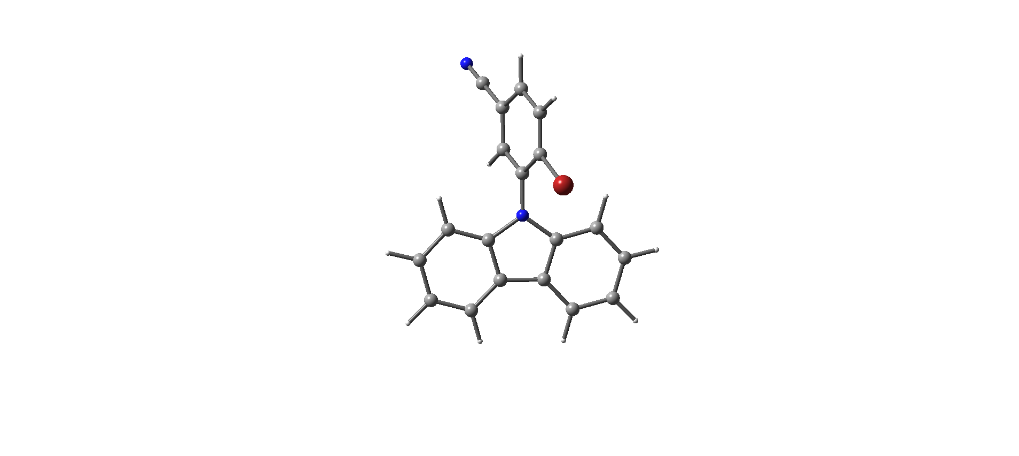  RTP-o-Br  Overlap integral: 0.26 | 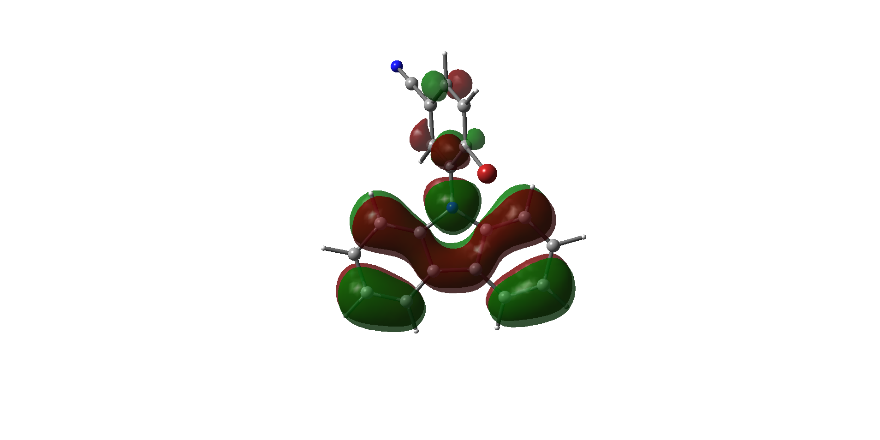 | 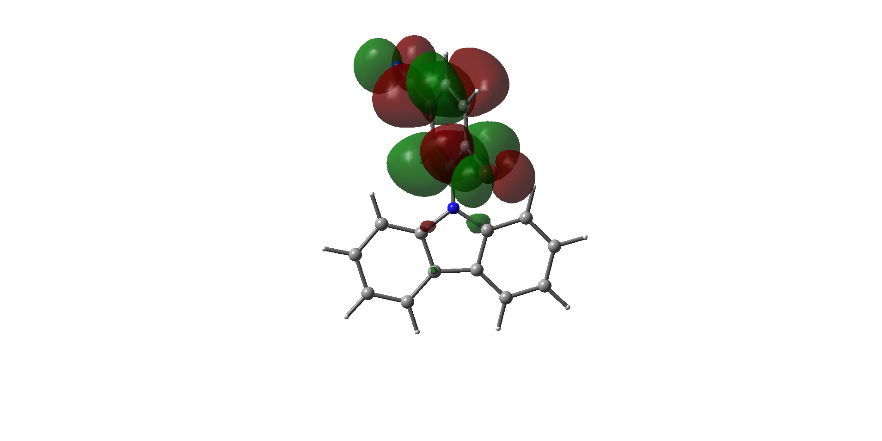 |
| 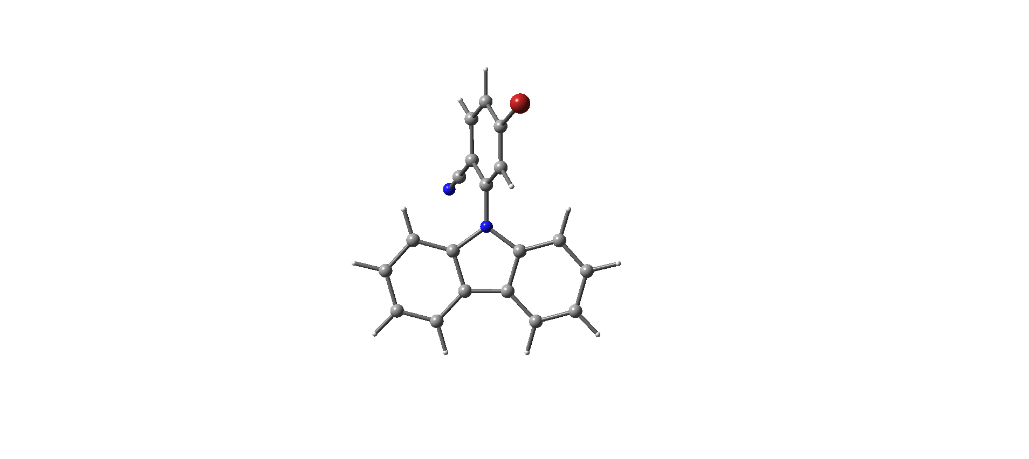  RTP-o-CN  Overlap integral: 0.33 | 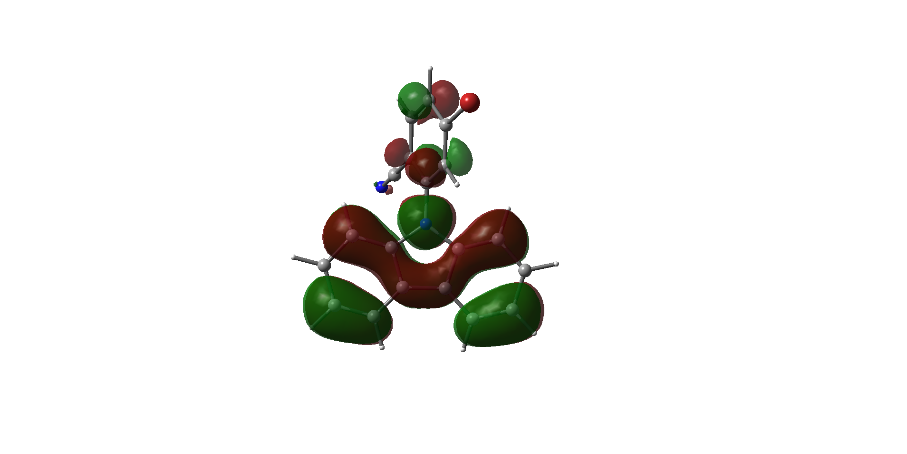 | 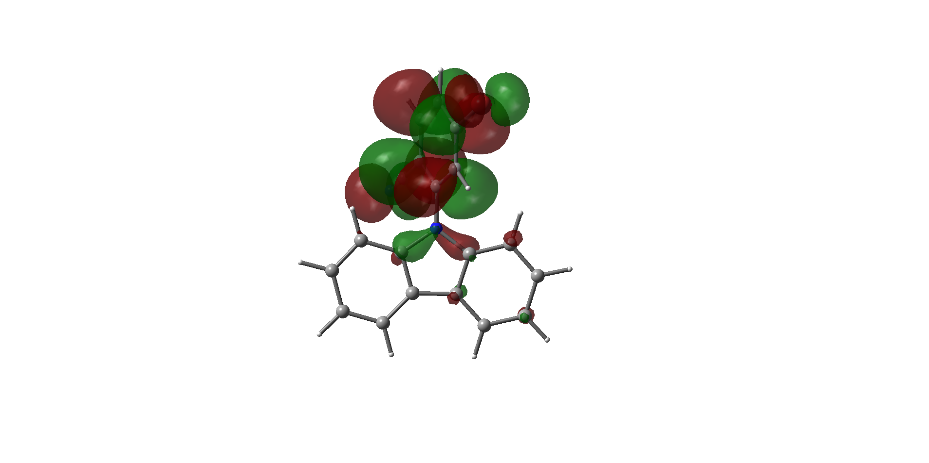 |

**Table S7.** Dipole moments for pRTP materials in optimized geometry for ground S_0_ and lowest vertical excitation S_1._

|  | S0 | S1@S0 |
| --- | --- | --- |
| RTP-Cl | 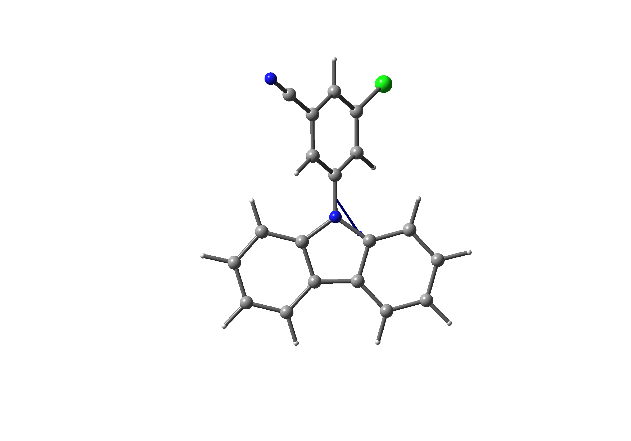  (-)3.5D | 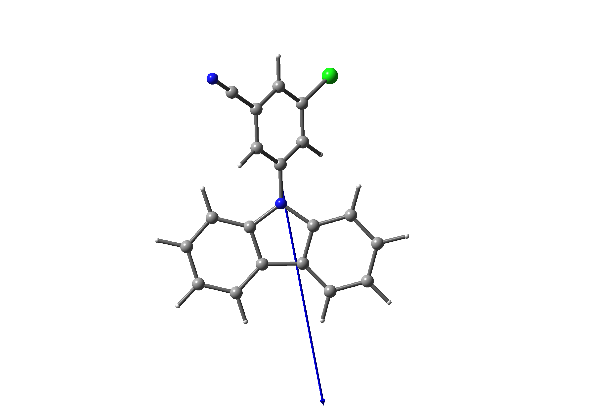  (-)15.0D |
| RTP-Br | 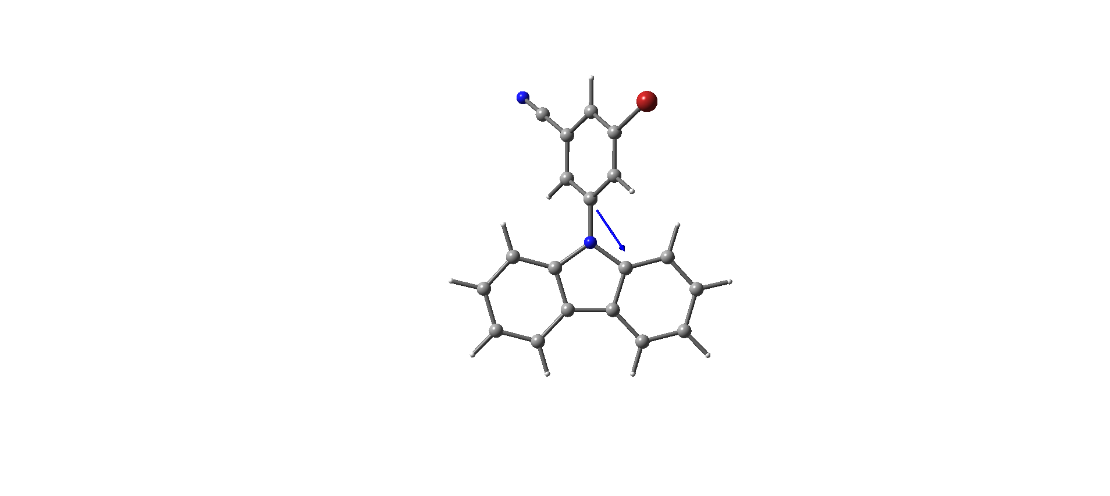  (-)3.5D | 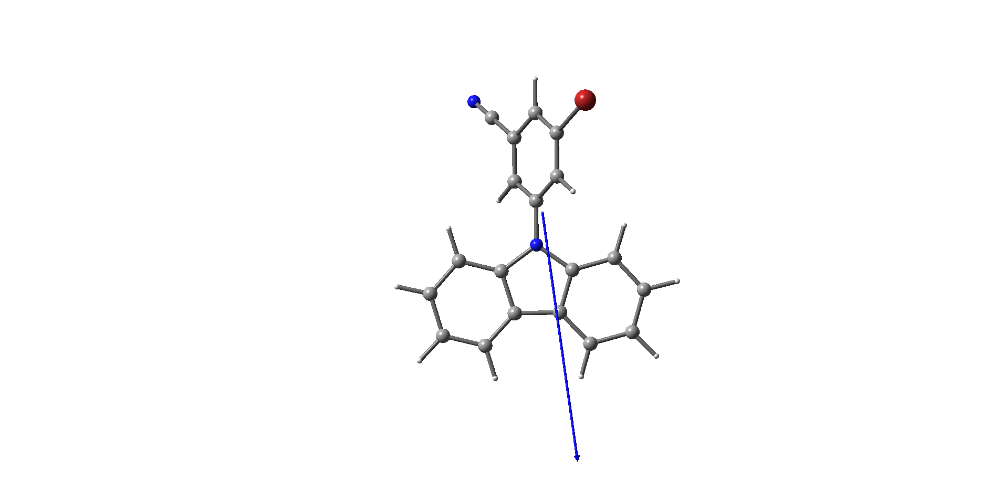  (-)15.1D |
| RTP-I | 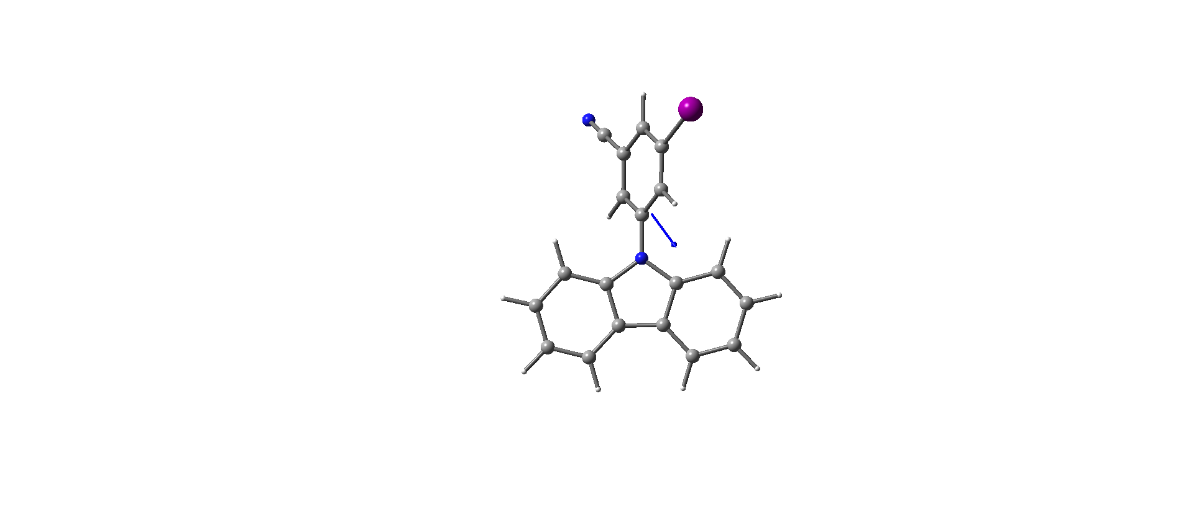  (-)3.6D | 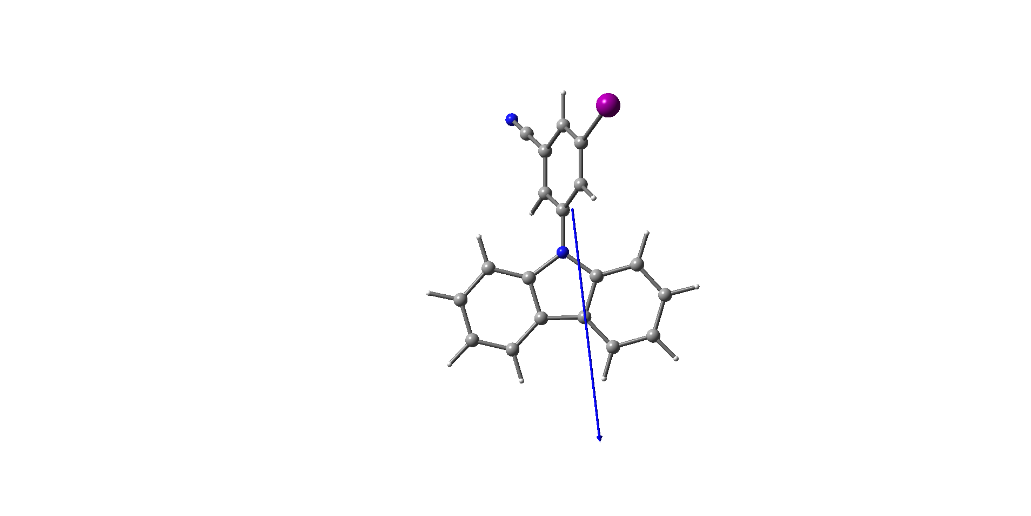  (-)14.9D |
| RTP-o-Br | 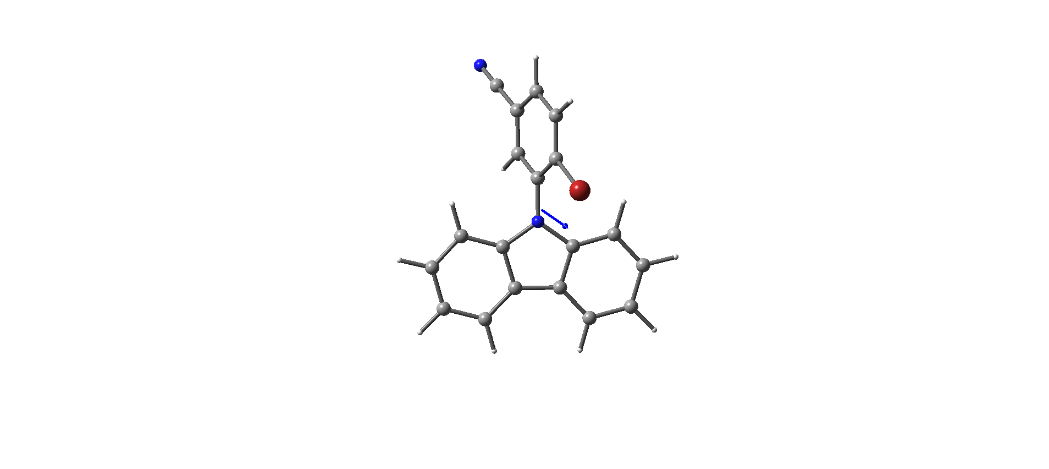  (-)3.0D | 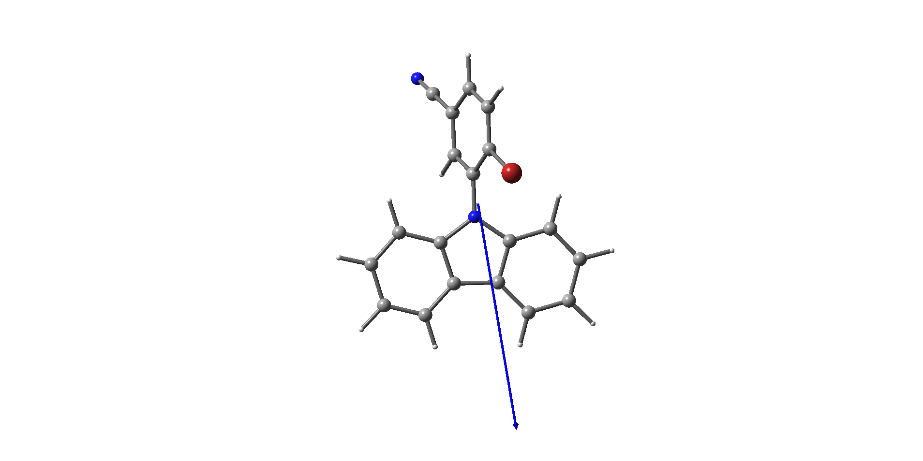  (-)14.0D |
| RTP-o-CN | 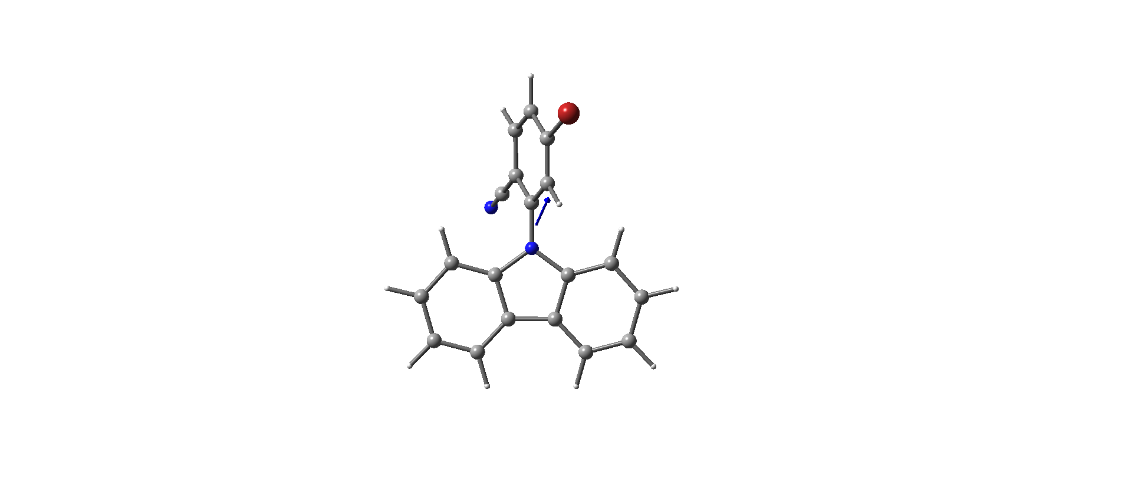  3.0D | 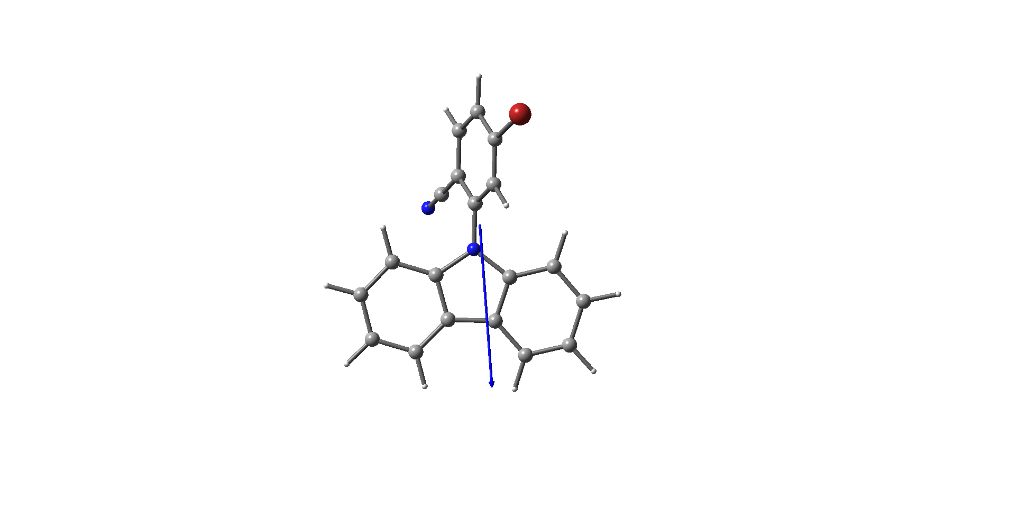  (-)9.5D |

**Table S8.** Orbital contributions to vertical excitations S_0_→S_1_ and oscillator strength coefficients for optimized geometries.

|  | Excitation energy | Character | Oscillator strength |
| --- | --- | --- | --- |
| RTP-Cl | S1 (^1^CT): 3.90eV = 318nm | HOMO – LUMO (96%) | 0.0541 |
|  | T1 (mix ^3^CT and ^3^LE(phenyl)): 3.47eV = 357nm |  |  |
|  | T2 (^3^LE(Cz)): 3.55eV = 350nm |  |  |
| RTP-Br | S1 (^1^CT): 3.89eV = 319nm | HOMO – LUMO (95%) | 0.0537 |
|  | T1 (mix ^3^CT and ^3^LE(phenyl)): 3.46eV = 358nm |  |  |
|  | T2 (^3^LE(Cz)): 3.55eV = 350nm |  |  |
| RTP-I | S1 (^1^CT): 3.88eV = 319nm | HOMO – LUMO (95%) | 0.0536 |
|  | T1 (mix ^3^CT and ^3^LE(phenyl)): 3.46eV = 358nm |  |  |
|  | T2 (^3^LE(Cz)): 3.55eV = 350nm |  |  |
| RTP-o-Br | S1 (^1^CT): 3.87eV = 320nm | HOMO – LUMO (97%) | 0.0355 |
|  | T1 (^3^LE(Cz)): 3.55eV = 349nm |  |  |
|  | T2 (mix ^3^CT and ^3^LE(phenyl)): 3.58eV = 346nm |  |  |
| RTP-o-CN | S1 (^1^CT): 3.75eV = 330nm | HOMO – LUMO (94%) | 0.0800 |
|  | T1 (mix ^3^CT and ^3^LE(phenyl)): 3.42eV = 362nm |  |  |
|  | T2 (^3^LE(Cz)): 3.55eV = 349nm |  |  |


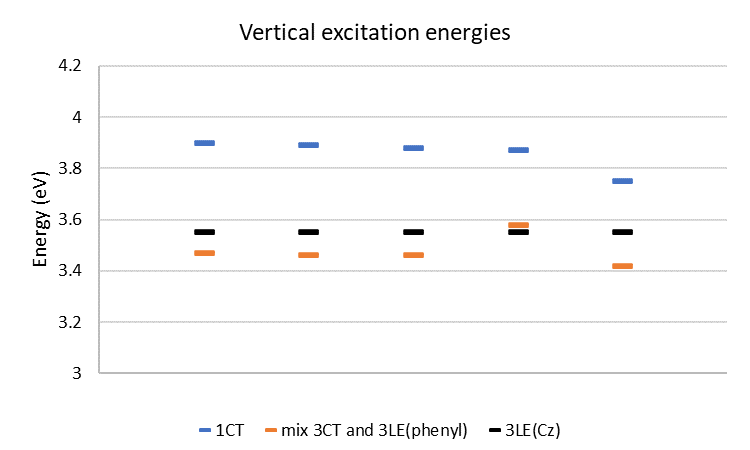


**RTP-Cl RTP-Br RTP-I RTP-o-Br RTP-o-CN**

**Table S9.** Natural transition orbitals (NTOs) of triplet excited states for optimized geometries.

|  |  | HONTO | LUNTO |
| --- | --- | --- | --- |
| RTP-Cl | T1 | 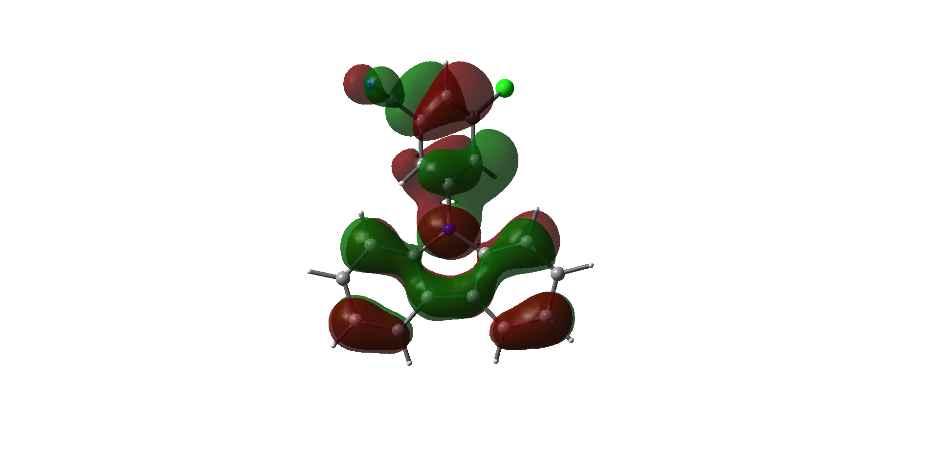 | 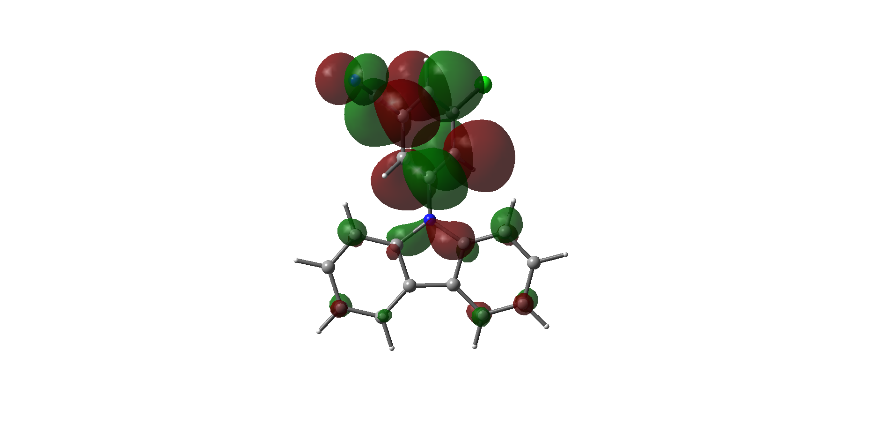 |
|  | T2 | 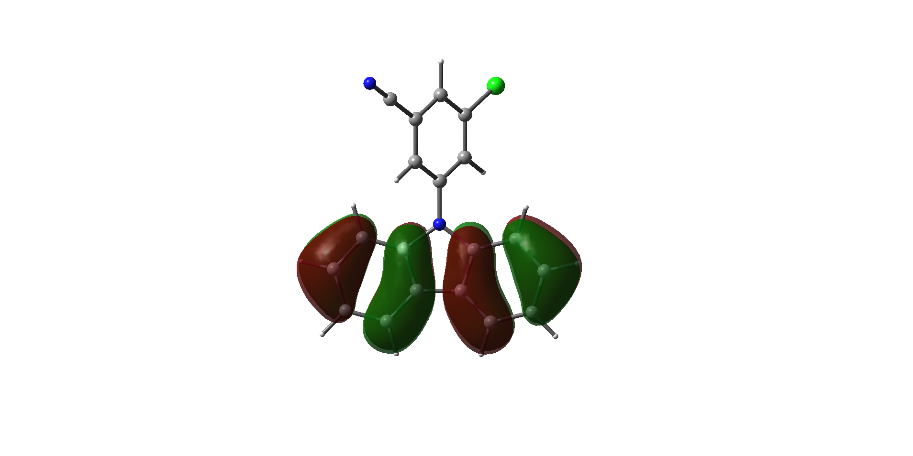 | 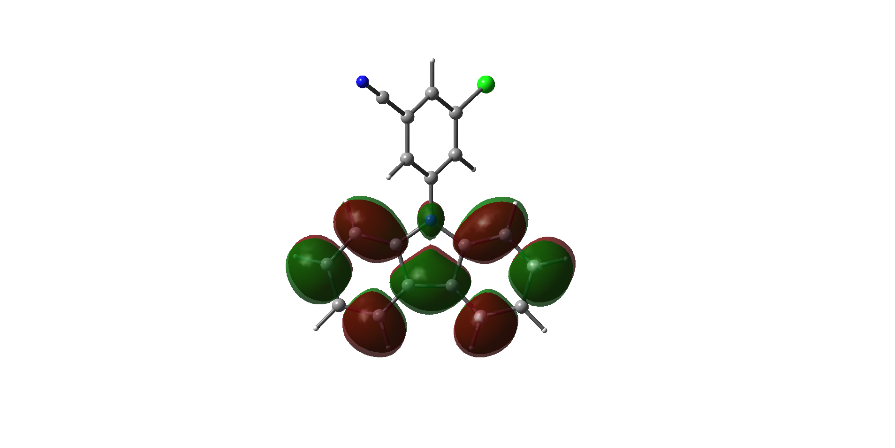 |
| RTP-Br | T1 | 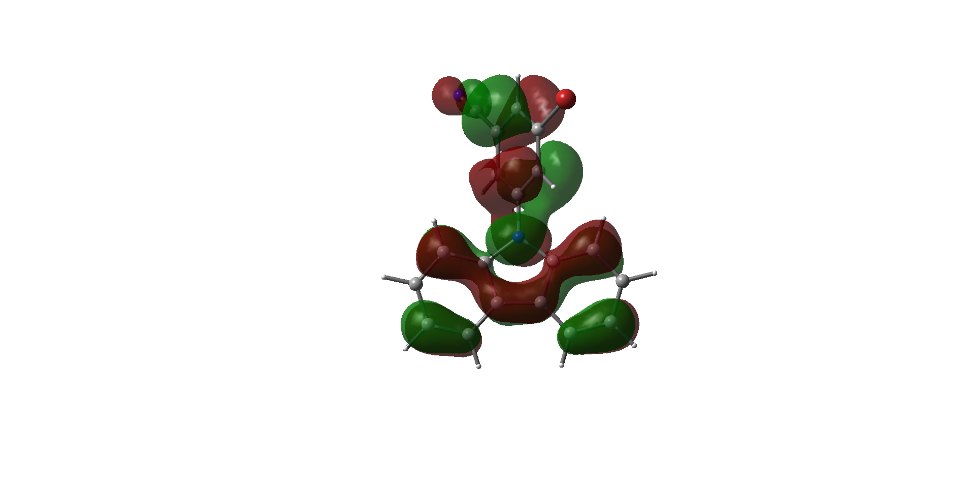 | 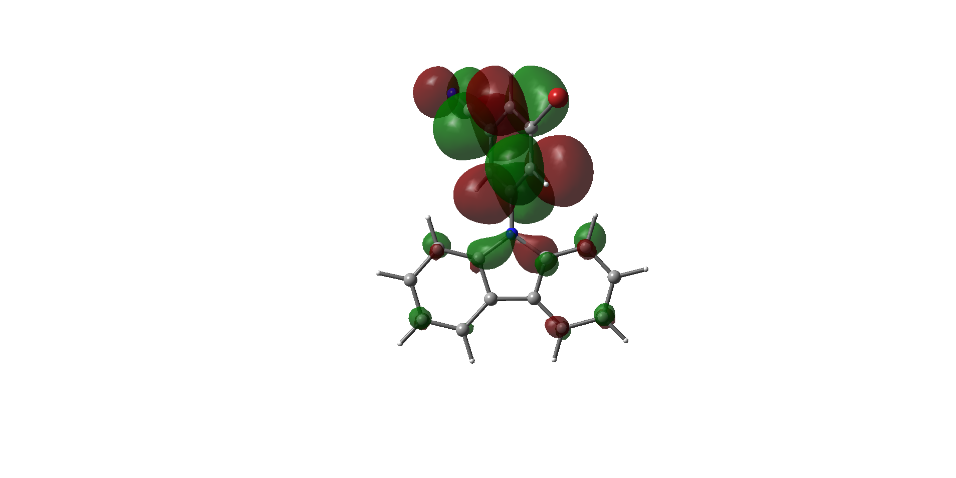 |
|  | T2 | 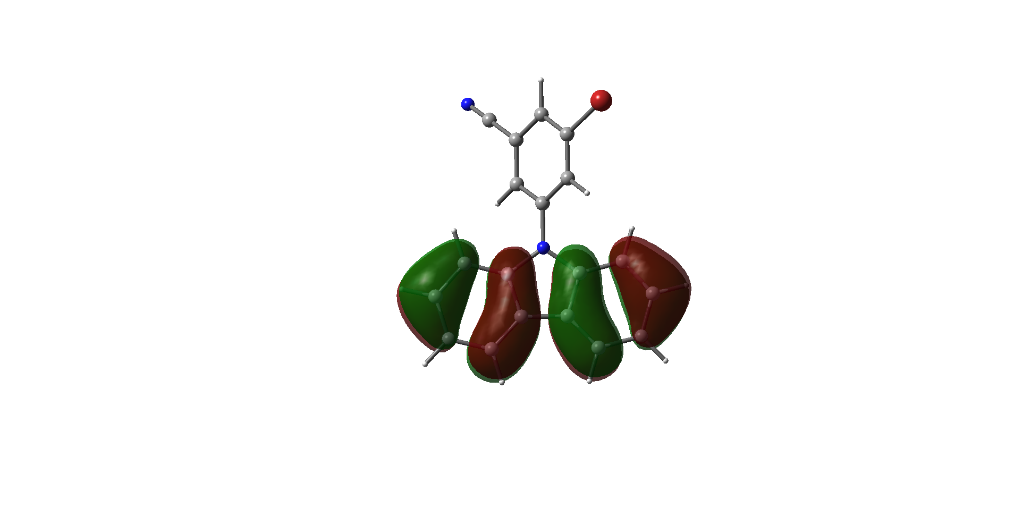 | 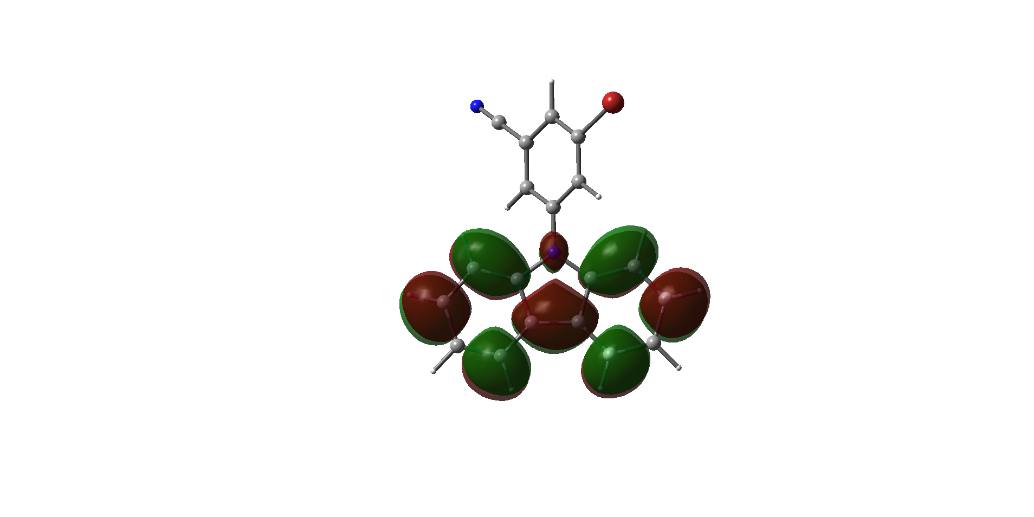 |
| RTP-I | T1 | 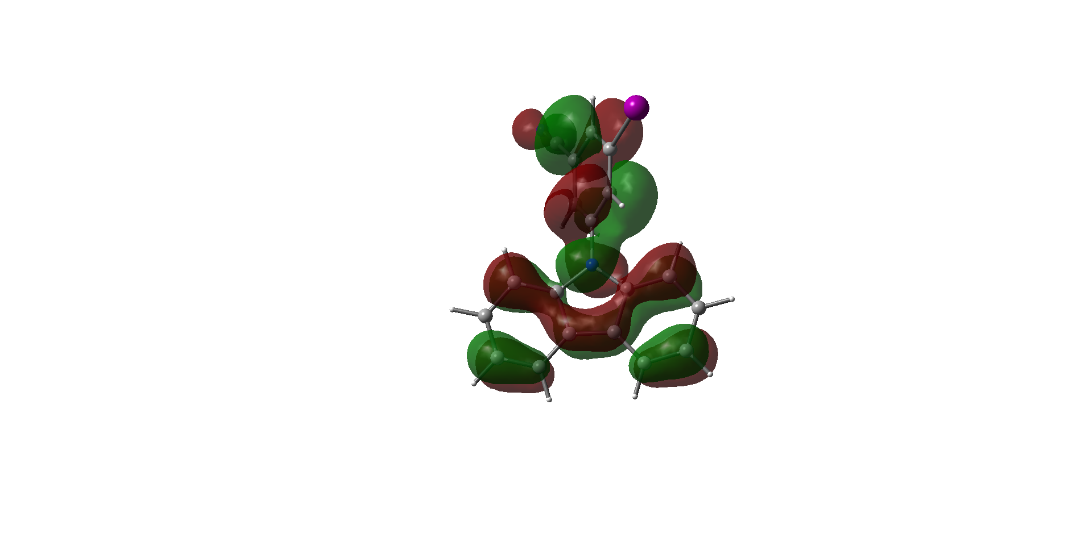 | 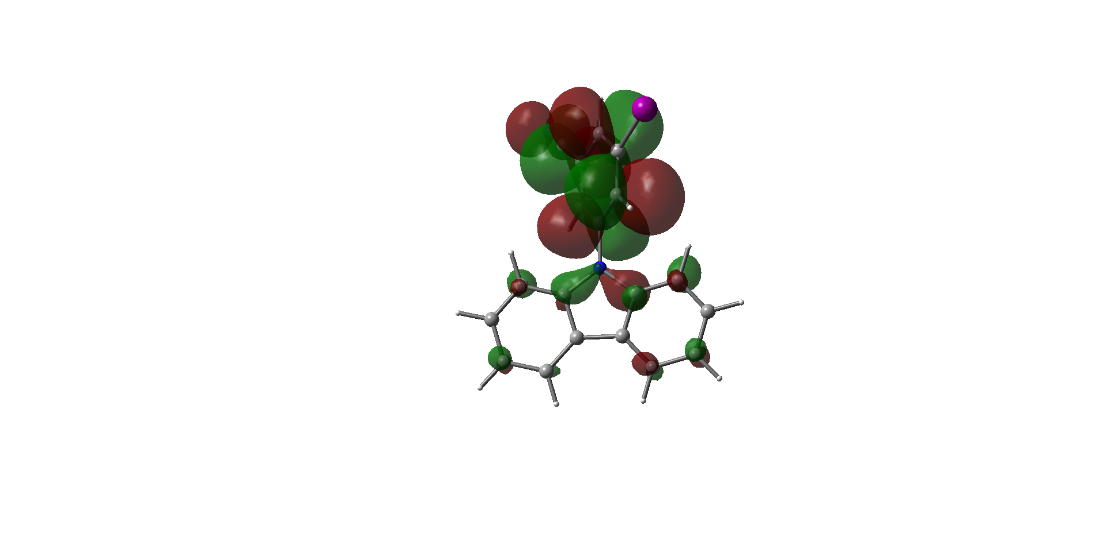 |
|  | T2 | 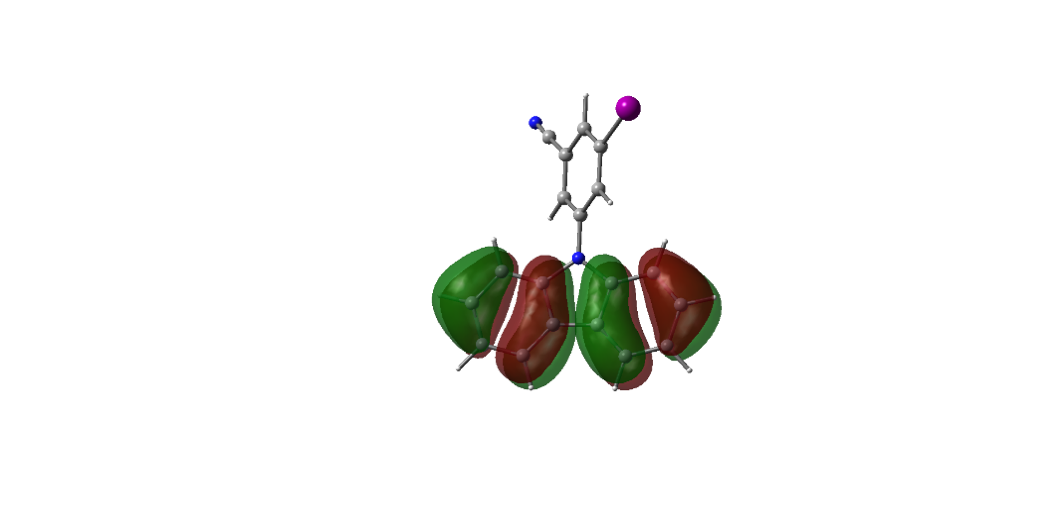 | 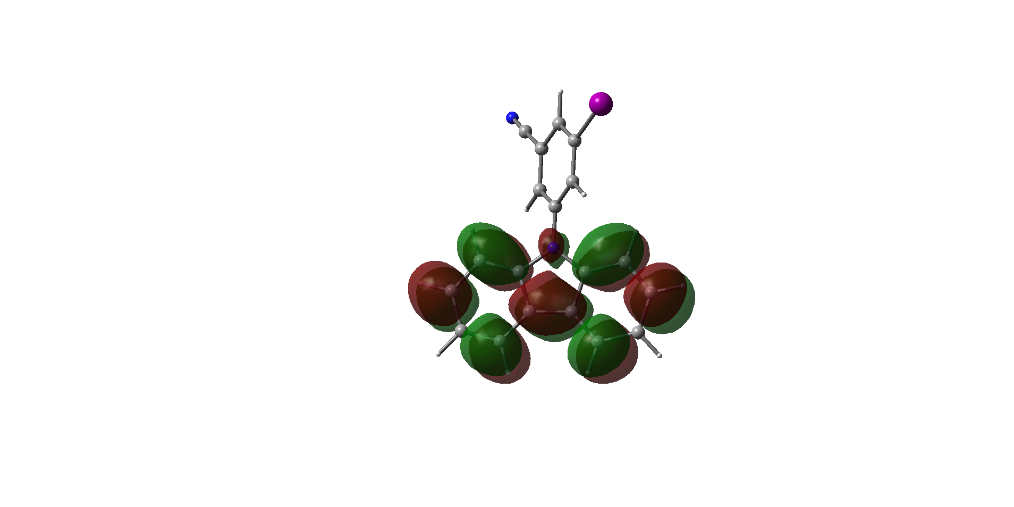 |
| RTP-o-Br | T1 | 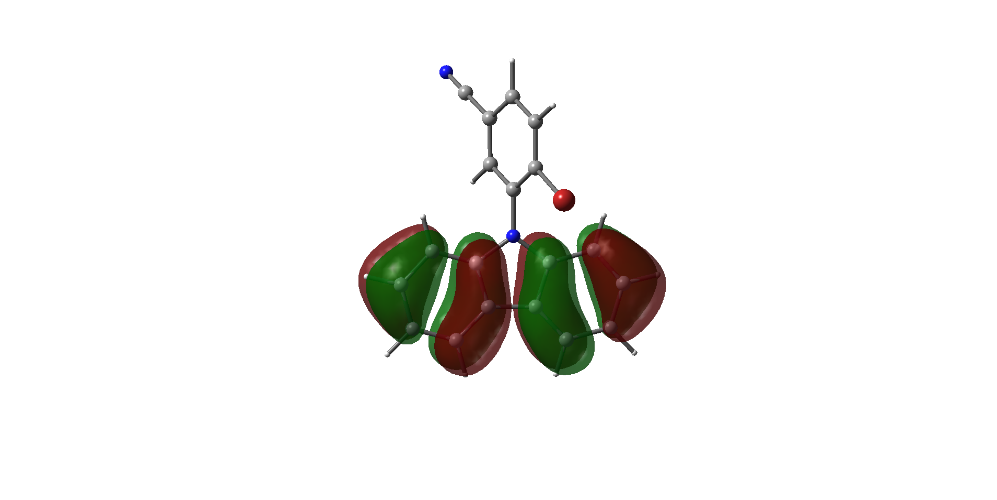 | 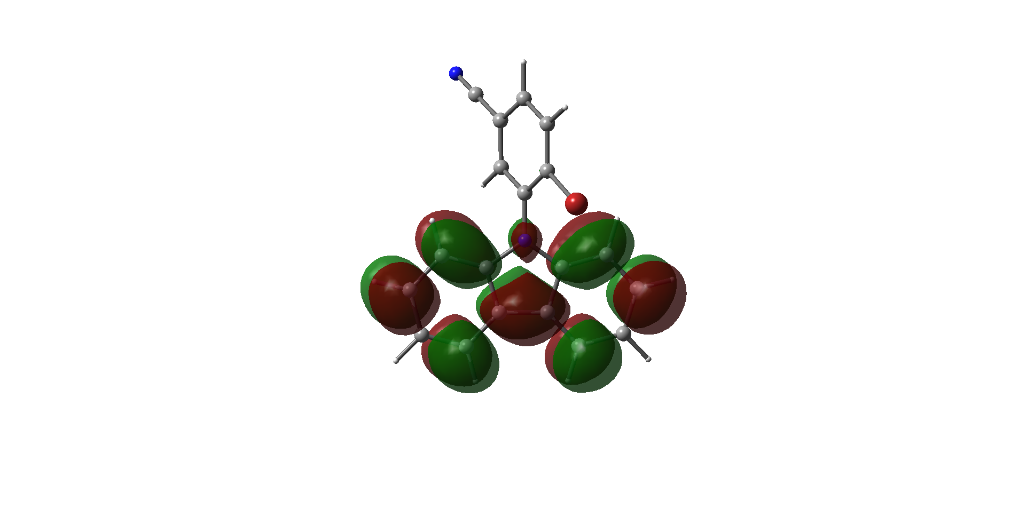 |
|  | T2 | 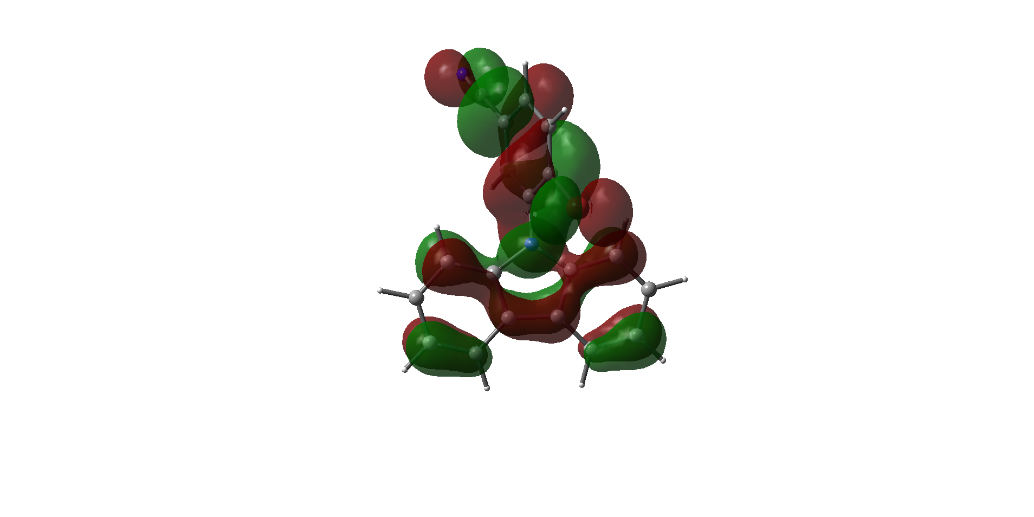 | 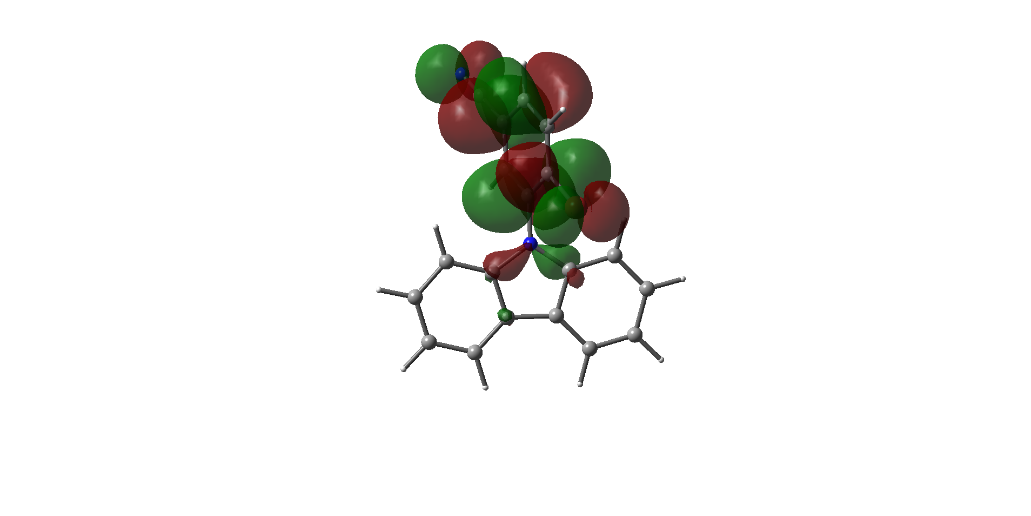 |
| RTP-o-CN | T1 | 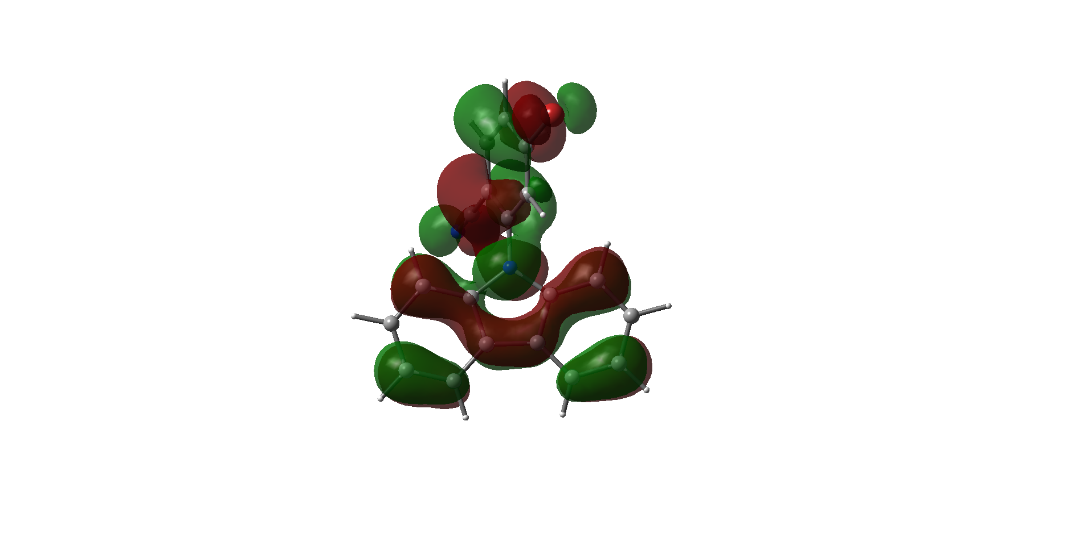 | 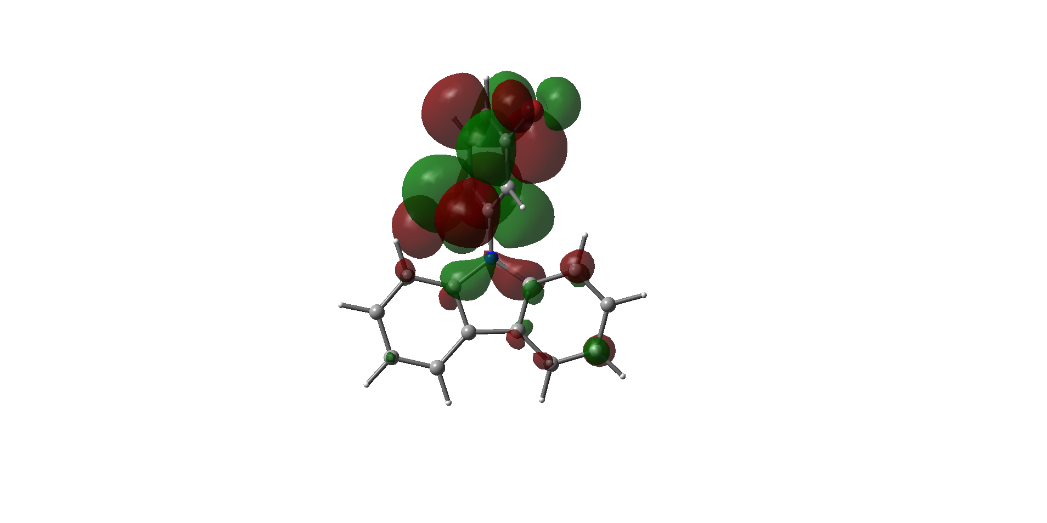 |
|  | T2 | 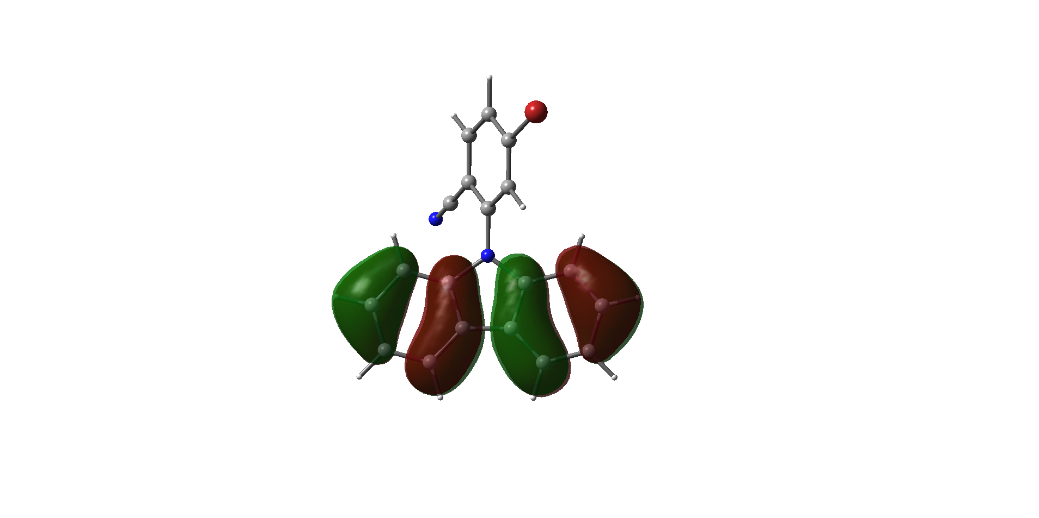 | 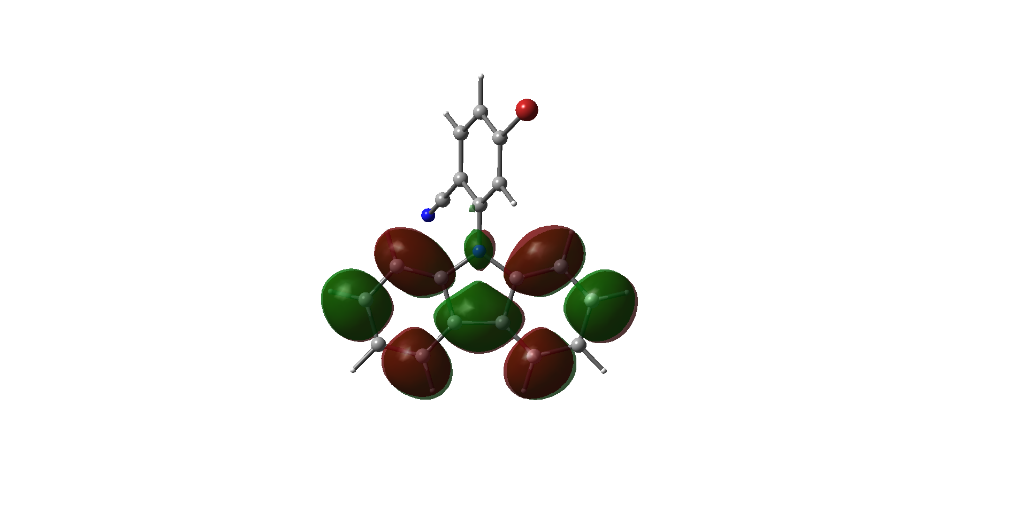 |
